# Identification of acute kidney injury after non-cardiac surgery by machine learning leveraging a large electronic health record database

**DOI:** 10.1038/s41598-026-65849-y

**Published:** 2026-08-18

**Authors:** Jonas Leins, Hendrik Meyer, Laurenz Engel, Christian Hinze, Hans-Jörg Gillmann, Thomas Stüber, Helena U. Zacharias

**Affiliations:** 1 Hannover Medical School, Peter L. Reichertz Institute for Medical Informatics of TU Braunschweig and Hannover Medical School, Hannover, 30625 Germany; 2 Hannover Medical School, Department of Anesthesiology and Intensive Care Medicine, Hannover, 30625 Germany; 3 Hannover Medical School, Department of Nephrology and Hypertension, Hannover, 30625 Germany

**Keywords:** Post-operative acute kidney injury, Machine learning, Electronic health record, Time-series data, Non-cardiac surgery, Feature importance, Cardiology, Diseases, Medical research, Nephrology, Risk factors

## Abstract

Post-operative acute kidney injury (PO-AKI) is a frequent complication after non-cardiac surgery, substantially increasing patients’ mortality and morbidity rates. Early identification of patients at high risk of PO-AKI could facilitate improved patient care. We investigated a large, single-center database of 13,890 patients undergoing non-cardiac surgery, of whom 1,718 (12.37*%*) developed PO-AKI. Based on 62 pre-, intra- and postoperative patient characteristics, including time-series mean arterial pressure measurements, we trained and validated eight different machine learning (ML) algorithms to predict PO-AKI. Top ML model performance based on Area under the Receiver Operating Characteristics curve (ROC-AUC) was achieved at 0.742 (95% CI, 0.725-0.762) on the validation data set by a Random Forest classifier. Feature Importance assessment revealed a wide range of patient characteristics predictive of PO-AKI, which, however, varied considerably between ensemble and non-ensemble ML models. Training and evaluation of Random Forest models at seven different perioperative time-points revealed a performance increase in ROC-AUC from 0.722 (95% CI, 0.703-0.746) preoperatively to 0.738 (95% CI, 0.716-0.755) postoperatively on the validation data set. In conclusion, we present a comprehensive performance evaluation of different ML algorithms for the personalized prediction of PO-AKI on a large, real-world electronic health record data set.

## Introduction

Post-operative acute kidney injury (PO-AKI) is a frequent and severe complication of both cardiac and non-cardiac surgery with an incidence rate of up to 39%^1,2^. PO-AKI is commonly defined as an increase in serum creatinine or a decrease in urine output that meets the existing Kidney Disease: Improving Global Outcomes (KDIGO) criteria for AKI within seven days after surgery^3^. It represents a severe clinical syndrome, leading to increased patient mortality and morbidity^4^. The causes are often complex and multifactorial, encompassing a wide range of both pre- and peri-operative factors, including advanced age, chronic comorbidities, prolonged surgery, and nephrotoxins^2,3,5,6^. Likewise, PO-AKI occurrence is markedly influenced by the type of surgical procedure, with incidence rates reaching 42% in cardiac surgeries and 29% in non-cardiac surgeries, respectively. The highest incidence rates for non-cardiac surgeries have been documented in abdominal surgeries^2,7^. The risk of PO-AKI can be effectively lowered by appropriate volume management, maintenance of adequate blood pressure, and the judicious avoidance of nephrotoxins following the KDIGO Clinical Practice Guideline for AKI^8^. Nevertheless, the implementation of personalized preventive measures in clinical settings remains limited. The timely identification of patients undergoing surgery with an elevated risk of PO-AKI could significantly improve patient care and subsequent outcomes, including most importantly lowered mortality and morbidity rates.

Machine Learning (ML) has already proven to be an extremely efficient tool to improve personalized risk prediction in clinical care^9^. Several ML-based models for the timely identification of patients at risk of PO-AKI have been developed, whereas the number of models specifically developed for the clinical setting of cardiac surgery significantly outweighs those specifically designed for non-cardiac surgery settings^5,10,11^. Many of these ML models, however, exhibit major shortcomings, which limit their clinical utility, including lack of generalizability of included predictor variables, employment of highly variable AKI definitions, model development on small patient cohorts with limited generalizability, e.g., restriction to only one type of non-cardiac surgery, and limited explainability and transparency of ML models hampering biomedical interpretation as well as user confidence and trust^12–14^. Additionally, many available ML models and traditional risk scores are solely based on pre-operative and static intraoperative risk factors and exclude readily available, intra- and post-operative, time-series patient monitoring parameters, which have already proven to play pivotal roles in the manifestation of PO-AKI^5,12,15^.

In this study, we systematically explored the predictive performance of eight interpretable ML algorithms to predict PO-AKI on a large, real-world, single-center database of 13,890 patients undergoing a wide range of different non-cardiac surgeries. To this end, we trained ML models in a development data set on up to 62 easily accessible, pre-, intra- and postoperative patient characteristics, including time-series Mean Arterial Pressure (MAP) values. We comprehensively assessed their performance on an internal validation data set and evaluated the impact of different patient characteristics on PO-AKI prediction by systematic Feature Importance analysis. Finally, we investigated how the predictive performance of the top scoring ML algorithm varied during the course of anesthesia depending on the amount of available predictor variables.

## Methods

### Data assessment

Ethical approval for this retrospective, single-center study was provided by the local Ethics Committee of Hannover Medical School (MHH), Germany, on June, the 11th, 2020 (approval number: 9168_BO-K_2020) and it was conducted in compliance with the Declaration of Helsinki and the World Medical Association. Due to the retrospective nature of the study, the need to obtain informed consent was waived by the Ethics Committee of MHH. The study includes adult patients at least 18 years of age, who underwent non-cardiac surgery with intubation at MHH, a tertiary referral university hospital in Germany, between 3/2014 and 9/2019. The patients had electronically available pre- and postoperative serum creatinine data, and no selection was made based on ethnicity. Patients with documented chronic renal insufficiency or chronic renal replacement therapy, as recorded in the electronic anesthesia information system, were excluded from the study. A detailed list of exclusion criteria is provided in Supplementary File S1 and the corresponding overview of the study population selection process is displayed in Supplementary Figure S1.

Pre-, intra-, and postoperative anesthesia data was documented using the ANDOK Anesthesia Information Management System and mainly derived from the preoperative anesthesia anamnesis (Supplementary File S2). All variables were grouped into eight different categories, including demography, preoperative risk assessment, pre-existing conditions, surgical department, durations of the anesthesia process, long-term medication, anesthesia medication, and MAP features. The primary endpoint of the study was postoperative acute kidney injury (PO-AKI) as defined by an increase in serum creatinine of *\ge* 0.3mg/dl or *\ge* 50% of the last pre-operative value within seven days post-operatively according to the KDIGO Clinical Practice Guideline for Acute Kidney Injury 2012^8^. After data merging, inclusion and exclusion criteria were validated, and data was subsequently anonymized. MAP time series data was analyzed to yield univariate representations of curve characteristics and enable meaningful ML analyses. In total, 65 threshold-based MAP characteristics were extracted, alongside six additional features, representing the dynamics of the MAP time series, similar to Xue et al.^16^. More details are provided in Supplementary File S3.

### Statistical analysis

Statistical and ML analyses were performed in *R*, versions 4.2.2 and 4.4.1, and Python, version 3.11.3^17,18^. Data cleaning was carried out employing the *R* packages *openxlsx*, *dplyr*, *tidyr*, *ggplot2*, *zoo*, *reshape2*, *moments*, and *trend*, and using the Python packages *pandas* and *numpy*^19–28^. For descriptive analyses, mean and standard deviation are reported for continuous variables, number of instances and percentage for categorical variables, respectively. Standardized mean differences (SMDs) were calculated using the *R* package *tableone* to assess the balance of baseline characteristics independent of sample size^29^. Groups were compared with logistic regression analysis using the *statsmodels* module, and *p*-values were adjusted using the Bonferroni correction with the *multipletests* function of this *Python* module^30^. Multicollinearity analyses, to identify strongly correlated features within the data set, were conducted by employing Pearson’s correlation coefficient utilizing the *R* package *Hmisc* for individual combinations of continuous features; for individual combinations of binary features, the Phi coefficient was calculated using the *R* package *psych*; individual combinations of binary and multinomial features were analyzed by calculating Kendall’s *τ*, employing the *R* package *stats*; correlations between continuous and binary and continuous and multinomial features were investigated using point biserial and multiserial correlations, respectively, calculated with the *R* package *psych*^31–33^. Visualisations of correlation matrices were carried out with the *R* package *ggcorrplot*^34^. For each correlated cluster a single feature was chosen to remain within the data set, as shown and detailed in Supplementary Figure S2 and File S4.

Prior to ML analyses, multinomial categorical features were binarized (occurrence vs. non-occurrence) in training and evaluation data sets for logistic Least Absolute Shrinkage and Selection Operator (LASSO), Ridge, and Elastic Net regression, as well as Support Vector Machine classifiers and linear extreme Gradient Boosting Machines, since they are not able to handle multinomial categorical variables. Continuous features were log$_2$-transformed and standardized to enable appropriate scaling of coefficients for linear classifiers.

### Machine learning

In total, eight binary classifiers were trained and evaluated: four regression based algorithms, including logistic Least Absolute Shrinkage and Selection Operator (LASSO)^35,36^ regression, logistic Group-LASSO regression^37^, logistic Ridge regression^38^, logistic Elastic Net regression^35^, binary Support Vector Machine (SVM) classifier^39^, and three ensemble methods, including binary Random Forest classifier (RFC)^40^, and linear as well as tree-based extreme Gradient Boosting Machine (xGBM)^41,42^.

#### Logistic LASSO, Group LASSO, Ridge, and Elastic Net regression

The logistic LASSO, Group LASSO, Ridge, and Elastic Net regression algorithms are penalized linear classifiers, each augmented with a specific regularization term to control the bias-variance trade-off^36,38,43^. Both the LASSO and Group LASSO algorithm employ an $L_1$ regularization term, which allows the direct selection of predictive features during model training by shrinking certain feature coefficients exactly to zero^35^. The Ridge regression algorithm uses an $L_2$ regularization term in order to shrink feature coefficients towards zero, which causes models to more effectively handle multicollinearity within the data set while simultaneously avoiding the formation of sparse matrices^38^. The Elastic Net algorithm includes a linear combination of an $L_1$ and $L_2$ regularization term, whereas the latter emphasizes on concurrently shrinking feature coefficients of strongly correlated groups of features^43^. The Group LASSO algorithm additionally facilitates the simultaneous coefficient shrinkage for non-orthogonal features belonging to a user-defined group, ensuring equal coefficient regularization for all levels of multinomial features^37^. Logistic LASSO, Ridge, and Elastic Net regression were implemented using the *R* package *glmnet*, the logistic Group LASSO regression, where one-hot encoded multinomial variables were assigned to respective groups to account for their original multinomial character, was implemented employing the *R* package *gglasso*^44,45^. For each model, the regularization hyperparameter *λ* was optimized with respect to the misclassification error within a five-fold internal cross-validation. For the Elastic Net regression model, the alpha parameter, controlling the impact of $L_1$ and $L_2$ regularization, was set to 0.5, equalizing the impact of both regularization terms.

#### Random Forest classifier

 An RFC encompasses a multitude of decision trees, trained on different bootstrap samples of the training data^40^. Thus, it combines the interpretability of decision trees with the improved robustness against overfitting of ensemble learning methods. Labels for new data points are assigned via a majority vote over all individual tree-predictions in the forest. We used the *R* packages *ranger* and *caret* to train RFCs^46,47^. Optimal forest depth was determined by comparing the performance of models with differing forest depths, ranging from 25 to 1,500, in an outer five-fold cross-validation on the development data set. For each of these models, the number of sampled features per branch-split and the minimal size of leaf nodes were optimized in an inner five-fold cross-validation on the training data sets from the outer cross-validation.

#### Support Vector Machine classifier

 SVM classifiers are an instance of maximum margin classifiers, as they aim to construct a hyperplane separating the two classes within a high-dimensional feature space by maximizing the minimal distance of both classes to the hyperplane^39^. To achieve this, usually a kernel function is used to transform the feature space into a higher dimension, where a linear separation of the two classes is possible, and subsequently, the resulting decision boundary is projected back into the original feature space^39,48^. For data set classification, we trained SVMs using a radial basis function kernel employing the *R* packages *kernlab* and *caret*^47,49,50^. The hyperparameter *σ*, controlling the width of the kernel, and the cost parameter *C*, controlling the trade-off between margin width and misclassification error, were optimized in a five-fold cross-validation on the development data set. An automated grid search provided by the *caret* package with a maximum number of 20 unique combinations was used to choose parameter ranges for tuning^47^.

#### Linear and tree-based extreme gradient boosting machine

 Similar to RFCs, GBMs provide an approach for combining multiple weaker ML models, also called base learners, like logistic regression or decision tree models, to create a more stable ensemble model^41^. In contrast to the parallel combination of weak learners in RFCs, GBMs implement multiple learners sequentially, while each learner changes the impact of input features for the training process to compensate for misclassifications of the previous learners. Extreme Gradient Boosting Machines (xGBMs) represent a specifically efficient, scalable, and scarcity aware implementation of Gradient Boosting algorithms^42,51^. We implemented xGBMs using both linear logistic regression models and decision trees as a base learner, respectively, employing the *R* packages *caret* and *xgboost*^42,47^. Hyperparameters for both xGBM algorithms were tuned in a five-fold cross-validation on the development data set. For both algorithms, the number of boosting rounds was chosen from a set of $\{$50, 100, 200, 300*\}* rounds and the learning rate was chosen from $\{$0.1, 0.01*\}*. For the linear regression based xGBM, both $L_1$ and $L_2$ regularization parameters were chosen from a sequence from 0.1 to 1 in six equidistant steps. For the tree-based xGBM algorithm, maximum tree depth was tuned from a range of $\{$10, 15, 25*\}* branch nodes, and the subsample ratio of features for tree construction was chosen from a range between 0.4 and 0.95 in 9 equidistant steps. Minimum loss reduction was set to zero, minimum sum of hessian instance weight and subsample ratio of training instances were set to one, respectively.

#### Model training and performance assessment

 First, the complete data set was split into a development and a validation data set by random sampling within the levels of the binarized response variable PO-AKI to ensure the same class distribution among both data sets. Subsequently, the influence of imbalanced class distribution of the PO-AKI response variable was investigated in an internal resampling approach using the “original development data set” and a randomly undersampled development data set, termed “balanced development data set”, ensuring a balanced class distribution in the latter. Final models were then trained on the best performing development data set and internally validated in a bootstrapping approach of 100 bootstrap replicates on the validation data set, see Supplementary Figure S1. ML model performance measures included Area under the Receiver Operating Characteristics curve (ROC-AUC), Balanced Accuracy (BACC), sensitivity, and specificity, calculated employing the *R* package *tidymodels*^52^. Additionally, positive and negative predictive values, precision, recall, F1-score, detection rate, and detection prevalence were computed using the *R* package *caret*^47^. Moreover, ROC and Precision Recall (PR) curves were generated employing the *R* package *ggplot2*^22^. Following model performance assessment, a Decision Curve Analysis (DCA) was performed for all decision thresholds of the best performing model, following Vickers et al., to investigate potential clinical benefit^53^. Additionally, a calibration curve was calculated for the best performing model, alongside the respective calibration slope, intercept and Brier score, in order to assess the translation of risk probabilities^54^. Training of all ML algorithms was carried out on the MHH High-Performance Computing (HPC) infrastructure.

#### Feature importance assessment

 Native Feature Importances (FI) of different ML models were computed using the *R* package *caret* for all algorithms except for logistic regressions^47^. Additionally, the Shapley Additive Explanations (SHAP) implementation *kernelshap* was used to compute contributions of all features to each prediction on the validation data set^55^. All FI scores and Shapley values were scaled to their respective maximum value for easier comparison. A detailed account on native FI extraction and Shapley value computation is provided in Supplementary File S5.

#### Sensitivity analysis

To investigate the influence of specific AKI severity stages on the importance assigned to individual predictors, RFC models were trained and evaluated using subsets of the full training and development data set. These individual data subsets retained only the non-occurrence cases alongside those that showed the respective outcome AKI severity stage (AKI stage 1, 2, or 3). Subsequently, model performance as well as native and post-hoc feature importances were assessed similar to the full model training and evaluation.

#### Prediction of PO-AKI in the perioperative course

 To investigate whether the predictive performances of our PO-AKI ML classifiers vary during the course of anesthesia, RFC models were trained and evaluated at eight different time points during the perioperative course. RFC training followed the methodology described above with an extension to different perioperative time points. These time points included the preoperative time point (preoperative), the end of induction (end of induction), five intraoperative time points in 30-minute intervals and the postoperative time point (postoperative). A separate RFC model was trained for each of these time points. The underlying data sets only contained the variables available up to this point in time, so that the postoperative predictor variable set was identical to the complete predictor variable set for the RFC algorithm described in the previous section. Each model represents a distinct prediction setting corresponding to a specific perioperative stage and is based exclusively on the information available up to that time point, allowing risk estimation to be updated throughout the perioperative course from preoperative assessment to intraoperative reassessment and early postoperative evaluation. Potential predictor variables of the various categories were selected as described above. For each intraoperative time point, the characteristics of the MAP time series for the corresponding interval were also included. The number of predictor variables per time point was 39 variables preoperatively, 58 variables intraoperatively, and 62 variables postoperatively, respectively.

The manuscript was prepared following the STROBE guideline and the TRIPOD+AI guideline for clinical prediction algorithms.

## Results

### Baseline characteristics of study population

From March, the 1st, 2014 to September, the 30st, 2019, a total of 178,531 non-cardiac surgical procedures were documented at Hannover Medical School. Of these, 133,116 operations met the inclusion criteria of the study protocol, and a declaration of consent for inclusion in the study was available for 111,345 of these operations. After applying exclusion and quality criteria as well as a data cleansing process, which also included the exclusion of repetitive operations of individual patients, the study population totaled 13,890 patients (Supplementary Figure S1). This population was divided into a development cohort, termed “original development cohort”, (*n* = 9,260) and a validation cohort (*n* = 4,630), respectively. The final balanced development cohort included 2,286 patients. A comparison between the complete study cohort and the final study cohort is provided in Supplementary Table S1. Based on an absolute SMD > 0.1, the final study cohort comprised slightly older patients, a higher proportion of patients with Revised Cardiac Risk Index (RCRI) 1 and American Society of Anesthesiologists (ASA) class III, fewer patients with RCRI 0 and ASA classes I and IV, longer induction and anesthesia durations, and a higher proportion of general and neurosurgical procedures than the complete study cohort.

The incidence of postoperative acute kidney injury (PO-AKI) was 12.34% (1,143/9,260) in the original development cohort, 50.00% (1,143/2,286) in the balanced development cohort, and 12.42% (575/4,630) in the validation cohort, respectively. In the balanced development cohort, 55.20% of patients were male, the mean age was 59.72 ± 16.50 years, and the mean preoperative estimated Glomerular Filtration Rate (eGFR) was 80.72 ± 27.48 mL/min/1.73 m$^{2}$. In the validation cohort, 53.80% of patients were male, the mean age was 58.68 ± 17.30 years, and the mean preoperative eGFR was 84.71 ± 24.74 mL/min/1.73 m$^{2}$.

Multicorrelation analyses amongst the baseline characteristics of the original development cohort resulted in the identification of 17 strong (correlation coefficients > 0.60) associations between potential PO-AKI predictor variables (Supplementary Figure S2). These associations included, e.g., duration of surgery and anesthesia, height and sex, categorized and binarized diabetes prevalence variables, or weight and Body Mass Index (BMI), respectively. Consequently, subsequent statistical and ML analyses were carried out including only a subset of 62 predictor variables after exclusion of highly correlated patient characteristics, as detailed in Supplementary File S2, to avoid multicollinearity. All 62 predictor variables and corresponding summary statistics in the balanced development as well as in the validation cohort are listed in Supplementary Tables S2 and S3.

### Univariate association of risk factors with PO-AKI

Univariate analyses showed significant differences with Bonferroni adjusted p-values < 0.001 between patients with and without PO-AKI in the balanced development cohort and the validation cohort (Supplementary Tables S2 and S3), respectively. Demographic variables such as age (*β* = 0.015 in the balanced development cohort; *β* = 0.020 in the validation cohort) were consistently associated with a higher risk of PO-AKI. A higher preoperative eGFR (*β* = -0.015 in the balanced development cohort; *β* = -0.018 in the validation cohort) was associated with a lower risk of PO-AKI. Prevalent liver cirrhosis was significantly associated with PO-AKI in both cohorts (*β* = 1.409 in the balanced development cohort; *β* = 1.381 in the validation cohort). PO-AKI occurred significantly less for neurosurgical procedures in both cohorts. In contrast, there was a significantly increased risk of PO-AKI for urological procedures in the balanced development cohort (*β* = 0.903) and for general surgical procedures in the validation cohort (*β* = 0.563). With regard to long-term medication, significant positive associations between PO-AKI and the administration of diuretics (*β* = 0.947 in the balanced development cohort; *β* = 0.699 in the validation cohort) were found.

### Development and performance assessment of machine learning models for individual PO-AKI risk prediction

A resampling approach was used to compare the impact of balanced and unbalanced outcome class distributions in the development data set on ML model performance (Supplementary File S6 and Supplementary Table S4). ML models trained on subsets of the balanced development data set considerably outperformed those trained on subsets of the unbalanced development data set. Accordingly, development of the final prediction models was carried out exclusively on the balanced development data set, leveraging its full sample size.

Variance estimation of the performance assessment of final models in the validation cohort was carried out by a bootstrapping approach with 100 bootstrap replicates. Average performances of all final PO-AKI classification models are shown in Table 1 and Supplementary Figure S3. ROC-AUCs ranged from 0.704 (95% confidence interval, CI, 0.680 to 0.726) to 0.742 (95% CI, 0.725 to 0.762) for linear xGBM and the RFC, respectively. The RFC model trained on the entire balanced development data set yielded the best performance with a BACC of 0.669 (95% CI, 0.648 to 0.691) and an F1-score of 0.336 (95% CI, 0.310 to 0.369). The worst performance according to ROC-AUC was achieved by Linear xGBM with a corresponding BACC of 0.654 (95% CI, 0.632 to 0.672) and an F1-score of 0.320 (95% CI, 0.292 to 0.346). The decision curve analysis performed on the best performing RFC prediction model showed a slight increase in net benefit when using the model for threshold probabilities between 0.7 and 0.12, falling below zero when exceeding a threshold of 0.124 (Supplementary Figure S4).

**Table 1:** Final model performances of eight different machine learning algorithms. Mean and standard deviation of different performance metrics, estimated on 100 bootstrap replicates of the validation data set, using final classification models trained on the complete balanced development cohort to estimate overall performance variance are given. A classification threshold of 0.5 was used to evaluate sensitivity and specificity measures. Abbrev.: BACC, Balanced Accuracy; LASSO, Least Absolute Shrinkage and Selection Operator; Neg Pred Value, negative predictive value; RFC, Random Forest Classifier; ROC-AUC, area under receiver operating characteristics curve; SVM, Support Vector Machine; xGBM, extreme Gradient Boosting Machine.

Model	ROC-AUC	Sensitivity	Specificity	Neg Pred Value	Precision	F1-score	BACC
Group LASSO	0.726 ± 0.011	0.644 ± 0.018	0.675 ± 0.008	0.930 ± 0.005	0.219 ± 0.010	0.327 ± 0.012	0.659 ± 0.010
LASSO	0.722 ± 0.011	0.639 ± 0.019	0.673 ± 0.007	0.929 ± 0.005	0.217 ± 0.008	0.324 ± 0.011	0.656 ± 0.010
Ridge	0.725 ± 0.012	0.651 ± 0.021	0.679 ± 0.008	0.931 ± 0.005	0.224 ± 0.011	0.333 ± 0.014	0.665 ± 0.011
Elastic Net	0.723 ± 0.012	0.643 ± 0.020	0.678 ± 0.008	0.931 ± 0.005	0.220 ± 0.010	0.328 ± 0.013	0.661 ± 0.011
SVM	0.733 ± 0.010	0.645 ± 0.018	0.681 ± 0.007	0.931 ± 0.004	0.223 ± 0.010	0.332 ± 0.012	0.663 ± 0.010
Linear xGBM	0.704 ± 0.012	0.650 ± 0.021	0.658 ± 0.007	0.930 ± 0.004	0.212 ± 0.011	0.320 ± 0.014	0.654 ± 0.011
Tree xGBM	0.731 ± 0.011	0.657 ± 0.020	0.666 ± 0.007	0.932 ± 0.005	0.218 ± 0.010	0.327 ± 0.013	0.661 ± 0.011
RFC	0.742 ± 0.010	0.655 ± 0.021	0.683 ± 0.007	0.933 ± 0.004	0.226 ± 0.012	0.336 ± 0.015	0.669 ± 0.011

Assessment of a calibration curve generated on the respective validation data set and calculation of associated measures, performed in 100 bootstrap replications on the validation data set, resulted in a Brier Score of 0.208 (95% CI, 0.205 to 0.212), alongside a calibration slope of 1.307 (95% CI, 1.196 to 1.442) and a calibration intercept of -1.953 (95% CI, -2.054 to -1.871). Both nonparametric and logistic calibration curves lie consistently below the diagonal (Supplementary Figure S5). The top-performing RFC trained on the full perioperative feature set is implemented as an online web service tool freely available at [https://post-operative-aki.ckdn.app] and as a GitHub resource at [https://github.com/JoLeins/risk-calculator-PO-AKI]. All analytical and development code used in this study is freely available within the same repository.

**Fig. 1:**
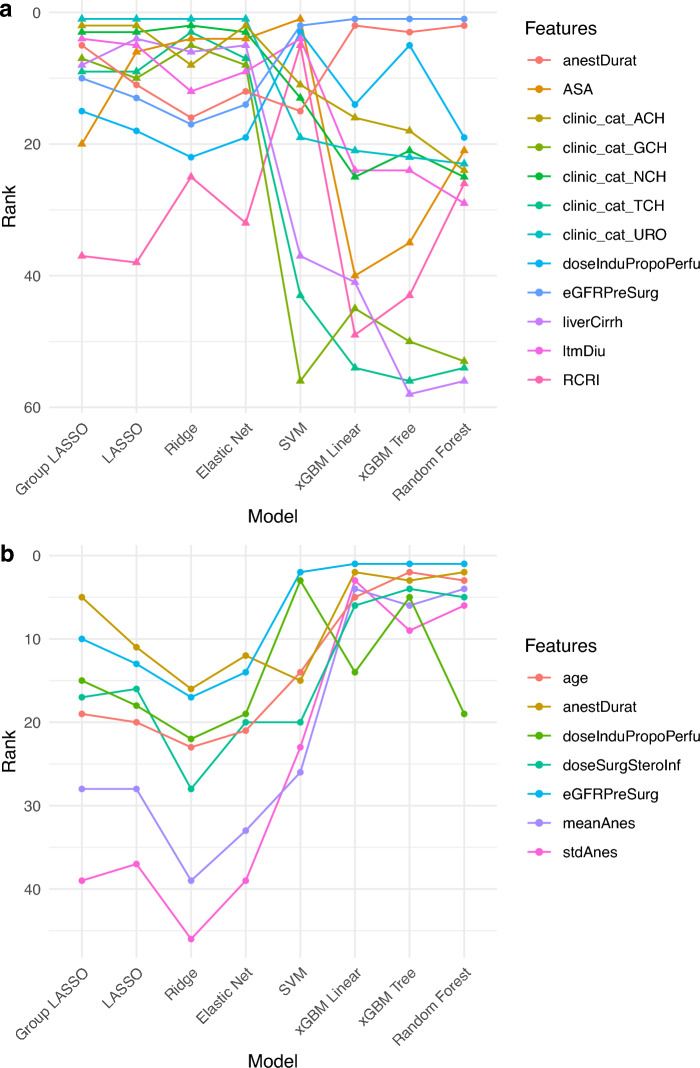
Ranks of native Feature Importance (FI) assigned by different PO-AKI prediction models trained on the complete balanced development cohort. The FI ranks across all models are displayed for the most important features (ranks 1-5) in each non-ensemble (**a**) and ensemble model (**b**). Predictors were ranked based on the absolute native FI that was estimated by the respective model and scaled to the largest importance per model. Lower rank values indicate higher importance with rank 1 representing highest importance. Continuous features are shown as dots, categorical features are displayed as triangles, respectively. Abbrev.: age, patient age; anestDurat, duration of anesthesia; ASA, American Society of Anesthesiology physical status; clinic_cat_ACH, general surgery; clinic_cat_GCH, vascular surgery; clinic_cat_NCH, neurology surgery; clinic_cat_TCH, thoracic surgery; clinic_cat_URO, urology surgery; doseInduPropoPerfusor, dose of propofol administered via perfusor during induction; doseSurgSteroInf, dose of sterofundin administered via infusion during induction; eGFRPreSurg, estimated glomerular filtration rate before surgery; liverCirrh, liver cirrhosis; ltmDiu, longterm medication with diuretics; meanAnes, mean of MAP during anesthesia; RCRI, Revised Cardiac Risk Index; stdAnes, standard deviation of MAP during anesthesia.

Across all models, presurgical eGFR and duration of anesthesia ranked among the top predictors based on native FI, with ensemble models and SVM placing eGFR in the top five, and regression models within their top 15 most important features (Figure 1). All other top-ranking features are ranked heterogeneously between ensemble and regression models, where features ranked highly by ensemble models were assigned less importance by regression models, and vice versa. The highest ranking features according to regression models were specific surgery types, i.e., general surgery, neurological surgery, and urological surgery, the ASA score, long-term medication with diuretics, and cirrhosis of the liver. For ensemble models, patient age, presurgical eGFR, duration of anesthesia, and sterofundin dosage during surgery were the four most important predictors (Supplementary Table S5 and Figures S6-13). Feature Importances assigned by the SVM classifier ranked top predictors both from ensemble and regression models slightly lower.

**Fig. 2:**
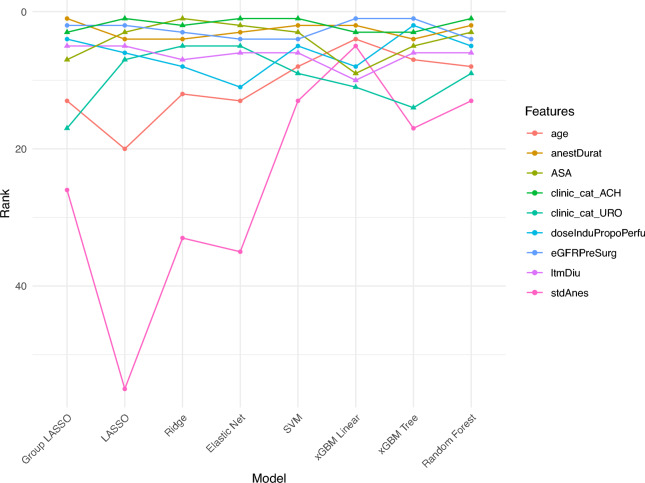
Ranks of Shapley value based Feature Importance (FI) assigned by different PO-AKI prediction models trained on the complete balanced development cohort. Shapley values were averaged across all models and subsequently scaled to the highest average importance before descending ranks of importance were assigned. Lower rank values indicate higher importance with rank 1 representing highest importance. Continuous features are shown as dots, categorical features are displayed as triangles, respectively. Abbrev.: age, patient age; anestDurat, duration of anesthesia; ASA, American Society of Anesthesiology physical status; clinic_cat_ACH, general surgery; clinic_cat_URO, urology surgery; doseInduPropoPerfusor, dose of propofol administered via perfusor during induction; eGFRPreSurg, estimated glomerular filtration rate before surgery; ltmDiu, longterm medication with diuretics; stdAnes, standard deviation of MAP during anesthesia.

For further contextualization, average feature contributions to model predictions were computed in form of Shapley values and ranked accordingly. The five most important features for every model are displayed in Figure 2. All models include anesthesia duration, general surgery and presurgical eGFR in their top five features. ASA is also ranked among the latter by all models except for the Group LASSO regression and linear xGBM models. Additional top predictors included age, propofol dosage, and standard deviation of MAP during induction. Note that the MAP standard deviation and patient age were selected as one of the top five predictors only by the linear xGBM model while ranked very heterogeneously by all other models (Supplementary Table S6 and Supplementary Figures S14-21).

Training of AKI severity stage-specific RFC models was performed on comparatively small balanced development data sets (*n* = 802 patients without AKI and *n* = 802 patients with AKI stage 1, *n* = 181 patients without AKI and *n* = 181 patients with AKI stage 2, *n* = 160 patients without AKI and *n* = 160 patients with AKI stage 3). Models trained on subsets of the development and validation data sets, that only contained specific AKI stage 1 or 2 outcomes achieved slightly worse performances in terms of ROC-AUC, sensitivity, and Balanced Accuracy compared to the model trained on the complete development cohort, while model specificity slightly increased (Supplementary Table S7). In contrast, the RFC prediction model trained on the AKI stage 3 subset yielded higher predictive performances across ROC-AUC, sensitivity, specificity and Balanced Accuracy. With respect to both native FIs and Shapley values, patient age, duration of anesthesia, and presurgical eGFR still remained the top 5 predictors across all AKI stage-specific models (Supplementary Figures S22-S28). Stage 1 prediction models also ranked trend and skewness of MAP time series, urological surgery, and long-term medication with diuretics as their most important predictors, while stage 3 predictors valued propofol dosage during surgery higher compared to the other models.

Due to a high relative importance of presurgical eGFR across most models, an additional sensitivity analysis was performed where the predictor was removed from training and development data set before training an additional RFC model. A comparison of model performances and FI estimations can be seen in Supplementary Table S8 and Supplementary Figure S29. The newly trained RFC model achieved a slightly worse performance than the full model across all metrics. After the removal of preoperative eGFR, the AUC of MAP measurements under 65 mmHg gained much FI in the native estimations, while patient age, ASA and long-term medication with diuretics were assigned higher FI by the post-hoc estimations. Top 30 FIs of the respective model are displayed in Supplementary Figures S30-31.

### Time course development of available predictor variables

Early identification of PO-AKI, ideally already at a pre- or intraoperative time point, is paramount for the initiation of therapeutic treatments. Therefore, we investigated the performance of RFCs trained separately at eight different time points pre-, intra-, and postoperatively. Adjustments that were made to the training and validation data set are described in detail in Supplementary File S2. The performance of these RFCs, trained on the balanced development data set, for predicting PO-AKI on the validation data set varied across the different perioperative anesthesia time points (Figure 4, Supplementary Table S9 and Supplementary Figure S32). The preoperative model achieved a ROC-AUC of 0.722 (95% CI, 0.703 to 0.746), an F1-score of 0.321 (95% CI, 0.299 to 0.346), and a BACC of 0.661 (95% CI, 0.642 to 0.683). At the end of induction, the ROC-AUC, the F1-score and the BACC decreased slightly. The postoperative model showed the best overall performance with a ROC-AUC of 0.738 (95% CI, 0.716 to 0.755), an F1-score of 0.326 (95% CI, 0.301 to 0.351), and a BACC of 0.670 (95% CI, 0.648 to 0.689).

**Fig. 3:**
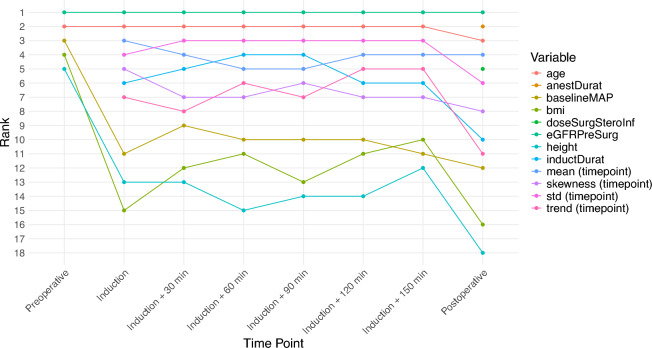
Native Feature Importance (FI) ranks of the five most important features across all perioperative time points of the Random Forest classifier models trained on the balanced development cohort. The plot displays the rank trajectories of those variables that were among the top five features at any time point. Ranks were determined within each model based on native FI values. Lower rank values indicate higher importance with rank 1 representing highest importance. To facilitate comparability across time points, the variable names of features in the MAP time series for different time points were transferred to higher-level categories (labeled by “timepoint”). Abbrev.: age, patient age; anestDurat, duration of anesthesia; ASA, American Society of Anesthesiology physical status; baselineMAP, first measured MAP value; doseInduRemifPerfu, dose of remifentanil administered via perfusor during induction; height, patient height; inductDurat, duration of induction of surgery; kurtosis (timepoint), kurtosis of MAP during corresponding timepoint; mean (timepoint), mean MAP during corresponding timepoint; trend (timepoint), trend of MAP during corresponding timepoint; urgency, level of urgency of surgical intervention.

The native FI analysis revealed that the preoperative eGFR value received the highest FI across all time points, including the preoperative and the postoperative model (Figure 3, Supplementary Table S10). Age was among the three most important features in all perioperative models. Other preoperative variables with high FI ranks were baseline MAP, BMI, height, and the ASA score. At the end of the induction phase, the duration of induction had a high FI rank (rank 6). It remained among the ten most important variables in all subsequent intra- and postoperative phases. The intraoperative characteristics of the MAP time series such as trend, mean, kurtosis, and skewness exhibited high FIs at all intraoperative time points, with FI ranks ranging between 3 and 11. Postoperatively, the total duration of anesthesia (rank 2 at this time point) was found to be of additional feature importance (Figure 3, Supplementary Table S11). In contrast, intraoperative variables relating to anesthesia administration, such as propofol or remifentanil, did not rank among the ten most important variables at any perioperative time point. The RFC trained on the preoperative feature set is also implemented at [https://post-operative-aki.ckdn.app] and [https://github.com/JoLeins/risk-calculator-PO-AKI].

**Fig. 4:**
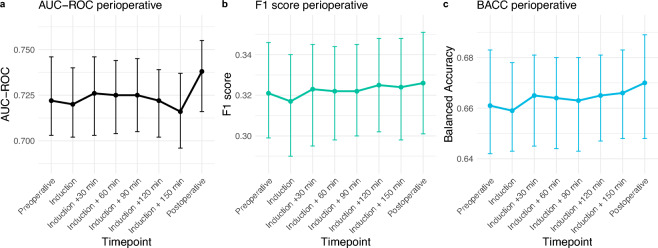
Displayed are the Area under the Receiver Operating Characteristic curve (ROC-AUC) (**a**), F1-score (**b**), and Balanced Accuracy (BACC) (**c**) of the Random Forest classifier models trained on the balanced development dataset and evaluated on the validation cohort. Each metric is reported as mean±95% confidence interval based on 100 bootstrap replicates. Time points cover the preoperative phase, induction of anesthesia, selected intraoperative intervals, and the immediate postoperative phase.

## Discussion

This study presents a comprehensive evaluation of eight different interpretable machine learning algorithms trained for the prediction of post-operative acute kidney injury on a large, single-center, electronic health record database. The Random Forest Classifier achieved the best predictive performance for the occurrence of PO-AKI on an internal validation data set. Overall, models trained on a balanced data set yielded significantly higher performance compared to those trained on an unbalanced data set. For the performance estimation of our models we see clear differences between ROC-AUC and Balanced Accuracy (BACC) metrics compared to, e.g., Precision and F1 Score. This can be explained by the very unbalanced outcome class distribution within our validation data set, to which Precision and F1 Score are more sensitive than ROC-AUC and BACC, as well as the fact that the decision threshold for this estimation was set arbitrarily to 0.5. Notably, the provided predicted probabilities do not represent the real clinical risk, since for the presented analysis no calibration was performed. Training on a balanced development data set combined with the real validation set incidence of 12.42%, resulted in the model systematically overestimating outcome probabilities, with a slightly compressed range of predicted probabilities and poor probabilistic accuracy, as displayed in the calibration curve and associated measures. It is, therefore, recommended that a calibration on a target data set is performed and evaluated, if a direct interpretation of predicted probabilities given by our models is desired. While ROC-AUC analyses judged model performance across all decision thresholds, Precision and F1 Score only evaluated this specific decision threshold of 0.5. With respect to Balanced Accuracy, a performance measure which explicitly accounts for different outcome class distributions in validation data sets, our RFC achieved similar diagnostic performance as the model of Xue et al. trained on perioperative data (BACC: 0.678), and only slightly inferior performance in comparison to the temporal enrichment study of Adhikari et al. (BACC: 0.790)^16,56^.

The evaluation of RFC models, trained across different perioperative time points, revealed only minor changes in the predictive performance for PO-AKI in our study. This suggests that intraoperative data may offer limited incremental value when added to preoperative models. Our finding is consistent with previous studies that have reported only marginal benefits from incorporating intraoperative features^16^. Similarly, Zhou et al. demonstrated that preoperative models alone achieved ROC-AUC values ranging from 0.71 to 0.75 across multiple external validation cohorts, supporting the robustness of preoperative predictors^57^. Sun et al. also reported that restricting potential predictors to preoperative variables or a limited subset of top predictors only slightly affected overall model performance^58^. However, other studies have reported more significant benefits from integrating intraoperative data, particularly when advanced modelling techniques or high-resolution intraoperative monitoring are employed^56^.

The value of complex intraoperative data collection and interpretation appears limited, especially given the significant effort required for data processing and harmonisation. This is supported by a recent critical evaluation of perioperative modelling strategies for PO-AKI, which highlights the marginal added value of intraoperative data compared to preoperative risk stratification^59^. Including detailed intraoperative characteristics may increase the model’s complexity without proportionally increasing its predictive accuracy or clinical applicability^60^.

Our evaluation of native feature importances in different ML algorithms revealed a strong systematic difference in ensemble versus non-ensemble models. Presurgical eGFR and anesthesia duration have yielded the highest average importance across native FI estimations. Average Shapley Additive Explanation values further supported the importance estimation for eGFR, anesthesia duration as well as the surgery type “general surgery”. Estimations across models utilizing Shapley values did not show systematic differences between model types in contrast to native FI estimations, suggesting the latter to be mainly guided by algorithmic individualities in the respective models’ estimations.

The RFC model trained after removing preoperative eGFR as a predictor achieved only slightly lower performance than the best performing classifier. Both native and post-hoc Feature Importance estimations remained relatively unchanged with age gaining relative importance in both measures, and the AUC for MAP measurements under 65 mmHg rising to be a top 5 predictor in native estimation after removal of preoperative eGFR. AKI stage 1 or 2-specific models showed lower predictive performance in comparison to the RFC model trained on the complete development cohort. In contrast, the RFC model trained on study participants not experiencing PO-AKI and only those developing AKI stage 3 achieved improved performance metrics. These results should, however, be interpreted cautiously due to the rather small sample sizes of AKI stage 2 and 3 patients in both our development (*n* = 181 and *n* = 160) and validation data sets (*n* = 100 and *n* = 69). Native Feature Importance and Shapley value analyses for all three models reinforced the results of the full models. While the stage 1 model valued intraoperative MAP trend and skewness higher compared to the stage 2 and 3 models, that conversely included intraoperative propofol dosages in their top 5 predictors, most other top 5 predictors were rated similarly across stage-specific models. To further validate these findings, assessments within larger cohorts that include more patients particularly suffering from AKI stage 2 or 3 are required. Importantly, the estimations of feature importance provide only correlative indication to potential causal relationships. All associations identified by our predictive models Feature Importance assignments need to be further validated in order to verify causal dependency.

Overall, patient characteristics collected prior to anesthesia as well as during the initial phase of anesthesia induction appeared to be the most important risk factors for PO-AKI according to the different ML models. Key renal-specific indicators included preoperative eGFR, long-term medication with diuretics, and urological procedures, which often imply a pre-existing nephrological burden. In addition, extensive surgical procedures, such as major abdominal surgery, are inherently associated with a higher physiological burden^61^. The induction phase of general anesthesia can unintentionally test cardiovascular reserve^62^. Additionally, the duration of induction may serve as a surrogate marker for a technically or physiologically complex induction. During this phase, particularly hypotension following induction has been linked to an increased risk of subsequent organ dysfunction in previous studies^63^.

Although our findings showed the established association between intraoperative hypotension and PO-AKI, as, e.g., suggested by Wesselink et al., the incremental predictive value of routinely documented intraoperative MAP features appeared lower than expected^6^. The distinction between causal relevance and incremental discrimination is of significance, as a clinically relevant risk factor may contribute only limited additional predictive information beyond dominant baseline variables. The observed effect may partly reflect routine anesthetic practice where MAP *\ge* 65 mmHg is actively maintained, leading to early correction and underrepresentation of hypotensive events, which limits their detectability and prognostic value in retrospective models^64^. Recent Randomized Controlled Trials further suggest that individualized blood pressure control does not prevent perioperative complications^65,66^. Furthermore, our analysis focused primarily on blood-pressure-derived features and did not assess the full spectrum of potentially relevant intraoperative predictors. In contrast, Park et al. reported higher predictive performance using minute-level intraoperative monitoring data, suggesting that higher-resolution physiological data may provide greater incremental value^67^.

Our study has several limitations. During the creation of the present data set, large proportions of the complete Electronic Health Record (EHR) database had to be excluded due to incomplete documentation, implausible values, or missing follow-up data. This considerable attrition rate, in addition to the necessity for regular postoperative creatinine measurements and a minimum postoperative hospital stay of seven days, may have led to a potential selection bias towards patients with a more complex clinical course. Although the selected cohort was slightly older and underwent longer anesthesia induction and surgical procedures, the overall baseline characteristics did not indicate a substantially higher-risk population, suggesting that the observed differences likely reflect a combination of perioperative management, data completeness, and the application of analysis-specific exclusion and data quality criteria rather than patient risk alone. The intraoperative MAP time series data were preprocessed using locally limited outlier filtering, but alternative methods could lead to deviating results. In addition, urine output was not included in the PO-AKI definition because reliable and standardized urine output measurements were not consistently available in the retrospective EHR dataset. Consequently, the non-inclusion of urine output might have limited the precision of our outcome definitions and restrict the model to PO-AKI classification based on serum creatinine^68,69^. The exclusion of patients with chronic renal insufficiency was based on routine clinical documentation in the electronic anesthesia information system rather than a systematic KDIGO-based classification. Consequently, patients with varying degrees of baseline renal impairment may still have been represented within the study cohort, which may partly explain the consistently high predictive importance of preoperative eGFR observed across multiple models. Moreover, the analysis did not incorporate mortality as an outcome due to the unavailability of reliable follow-up data beyond the initial hospitalization, thereby restricting the validity of potential mortality analyses. The clinical value of the provided risk score was also found to yield only slight benefits in clinical application and would need to be reevaluated in external validation settings. Furthermore, our PO-AKI risk scores were developed and validated retrospectively. Prospective external validation in independent, real-world cohorts is therefore the necessary next step before clinical implementation can be considered. Beyond confirming predictive performance, subsequent impact studies would be required to determine whether score-guided risk stratification translates into changes in management and improved patient-relevant outcomes.

Given a heterogeneous estimation of feature impact by ensemble and non-ensemble models, the interpretability of many FIs remains limited, even if considering additional post-hoc FI estimations. Another limiting factor to their interpretational impact is given by the fact that neither suitable datasets for external validation of our newly developed ML models nor data on predictor variables of already published PO-AKI risk scores were available for benchmarking in our database. All performance comparisons with other models were therefore only performed with the respective internal validation results, clearly limiting the generalizability of the latter. Particularly the applicability of our risk score in clinical practice is impacted by this constraint. The provided models, therefore, serve as a proof-of-concept but require external validation, ideally on one or several multi-center data sets to avoid overestimation of model performance and to demonstrate generalizability of our models. With the provision of our pre- and postoperative RFC models as both an easy-to-use webservice and a freely available GitHub resource, we aim to ease the explorative implementation of our risk scores for clinicians and also improve possibilities for comparative benchmarking in the field. Our implemented risk calculators depend on the availability of complete, high quality input data to achieve the postulated model precisions. For the application, users are required to process MAP time series employing the R script as provided in the GitHub repository.

In conclusion, our work provides a comprehensive, reproducible, and transparent approach to PO-AKI risk factor analysis, utilizing ML model-specific measures as well as model-agnostic post-hoc tests to assess the impact of features on model predictions. Leveraging a large, heterogeneous EHR database, we were able to match the predictive performance of similar ML models on a real-world dataset, while offering comprehensible insights into feature impact for our models’ predictions. Adding perioperative data, such as MAP measurements, led to a visible, but not statistically significant increase in predictive performance, suggesting a limited incremental predictive value of routinely documented MAP-derived features beyond strong baseline risk factors in our cohort. We furthermore observed systematic differences in native FI estimations between ensemble and non-ensemble ML algorithms, encouraging future research in this area. By making our random forest models available as an online risk calculator and open source code, we aim to facilitate benchmarking and foster transparency. Our findings highlight both the potential and current limitations of interpretable machine learning models for PO-AKI prediction and underscore the need for further validation and refinement, particularly regarding the integration of higher-resolution perioperative data to enhance their clinical utility. With this work, we answered a call for more transparent implementations of interpretable machine learning solutions in the field of PO-AKI prediction^5^.

## Supplementary Information

Supplementary Information.

## Data Availability

The data underlying this study cannot be shared publicly due to European and federal data protection regulations. De-identified individual participant data that underlie the results reported in this article, together with the R analysis protocol, will be made available from the corresponding author upon reasonable request, beginning three months and up to ten years following article publication. Access to patient data is subject to approval by the data protection officer of Hannover Medical School. Requests can be directed to Hans-Joerg Gillmann (gillmann.hans-joerg@mh-hannover.de) or Thomas Stüber (stueber.thomas@mh-hannover.de), with formal approval coordinated via the data protection officer (datenschutz@mh-hannover.de).

## References

[1] Boyer, N., Eldridge, J., Prowle, J. R. & Forni, L. G. Postoperative acute kidney injury. *Clinical Journal of the American Society of Nephrology* **17**, 1535–1545 (2022). 35710717 10.2215/CJN.16541221 PMC9528271

[2] Zarbock, A. et al. Epidemiology of surgery associated acute kidney injury (epis-aki): a prospective international observational multi-center clinical study. *Intensive Care Medicine* **49**, 1441–1455 (2023). 37505258 10.1007/s00134-023-07169-7 PMC10709241

[3] Prowle, J. R. et al. Postoperative acute kidney injury in adult non-cardiac surgery: joint consensus report of the acute disease quality initiative and perioperative quality initiative. *Nature Reviews Nephrology* **17**, 605–618 (2021). 33976395 10.1038/s41581-021-00418-2 PMC8367817

[4] Gameiro, J. et al. Acute kidney injury, long-term renal function and mortality in patients undergoing major abdominal surgery: a cohort analysis. *Clinical Kidney Journal* **9**, 192–200 (2016). 26985368 10.1093/ckj/sfv144 PMC4792619

[5] Takkavatakarn, K. & Hofer, I. S. Artificial intelligence and machine learning in perioperative acute kidney injury. *Advances in Kidney Disease and Health* **30**, 53–60 (2023). 36723283 10.1053/j.akdh.2022.10.001 PMC12228575

[6] Wesselink, E., Kappen, T., Torn, H., Slooter, A. & van Klei, W. Intraoperative hypotension and the risk of postoperative adverse outcomes: a systematic review. *British Journal of Anaesthesia* **121**, 706–721 (2018). 30236233 10.1016/j.bja.2018.04.036

[7] Liu, J. et al. Incidence and risk factors of acute kidney injury after abdominal surgery: a systematic review and meta-analysis. *Ann. Med.*10.1080/07853890.2025.2547324 (2025). 40819346 10.1080/07853890.2025.2547324 PMC12360056

[8] Group et al. Kdigo clinical practice guideline for acute kidney injury. *Kidney Int. Suppl.* **2**, 1 (2012).

[9] Zacharias, H. U. et al. A predictive model for progression of ckd to kidney failure based on routine laboratory tests. *American Journal of Kidney Diseases* **79**, 217-230.e1 (2022). 34298143 10.1053/j.ajkd.2021.05.018

[10] Zacharias, H. U. et al. Analysis of human urine reveals metabolic changes related to the development of acute kidney injury following cardiac surgery. *Metabolomics* **9**, 697–707 (2013).

[11] Zacharias, H. U. et al. Identification of plasma metabolites prognostic of acute kidney injury after cardiac surgery with cardiopulmonary bypass. *Journal of Proteome Research* **14**, 2897–2905 (2015). 26036910 10.1021/acs.jproteome.5b00219

[12] Thottakkara, P. et al. Application of machine learning techniques to high-dimensional clinical data to forecast postoperative complications. *PLOS ONE* **11**, e0155705 (2016). 27232332 10.1371/journal.pone.0155705 PMC4883761

[13] Vagliano, I. et al. Machine learning models for predicting acute kidney injury: a systematic review and critical appraisal. *Clinical Kidney Journal* **15**, 2266–2280 (2022). 36381375 10.1093/ckj/sfac181 PMC9664575

[14] Lee, Y. et al. Machine learning-based prediction of acute kidney injury after nephrectomy in patients with renal cell carcinoma. *Scientific Reports* **11**, 15704 (2021). 34344909 10.1038/s41598-021-95019-1 PMC8333365

[15] Mathis, M. R. et al. Preoperative risk and the association between hypotension and postoperative acute kidney injury. *Anesthesiology* **132**, 461–475. 10.1097/ALN.0000000000003063 (2020). 31794513 10.1097/ALN.0000000000003063 PMC7015776

[16] Xue, B. et al. Use of machine learning to develop and evaluate models using preoperative and intraoperative data to identify risks of postoperative complications. *JAMA network open* **4**, e212240–e212240 (2021). 33783520 10.1001/jamanetworkopen.2021.2240 PMC8010590

[17] R Core Team. R: A Language and Environment for Statistical Computing (R Foundation for Statistical Computing, Vienna, Austria, 2024).

[18] Van Rossum, G. & Drake, F. L. *Python 3 Reference Manual* (CreateSpace, Scotts Valley, CA, 2009).

[19] Schauberger, P. & Walker, A. openxlsx: Read, write and edit xlsx files (2024). R package version 4.2.5.2.

[20] Wickham, H., François, R., Henry, L., Müller, K. & Vaughan, D. dplyr: A grammar of data manipulation (2023). R package version 1.1.4.

[21] Wickham, H., Vaughan, D. & Girlich, M. tidyr: Tidy messy data (2024). R package version 1.3.1.

[22] Wickham, H. *ggplot2: Elegant Graphics for Data Analysis* (Springer-Verlag, New York, 2016).

[23] Zeileis, A. & Grothendieck, G. zoo: S3 infrastructure for regular and irregular time series. *Journal of Statistical Software* **14**, 1–27 (2005).

[24] Wickham, H. Reshaping data with the reshape package. *Journal of Statistical Software* **21**, 1–20 (2007).

[25] Komsta, L. & Novomestky, F. moments: Moments, cumulants, skewness, kurtosis and related tests (2022). R package version 0.14.1.

[26] Pohlert, T. trend: Non-parametric trend tests and change-point detection (2023) (R package version 1.1.6).

[27] pandas development team. *T. pandas-dev/pandas: Pandas*10.5281/10.5281/zenodo.10957263 (2024).

[28] Harris, C. R. et al. Array programming with NumPy. *Nature* **585**, 357–362 (2020). 32939066 10.1038/s41586-020-2649-2 PMC7759461

[29] Yoshida, K. & Bartel, A. *tableone: Create ’Table 1’ to Describe Baseline Characteristics with or without Propensity Score Weights* (2022). R package version 0.13.2.

[30] Seabold, S. & Perktold, J. statsmodels: Econometric and statistical modeling with python. In: *Proceedings of the 9th Python in Science Conference* (2010).

[31] Harrell Jr, F. E. Hmisc: Harrell miscellaneous (2024). R package version 5.1-3.

[32] Revelle, William. psych: Procedures for psychological, psychometric, and personality research (2024) (R package version 2.4.6).

[33] R Core Team,. R: A language and environment for statistical computing (2023).

[34] Kassambara, A. ggcorrplot: Visualization of a correlation matrix using ’ggplot2’ (2023). R package version 0.1.4.1.

[35] Ranstam, J. & Cook, J. A. Lasso regression. *British Journal of Surgery* **105**, 1348–1348 (2018).

[36] Tibshirani, R. Regression shrinkage and selection via the lasso. *Journal of the Royal Statistical Society Series B: Statistical Methodology* **58**, 267–288 (1996).

[37] Meier, L., Van De Geer, S. & Bühlmann, P. The group lasso for logistic regression. *Journal of the Royal Statistical Society Series B: Statistical Methodology* **70**, 53–71 (2008).

[38] Hoerl, A. E. & Kennard, R. W. Ridge regression: Biased estimation for nonorthogonal problems. *Technometrics* **12**, 55–67 (1970).

[39] Cortes, C. & Vapnik, V. Support-vector networks. *Machine Learning* **20**, 273–297 (1995).

[40] Breiman, L. Random forests. *Machine Learning* **45**, 5–32 (2001).

[41] Friedman, J. H. Greedy function approximation: a gradient boosting machine. *Annals of Statistics* 1189–1232 (2001).

[42] Chen, T. Xgboost: A scalable tree boosting system (Cornell University, 2016).

[43] Zou, H. & Hastie, T. Regularization and variable selection via the elastic net. *Journal of the Royal Statistical Society Series B: Statistical Methodology* **67**, 301–320 (2005).

[44] Friedman, J., Tibshirani, R. & Hastie, T. Regularization paths for generalized linear models via coordinate descent. *Journal of Statistical Software* **33**, 1–22 (2010). 20808728 PMC2929880

[45] Yang, Y. & Zou, H. A fast unified algorithm for solving group-lasso penalize learning problems. *Statistics and Computing* **25**, 1129–1141. 10.1007/s11222-014-9498-5 (2015).

[46] Wright, M. N. & Ziegler, A. ranger: A fast implementation of random forests for high dimensional data in C++ and R. *Journal of Statistical Software* **77**, 1–17 (2017).

[47] Kuhn, M. Building predictive models in r using the caret package. *Journal of Statistical Software* **28**, 1–26 (2008).27774042

[48] Hofmann, T., Schölkopf, B. & Smola, A. J. Kernel methods in machine learning. *The Annals of Statistics* **36** (2008).

[49] Vert, J.-P., Tsuda, K. & Schölkopf, B. A Primer on Kernel Methods. In *Kernel Methods in Computational Biology*, 35–70 (The MIT Press, 2004).

[50] Karatzoglou, A., Smola, A., Hornik, K. & Zeileis, A. kernlab - an S4 package for kernel methods in R. *Journal of Statistical Software* **11**, 1–20 (2004).

[51] Chen, T., & Guestrin, C. XGBoost: A Scalable Tree Boosting System. Proceedings of the 22nd ACM SIGKDD International Conference on Knowledge Discovery and Data Mining (2016).

[52] Kuhn, M. & Wickham, H. Tidymodels: a collection of packages for modeling and machine learning using tidyverse principles. (2020).

[53] Vickers, A. J. & Elkin, E. B. Decision curve analysis: A novel method for evaluating prediction models. *Medical Decision Making* **26**, 565–574. 10.1177/0272989X06295361 (2006). 17099194 10.1177/0272989X06295361 PMC2577036

[54] Harrell Jr, F. E. *rms: Regression Modeling Strategies* (2026). R package version 8.1-1.

[55] Mayer, M. & Watson, D. kernelshap: Kernel shap (2024). R package version 0.7.0.

[56] Adhikari, L. et al. Improved predictive models for acute kidney injury with idea: Intraoperative data embedded analytics. *PLOS ONE* **14**, e0214904 (2019). 30947282 10.1371/journal.pone.0214904 PMC6448850

[57] Zhuo, X. Y. Preoperative risk prediction models for acute kidney injury after noncardiac surgery: an independent external validation cohort study. Br. J. Anaesthesia (2024). 10.1016/j.bja.2024.02.018 38527923

[58] Sun, R. Development of interpretable machine learning models for prediction of acute kidney injury after noncardiac surgery: a retrospective cohort study. Int. J. Surgery (2024). 10.1097/JS9.0000000000001237 PMC11093510 38445452

[59] McIlroy, D. R. Predictive modelling for postoperative acute kidney injury: big data enhancing quality or the emperor’s new clothes?. *British Journal of Anaesthesia* **133**, 476–478 (2024). 38902116 10.1016/j.bja.2024.05.013

[60] Monk, T. G. et al. Association between intraoperative hypotension and hypertension and 30-day postoperative mortality in noncardiac surgery. *Anesthesiology* **123**, 307–319 (2015). 26083768 10.1097/ALN.0000000000000756

[61] Kim, M., Brady, J. E. & Li, G. Variations in the risk of acute kidney injury across intraabdominal surgery procedures. *Anesthesia & Analgesia* **119**, 1121–1132 (2014). 25191972 10.1213/ANE.0000000000000425

[62] Südfeld, S. et al. Post-induction hypotension and early intraoperative hypotension associated with general anaesthesia. *British Journal of Anaesthesia* **119**, 57–64 (2017). 28974066 10.1093/bja/aex127

[63] Maheshwari, K. et al. The association of hypotension during non-cardiac surgery, before and after skin incision, with postoperative acute kidney injury: a retrospective cohort analysis. *Anaesthesia* **73**, 1223–1228 (2018). 30144029 10.1111/anae.14416

[64] Sessler, D. I. et al. Perioperative quality initiative consensus statement on intraoperative blood pressure, risk and outcomes for elective surgery. *British Journal of Anaesthesia* **122**, 563–574 (2019). 30916004 10.1016/j.bja.2019.01.013

[65] Saugel, B. et al. Individualized perioperative blood pressure management in patients undergoing major abdominal surgery. *JAMA* **334**, 1893 (2025). 41076588 10.1001/jama.2025.17235 PMC12516512

[66] Kant, M. et al. Proactive vs reactive treatment of hypotension during surgery. *JAMA* **334**, 1905 (2025). 41076587 10.1001/jama.2025.18007 PMC12516513

[67] Park, S. et al. A deep-learning algorithm using real-time collected intraoperative vital sign signals for predicting acute kidney injury after major non-cardiac surgeries: A modelling study. *PLOS Medicine* **22**, e1004566 (2025). 40299885 10.1371/journal.pmed.1004566 PMC12040160

[68] Malbrain, M. L. N. G. et al. Urine output is an early and strong predictor of acute kidney injury and associated mortality: A systematic literature review of 50 clinical studies. *Annals of Intensive Care* **14**, 110. 10.1186/s13613-024-01342-x (2024). 38980557 10.1186/s13613-024-01342-x PMC11233478

[69] Bufkin, K. B., Karim, Z. A. & Silva, J. Review of the limitations of current biomarkers in acute kidney injury clinical practices. *SAGE Open Medicine* **12** (2024). 10.1177/20503121241228446 PMC10846001 38322582

