# Supplementary material for: Identification of acute kidney injury after non-cardiac surgery by machine learning leveraging a large electronic health record database
Source: Sci Rep. 2026 Aug 18;16:25909. doi: 10.1038/s41598-026-65849-y (PMC13486790; doi:10.1038/s41598-026-65849-y)
Supplement: Supplementary file 1 [file 41598_2026_65849_MOESM1_ESM.pdf]

Supplementary Material for: Identification of Acute Kidney Injury after Non-Cardiac Surgery by Machine Learning Leveraging a Large Electronic Health Record Database

Jonas Leins[1,+], Hendrik Meyer[2,+], Laurenz Engel[1], Christian Hinze[3], Hans-Jörg Gillmann[2,++], Thomas Stüber[2,++], and Helena U. Zacharias[1,++,*]

[1]Hannover Medical School, Peter L. Reichertz Institute for Medical Informatics of TU Braunschweig and Hannover Medical School, Hannover, 30625, Germany
[2]Hannover Medical School, Department of Anesthesiology and Intensive Care Medicine, Hannover, 30625, Germany
[3]Hannover Medical School, Department of Nephrology and Hypertension, Hannover, 30625, Germany
[+]these authors contributed equally to this work
[++]shared senior authors
[*]zacharias.helena@mh-hannover.de

Contents

1 Supplementary Files 5
1.1 Supplementary File S1: Study population selection . . . 5
1.2 Supplementary File S2: Data collection and preparation . . . 5
1.3 Supplementary File S3: Extraction of Mean Arterial Pressure Time Series Features . . . 6
1.4 Supplementary File S4: In- and Exclusion of Correlated Predictor Variables . . . 7
1.5 Supplementary File S5: Feature Importance Assessment . . . 8
1.6 Supplementary File S6: Influence of Balanced and Unbalanced Development Cohorts on Model Performance . . . 8
2 Supplementary Figures 9
3 Supplementary Tables 40
References 57

List of Figures

1 Study population selection . . . 9
2 Multicollinearity assessment of predictor variable candidates. . . . 10
3 Receiver Operating Characteristics and Precision-Recall curves of machine learning models trained on the balanced development data set and evaluated on the validation data set. . . . 11
4 Decision Curve Analysis of best performing RFC model. . . . 12
5 Calibration curve of best performing RFC model on the full validation data set. . . . 12
6 Scaled native Feature Importances (FIs) of the 30 most important predictors, assigned by a logistic Group-LASSO regression model trained on the complete balanced development cohort for the prediction of post-operative acute kidney injury after non-cardiac surgery. . . . 13
7 Scaled native Feature Importances (FIs) of the 30 most important predictors, assigned by a logistic LASSO regression model trained on the complete balanced development cohort for the prediction of post-operative acute kidney injury after non-cardiac surgery. . . . 14
8 Scaled native Feature Importances (FIs) of the 30 most important predictors, assigned by a logistic Ridge regression model trained on the complete balanced development cohort for the prediction of post-operative acute kidney injury after non-cardiac surgery. . . . 15
9 Scaled native Feature Importances (FIs) of the 30 most important predictors, assigned by a logistic Elastic Net regression model trained on the complete balanced development cohort for the prediction of post-operative acute kidney injury after non-cardiac surgery. . . . 16
10 Scaled native Feature Importances (FIs) of the 30 most important predictors, assigned by a Support Vector Machine using a radial kernel function, trained on the complete balanced development cohort for the prediction of post-operative acute kidney injury after non-cardiac surgery. . . . 17
11 Scaled native Feature Importances (FIs) of the 30 most important predictors, assigned by an extreme Gradient Boosting Machine with a linear regression algorithm as base learner, trained on the complete balanced development cohort for the prediction of post-operative acute kidney injury after non-cardiac surgery. . . . 18
12 Scaled native Feature Importances (FIs) of the 30 most important predictors, assigned by an extreme Gradient Boosting Machine with a Decision Tree algorithm as base learner, trained on the complete balanced development cohort for the prediction of post-operative acute kidney injury after non-cardiac surgery. . . . 19
13 Scaled native Feature Importances (FIs) of the 30 most important predictors, assigned by a Random Forest model trained on the complete balanced development cohort for the prediction of post-operative acute kidney injury after non-cardiac surgery. . . . 20
14 Scaled average Shapley values of the 30 most important predictors, assigned by a logistic Group-LASSO regression model trained on the complete balanced development cohort for the prediction of post-operative acute kidney injury after non-cardiac surgery. . . . 21
15 Scaled average Shapley values of the 30 most important predictors, assigned by a logistic LASSO regression model trained on the complete balanced development cohort for the prediction of post-operative acute kidney injury after non-cardiac surgery. . . . 22
16 Scaled average Shapley values of the 30 most important predictors, assigned by a logistic Ridge regression model trained on the complete balanced development cohort for the prediction of post-operative acute kidney injury after non-cardiac surgery. . . . 23
17 Scaled average Shapley values of the 30 most important predictors, assigned by a logistic Elastic Net regression model trained on the complete balanced development cohort for the prediction of post-operative acute kidney injury after non-cardiac surgery. . . . 24
18 Scaled average Shapley values of the 30 most important predictors, assigned by a SVM model trained on the complete balanced development cohort for the prediction of post-operative acute kidney injury after non-cardiac surgery. . . . 25
19 Scaled average Shapley values of the 30 most important predictors, assigned by a linear xGBM model trained on the complete balanced development cohort for the prediction of post-operative acute kidney injury after non-cardiac surgery. . . . 26
20 Scaled average Shapley values of the 30 most important predictors, assigned by a tree-based xGBM model trained on the complete balanced development cohort for the prediction of post-operative acute kidney injury after non-cardiac surgery. . . . 27
21 Scaled average Shapley values of the 30 most important predictors, assigned by a Random Forest model trained on the complete balanced development cohort for the prediction of post-operative acute kidney injury after non-cardiac surgery. . . . 28

22 Ranks of native and post-hoc assessed Feature Importance (FI) assigned by PO-AKI prediction models trained with different PO-AKI stages as outcome variable. . . . 29
23 Scaled native Feature Importances (FIs) of the 30 most important predictors, assigned by a Random Forest Classifier (RFC), trained on a balanced development cohort for the prediction of post-operative acute kidney injury stage 1 after non-cardiac surgery. . . . 30
24 Scaled native Feature Importances (FIs) of the 30 most important predictors, assigned by a Random Forest Classifier (RFC), trained on a balanced development cohort for the prediction of post-operative acute kidney injury stage 2 after non-cardiac surgery. . . . 31
25 Scaled native Feature Importances (FIs) of the 30 most important predictors, assigned by a Random Forest Classifier (RFC), trained on a balanced development cohort for the prediction of post-operative acute kidney injury stage 3 after non-cardiac surgery. . . . 32
26 Scaled average Shapley values of the 30 most important predictors, assigned by a Random Forest Classifier (RFC), trained on a balanced development cohort for the prediction of post-operative acute kidney injury stage 1 after non-cardiac surgery. . . . 33
27 Scaled average Shapley values of the 30 most important predictors, assigned by a Random Forest Classifier (RFC), trained on a balanced development cohort for the prediction of post-operative acute kidney injury stage 2 after non-cardiac surgery. . . . 34
28 Scaled average Shapley values of the 30 most important predictors, assigned by a Random Forest Classifier (RFC), trained on a balanced development cohort for the prediction of post-operative acute kidney injury stage 3 after non-cardiac surgery. . . . 35
29 Ranks of native and post-hoc assessed Feature Importance (FI) assigned by PO-AKI prediction models trained on the balanced development cohort without preoperative eGFR measurements. . . . 36
30 Scaled native Feature Importances (FIs) of the 30 most important predictors, assigned by a Random Forest Classifier (RFC) trained on the balanced development cohort without preoperative eGFR measurements. . . . 37
31 Scaled average Shapley values of the 30 most important predictors, assigned by a Random Forest Classifier (RFC) trained on the balanced development cohort without preoperative eGFR measurements. . . . 38
32 Receiver Operating Characteristics and Precision-Recall curves of Random Forest classifiers (RFCs), trained on the balanced development data set at different times points of anesthesia, and evaluated on the validation data set.39

List of Tables

1 Comparison of the complete study cohort and the final study cohort. 40
2 Baseline characteristics of the balanced development cohort. 42
3 Baseline characteristics of validation cohort. 45
4 Model performances of eight different machine learning algorithms in the resampling approach. 48
5 Native Feature Importances (FI) of final classification models trained on the balanced development cohort. 49
6 Average Shapley values of model predictions on the validation data set by final PO-AKI prediction models. . . . 51
7 Performance of different AKI severity stage-specific RFC models 53
8 Performance of RFC models trained on the balanced development cohort with and without preoperative eGFR . . 53
9 Final model performances of Random Forest classifier models, trained on the balanced development cohort at different time points during anesthesia. 53
10 Native Feature Importances (FI) of Random Forest classifiers (RFCs) trained on the balanced development cohort at different perioperative time points. 54
11 Estimation of performance variance dependent on available predictor variable sets across different perioperative time points. 56

1 Supplementary Files

1.1 Supplementary File S1: Study population selection

The study population selection process is illustrated in Supplementary Figure S1. As part of the data selection process, a total of 45,365 patients were excluded from further analysis due to defined exclusion criteria. This included 7,712 cases with cardiac interventions. These were primarily interventions with a heart-lung machine (*n* = 7,444), followed by extracorporeal membrane oxygenation (ECMO, *n* = 183), artificial hearts (*n* = 84), and the use of an intra-aortic balloon pump (*n* = 1). A large proportion of the exclusions (*n* = 39,675) were for procedures that were not performed under general anesthesia with endotracheal intubation. Within this group, patients with alternative intubation or anesthesia techniques were excluded, including laryngeal mask intubation (*n* = 20,779), cases with multiple or no intubation accesses (*n* = 17,462), and procedures performed under spinal (*n* = 74) or peridural (*n* = 1,360) anesthesia. In addition, 280 patients were excluded because of preoperative haemodynamic instability. This was defined by the administration of catecholamines before the start of induction and was used to ensure homogeneity in terms of circulatory status at the time of induction.
A further reason for exclusion was the absence of documented consent for the utilisation of data for research purposes as stipulated in the admission contract. In 21,821 cases, no informed consent could be established, resulting in the exclusion of these patients from the analysis in accordance with ethical and data protection regulations. Additionally, an exclusion based on quality criteria was conducted, leading to the removal of a further 38,079 patients from the analysis. In 2,624 cases, non-standardised or deviating dosage information for perfusor substances was available, which made a comparable interpretation of the medication difficult. In addition, 23 cases were excluded in which atypical opioids were used, which are not usually used during anesthesia. A major reason for exclusion concerned the lack of relevant laboratory parameters: In 32,615 cases, no postoperative creatinine value was available over a period of seven days, which precluded an assessment of the study endpoint. Furthermore, patients who were already on dialysis (*n* = 1,222) and with known chronic renal insufficiency (*n* = 1,595) were excluded, as in these cases the postoperative changes in renal function could not be assessed independently of the pre-existing organ status.
As part of the data cleansing process, a further 11,709 cases were excluded. These exclusions were based on the existence of implausible, incomplete and strongly deviating characteristics as well as extremely rare documented cases. In 187 cases, demographic information outside physiologically plausible value ranges was available. These included extreme heights (<100 cm or >251 cm, *n* = 138), a body weight over 205 kg (*n* = 14) and inconsistent body mass index (BMI) values: either due to strong deviations (>2.5) from the calculated value based on height and weight (*n* = 30) or due to absolute extreme values (<10 or >100, *n* = 5). In addition, relevant values were not documented in 1,406 cases, in particular information on height (*n* = 1,400) and in a few cases also on the DECREASE variable (*n* = 6). Eleven datasets had technically implausible negative values for medication data entries, including for induction with propofol (*n* = 1), remifentanil (*n* = 8) and gelafundin infusions (*n* = 2). 28 cases were excluded due to very infrequent use of certain anesthetic drugs during induction, including fentanyl bolus (*n* = 2), fentanyl perfusor (*n* = 1), hydroxyethyl starch infusions (*n* = 10), midazolam perfusor (*n* = 3), and thiopental perfusor (*n* = 12). Furthermore, 157 cases with extreme medication outliers were identified and excluded. These included etomidate boluses over 1.2 mg/kg bw i.v. (*n* = 4), remifentanil perfusors with more than 4 µg/kg bw/min i.v. (*n* = 1), sterofundin infusions with volumes over 200 ml/kg bw/h during induction of anesthesia (*n* = 29) or over 100 ml/kg bw/h intraoperatively (*n* = 120), as well as severely excessive administration of sufentanil (bolus >0.5 µg/kg bw i.v (*n* = 1), perfusor >6 µg/kg bw/h i.v (*n* = 1)) and thiopental bolus (>20 mg/kg bw i.v (*n* = 1)). As part of the adjustment of the mean arterial pressure time series (MAP) values, 199 cases were excluded. Reasons for exclusion included incomplete documentation (*n* = 6), negative time points for the onset of anesthesia (*n* = 2), unusually long anesthesia durations over 720 minutes (*n* = 172), very short induction phases of less than one minute (*n* = 17), and operation durations of less than one minute (*n* = 2).
Finally, 9,721 cases were excluded due to multiple operations on the same patient within the survey period in order to avoid bias due to non-independent observations and to ensure a clear allocation of effects.

1.2 Supplementary File S2: Data collection and preparation

The potential predictor variables were grouped into eight different categories, including demography, preoperative risk assessment, pre-existing conditions, surgical department, durations of the anesthesia process, long-term medication, anesthesia medication, and MAP features. Demographic variables incorporated, e.g., gender and age. Preoperative risk assessment variables included the last preoperative estimated glomerular filtration rate (eGFR) value and established risk scores, e.g., patient risk classifications according to the American Society of Anesthesiologists (ASA) score.[1] Data with regard to the pre-, intra-, and postoperative anesthesia care was electronically collected and documented with the ANDOK Anesthesia Information Management System (ANDOKlive, DATAPEC GmbH, Pliezhausen, Germany). The data stored in ANDOK corresponds to the medical documentation requirements and is mainly derived from the preoperative anesthesia anamnesis. Medical history is primarily entered into ANDOK via checkboxes, but ANDOK also provides free text fields to complete missing options in the checkboxes. For this study, we used ANDOK data from different subcategories, including demographics,

pre-existing conditions, long-term medication, and preoperative risk assessment. The patient's vital signs and respiratory data were automatically recorded via an interface. Mean arterial blood pressure (MAP) measurements were conducted both invasively and/or non-invasively. In the case of multiple recordings, the values marked as arterial by the anesthetist were given the highest priority, followed by the unmarked invasive measurements and the non-invasive blood pressure cuff measurements, respectively. The MAP time series was recorded at different time intervals for the different types of measurements, i.e., every minute for invasive measurements and every five, three, or one minute for non-invasive measurements, respectively. To obtain a complete MAP time series with values documented every minute, missing values were added according to the last observation carried forward principle.[2]

The bolus, infusion, and syringe pump categories available in ANDOK were used to document anesthetic medication. The administration time-points were categorized into the induction of anesthesia and surgery phase to allow statistical analyses stratified accordingly. Serum creatinine laboratory data was measured using photometry (enzymatic) on the Cobas 8000, module c701 (Roche Diagnostics, Switzerland). Slightly different measurement methods were used during the study period and standardized accordingly, as laboratory measurements had evolved. Perioperative creatinine values were electronically stored in the Hospital Information System (i.s.h.med., Oracle Cerner, Munich), and extracted from the Enterprise Clinical Research Data Warehouse (ECRDW, MHH). The primary endpoint of the study was postoperative acute kidney injury (PO-AKI) as defined by an increase in serum creatinine of $\geq 0{\cdot}3$mg/dl or $\geq 50\%$ of the last pre-operative value within seven days post-operatively according to the KDIGO Clinical Practice Guideline for Acute Kidney Injury 2012.[3] After data merging, inclusion and exclusion criteria were validated, and data was subsequently anonymized.

Following the calculation of MAP time-series features (as referred to in Supplementary File S3) additional samples had to be removed, only for the time-point specific ML analyses. For the intraoperative variables, cases with missing values occurred during the calculation of the MAP features for shortened time windows, which consequently had to be excluded. A total of 87 patients were thus removed from the balanced development dataset and 147 patients from the validation dataset, resulting for each intraoperative timepoint in a total number of n = 2,199 patients in the balanced development dataset and a total number of n = 4,483 patients in the validation dataset, respectively. Accordingly the adjusted PO-AKI incidences for the time point analyses are 49.84% (1,096/2,199) for the development and 12.41% (559/4,483) for the validation data sets, respectively.

1.3 Supplementary File S3: Extraction of Mean Arterial Pressure Time Series Features

In contrast to more complex and less interpretable models like deep neural networks, none of the machine learning algorithms used in this study is capable of incorporating an extra dimension of certain features (e.g., time) within their training process. In order to utilize information contained in the time series MAP data within our dataset, a statistical extraction of single valued features that hold a part of that information is therefore necessary. Feature extraction from MAP time series data has been investigated in several studies, and no superior feature extraction procedure has yet been identified.[4,5] We extracted the features 'area under the curve(AUC) below threshold 65mmHg', 'time-weighted average below threshold 65mmHg','minimum MAP value' [mmHg], and 'cumulative minimum MAP value for 5min' [mmHg] fromthe MAP time series during anesthesia. Six additional featuresrepresenting thedynamics of the MAP time series were extracted, similar to Xue et al.[6] These include general statistical characteristics of the probability density curve over all measured values, i.e., 'mean', 'standard deviation', 'kurtosis', and 'skewness', as well as 'entropy', and 'trend of time series' to reflect measures of irregularity and general value trends within the time series.

The entropy H is a measure for the complexity or disorder of a set of values, or in this case a time series. This variable is generated by calculating the probabilities p of observing a value x within the range of all observed values X in the time series. The entropy of that time series is then given as:[7]

$$H(X) = -\sum_{x \in X} p(x) \log(p(x)). \tag{1}$$

Here, any MAP value that was not present in the time series was omitted and only present values were considered. Since entropy values can reach different maxima, depending on the length of a time series, with longer series amounting to greater maximum values, entropy values were normalized with respect to the length of the corresponding time series to enable comparability across time series of different length.

Skewness is a measure for the symmetry of a probability density function (PDF) over all values in a time series. It is calculated as the tripled distance between mean $\bar{x}$ and median $\tilde{x}$ of MAP values across a time series and normalized by the standard deviation σ of that time series:[8]

$$\text{Skewness}(X) = \frac{3(\bar{x} - \tilde{x})}{\sigma}, \tag{2}$$

It provides a measure of whether values in the curve are distributed symmetrically or if the PDF over the curve is skewed to one side by outliers. A negative skewness would result from a median greater than the mean and therefore imply the presence of

outliers below the mean value as well as a higher count of values above the mean than below and vice versa.
Another measure for the shape of a PDF over the time series is kurtosis or peakedness of that function. Kurtosis β_2 describes the dispersion of values around the mean, relative to the given standard deviation:

$$\beta_2 = \frac{1}{n}\sum_{i=1}^{n}\left(\frac{x_i - \bar{x}}{\sigma}\right)^4, \tag{3}$$

and can be used to determine the occurrence of heavy outliers, or, given a PDF, the tendency to produce such outliers compared to the normal distribution.[8] As kurtosis values cannot be negative and the kurtosis of a normal distribution takes on a value of $\beta_2 = 3$, a kurtosis of $\beta_2 < 3$ is associated with either a leptokurtic PDF, where most values are grouped closely around the mean relative to single large outliers, that give the function a "fat-tailed" appearance. For $\beta_2 > 3$ the shape of the PDF can be described as platykurtic, where outliers occur less prevalent and extreme, compared to a normally distributed PDF.[9]
In order to determine, whether a global trend in the time series exists, Kendall's tau τ was computed as follows:

$$\tau = \frac{C - D}{\sqrt{(n_0 - n_1)(n_0 - n_2)}}, \tag{4}$$

$$n_0 = \frac{n(n-1)}{2}, \tag{5}$$

$$n_1 = \sum_i \frac{t_i(t_i - 1)}{2}, \tag{6}$$

$$n_2 = \sum_j \frac{u_j(u_j - 1)}{2}, \tag{7}$$

with the total number of observations n, the number of discordant D and concordant C pairs in the time series, respectively, and the number of tied values (u_j,t_i) in the respective pairs time t_i and MAP u_j.[10] Concordant and discordant pairs are determined by counting the number of values greater (concordant) and less (discordant) than a value at a certain time point, for all later time points. The individual counts of con- and discordant pairs for each point in time are then added to produce the overall counts. Kendall's tau, therefore, provides an indication whether an overall trend can be detected within a time series, where a strong rising trend would be indicated by a value of τ near 1, and conversely a value close to -1 would result from a strongly declining trend.[11]

1.4 Supplementary File S4: In- and Exclusion of Correlated Predictor Variables

Correlations among potential predictor variables were described by Pearson's correlation coefficient for continuous features, the Phi coefficient for binary features, and Kendall's τ for correlations between binary and multi-level features, respectively. Correlations in continuous-binary and continuous-multinomial pairings were investigated using point bi- and multiserial correlations, respectively. Correlations yielding a respective coefficient above 0.6 are shown in Supplementary Figure 2. For all correlation measures, a cut-off value of 0.8 was chosen, and all feature pairs with a higher absolute correlation were considered for variable exclusion. This was done in order to retain the highest possible amount of information while avoiding strong multicollinearity to influence model performance and feature importance estimation. Diabetes type and the respective binary occurrence variable yielded a correlation coefficient of 0.99. As a result, the diabetes type variable was removed from further analyses and model training. Similarly, the duration of surgery and duration of anesthesia were found to be highly correlated (0.97), leading to the exclusion of surgery duration. Patient weight and BMI were found to be highly correlated as well (0.86), and patient weight was chosen to be excluded from the dataset as a result. Both the pairing of patient sex with height and the Area Under the Curve (ROC-AUC) for MAP time series under 65mmHg with the time-weighted average (TWA) of MAP values under 65mmHg yielded a correlation coefficient of 0.82. As a result, the TWA of MAP below 65mmHg was removed from the dataset, however, both patient sex and height were retained in order to allow the implicit encoding of patient weight contained in the pairing of BMI and height. All remaining correlations were found to be below the chosen cutoff of 0.8 and therefore the respective features were left unchanged within the data set. Additionally, the median expiratory sevofluran measurement was excluded and the maximum expiratory sevofluran measurement was retained to avoid logical redundancy.

1.5 Supplementary File S5: Feature Importance Assessment

Native Model Estimations of Feature Importance Native Feature Importances (FI) were extracted from model architectures in the form of absolute values of feature coefficients (regression models and linear xGBM) or the average contribution of a feature to the total gain of this feature's splits within the model (Random Forest and tree-based xGBM). To enable direct comparison of FIs, values of one-hot encoded levels in multinomial features were averaged to represent the entire feature's importance. Subsequently, absolute FIs were scaled to the largest value for each model. For logistic regression models, absolute values of the *t*-statistic for each feature were used as direct indicator of FI. FI values of one-hot encoded multinomial features were averaged across all levels to allow for direct comparison among different features and algorithms. For Group LASSO models, the respective feature coefficients were directly extracted. The Gini coefficient as a measure of overall impurity decrease caused by splits based on a certain feature over all trees in a forest was used as a representation of FI for RFCs.[12–14] For the SVM classifier, FIs were estimated by fitting a loss smoothing function between predictors and outcomes and using the R^2 statistic as an indicator for a predictor's FI.[12,13] For both xGBM algorithm implementations, FI was estimated as the contribution of each feature to the model. For models with linear base functions, this was represented by the absolute magnitude of a feature's coefficient, and for tree-based models, the averaged contribution of each feature to the total gain of this feature's splits was used to estimate its importance.[12,13]

Shapley Value Estimation of Feature Importance Shapley values were computed for all individual predictions performed by the individual models on the validation data set. The respective individual features contributions to the prediction, as assessed by the Shapley Additive Explanations (SHAP) were averaged over all predictions to estimate overall feature contribution in each model. For multinomial features that were one-hot encoded, values of the individual feature levels were summed to estimate the overall feature's contribution to model prediction. Similar to native FIs, all values were subsequently scaled to the highest contribution per model to allow for a better comparison between models and importance scores.

1.6 Supplementary File S6: Influence of Balanced and Unbalanced Development Cohorts on Model Performance

In the resampling approach, training subsets of the balanced and unbalanced development data sets were exclusively used for hyperparameter optimized training of different prediction models for an initial comparison of approaches. Model performances were evaluated on the test subset of the respective split by calculating and averaging the performances on 100 different train-test-splits within both development data sets. AUC-ROC, sensitivities, specificities, negative predictive values, precision, F1-scores, and BAACs were evaluated for all algorithms in both approaches (Supplementary Table S3). Models trained on subsets of the balanced development data set considerably outperformed those trained on subsets of the unbalanced development data set across all metrics except for specificity. Accordingly, development of the final prediction models was carried out exclusively on the balanced development dataset, leveraging its full sample size. Among the eight different models assessed in the resampling approach, the RFC yielded the best performance with an AUC-ROC of 0·746 (95% Confidence Interval, CI, 0·706 to 0·779), F1-score of 0·678 (95% CI, 0·635 to 0·717), and BACC of 0·680 (95% CI, 0·638 to 0·709). The worst performance was achieved by the linear xGBM with an AUC-ROC of 0·714 (95% CI, 0·679 to 0·745), F1-score of 0·653 (95% CI, 0·611 to 0·694), and BACC of 0·656 (0·623 to 0·690). Similarly to this evaluation, development set dependent performance variance was also assessed for different time points as displayed in Supplementary Table S8.

2 Supplementary Figures

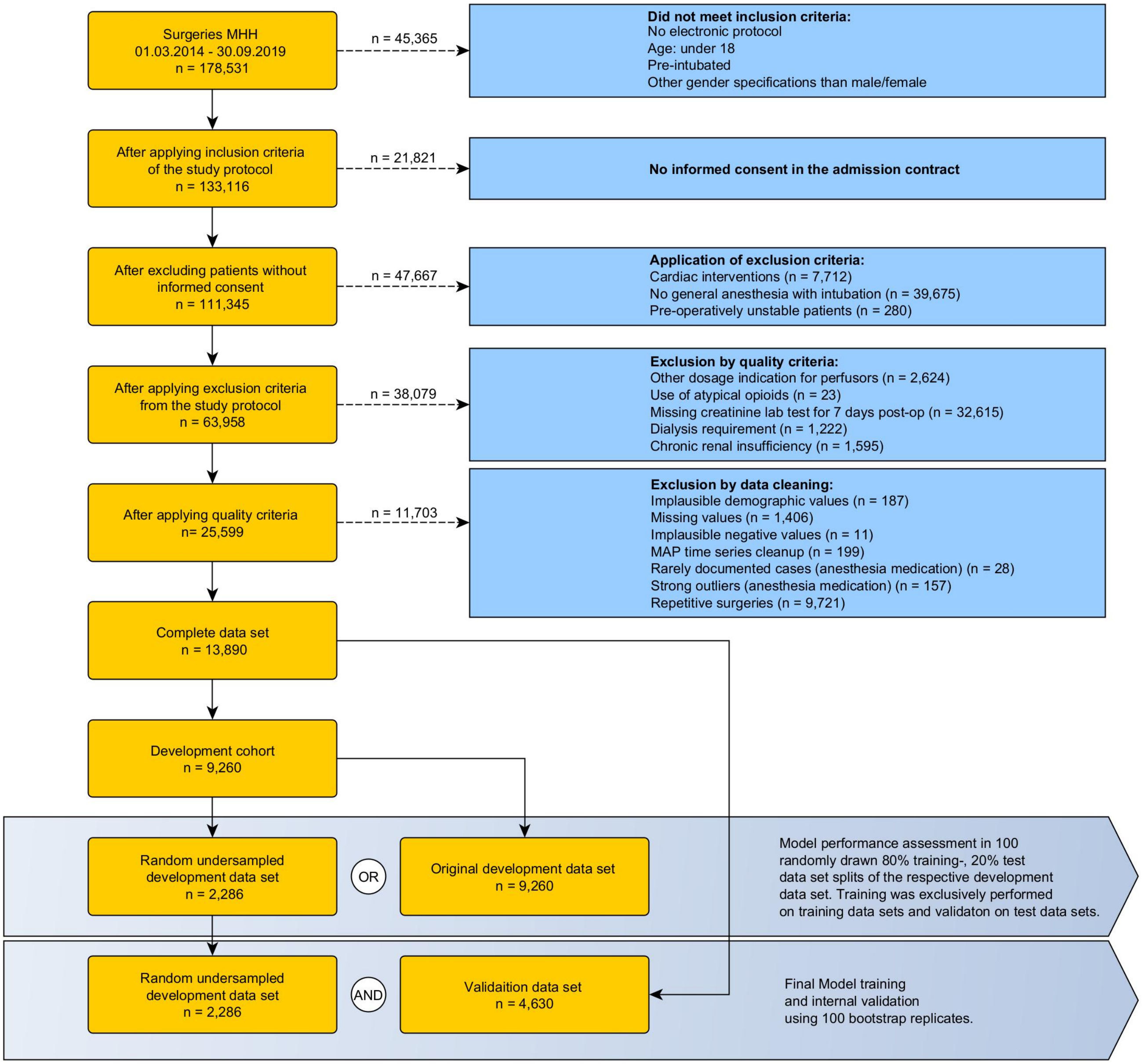

Figure 1. Study population selection. More details are provided in Supplementary File S1.

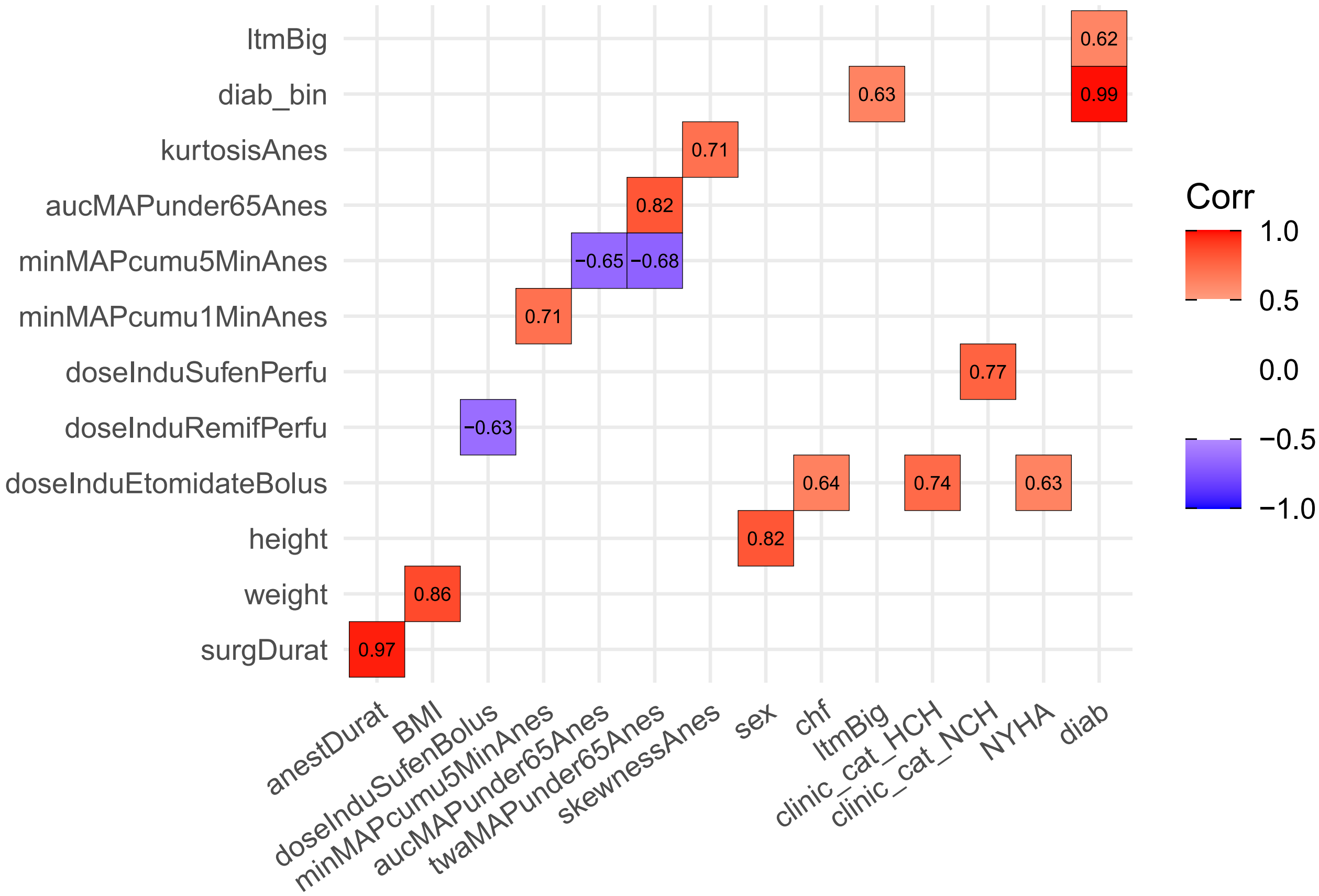

Figure 2. Multicollinearity assessment of predictor variable candidates. Correlation of features in the original development data set, that were found to be strongly (correlation coefficients >0.6) associated with each other, are displayed in a heatmap representation. Positive correlations are shown in red and negative correlations in blue, respectively. Continuous-continuous, binary-binary, ordinal-ordinal, continuous-binary, continuous-ordinal, and binary-ordinal correlations were calculated using the Pearson correlation coefficient, Phi coefficient, Kendall's Tau, Point biserial, and polyserial correlations, respectively. Weak (correlation coefficient <0.6) correlations are omitted. Abbrev.: anestDurat, duration of anesthesia; aucMAPunder65Anes, Area under the Curve for all MAP values below 65mmHg during anesthesia; BMI, body mass index of patient; chf, chronic heart failure; clinic_cat_HCH, surgery in dermatology department (tumor removal); clinic_cat_NCH, surgery in neurology department; DECREASE, Dutch Echocardiographic Cardiac Risk Evaluation Applying Stress Echo score; diab, diabetes (types); diab_bin, diabetes (yes/no); doseInduEtomidateBolus, dose of etomidate administered via bolus during induction; doseInduRemifPerfu, dose of remifentanil administered via perfusor during induction; doseInduSufenPerfu, dose of sufentanil administered via perfusor during induction; doseInduSufenBolus, dose of sufentanil administered via bolus during induction; eGFRPreSurg, estimated glomerular filtration rate prior to surgery; height, patient height; kurtosisAnes, kurtosis of MAP values during anesthesia; ltmBig, longterm medication with biguanides; minMAPcumu1MinAnes, minimal MAP value for 1min during anesthesia; minMAPcumu5MinAnes, minimal MAP value for 5min during anesthesia; NYHA, New York Heart Association classification of heart disease; RCRI, Revised Cardiac Risk Index; sex, biological sex of patient; skewnessAnes, skewness of MAP values during anesthesia; surgDurat, duration of surgical intervention; twaMAPunder65Anes, time-weighted average for all MAP values below 65mmHg during anesthesia; weight, patient weight.

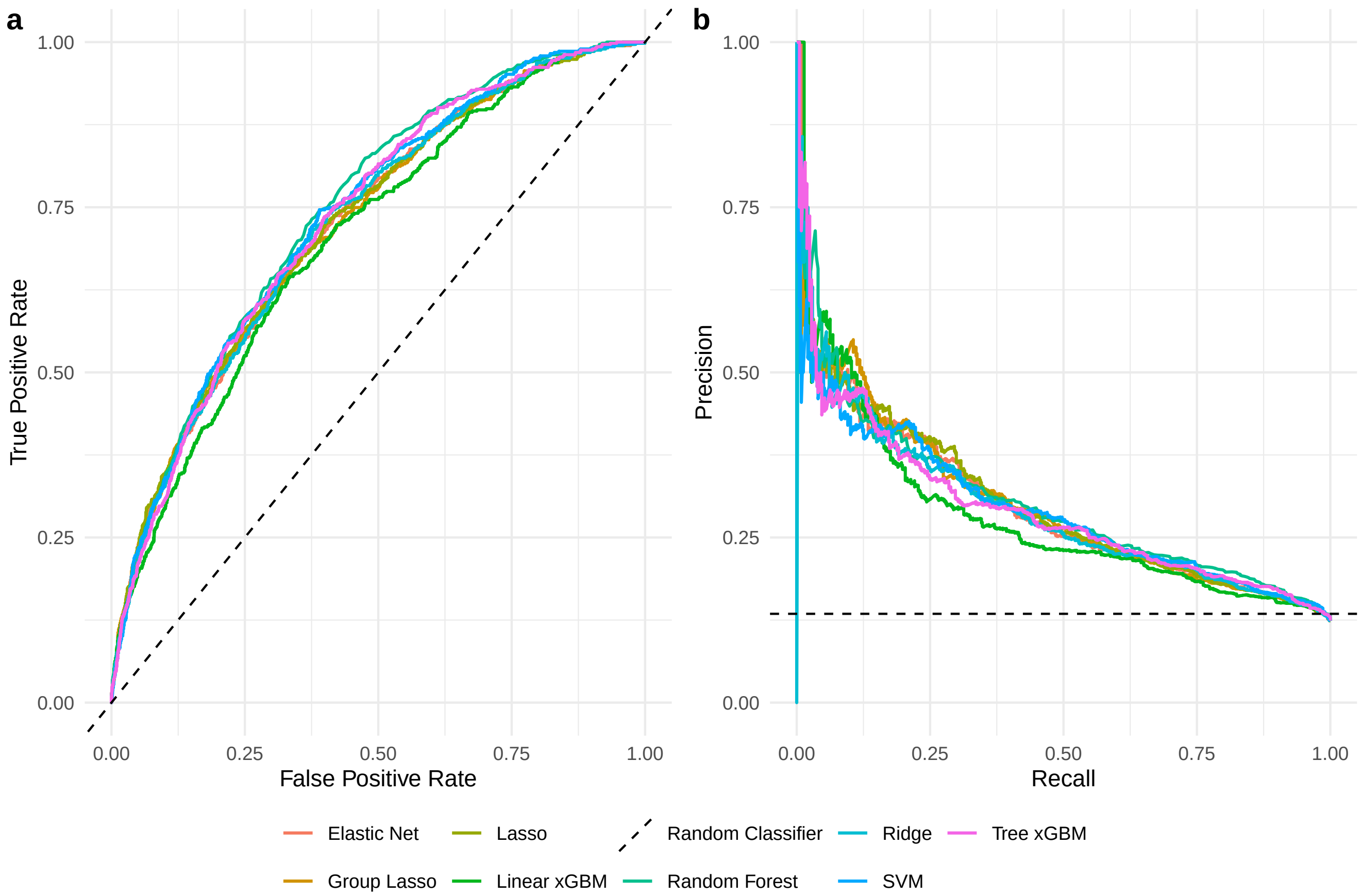

Figure 3. **(a) Receiver Operating Characteristics and (b) Precision-Recall curves of machine learning models trained on the balanced development data set and evaluated on the validation data set.** The dashed lines indicate the expected curves for a random classifier, given for the Precision-Recall curve by the outcome distribution within the validation dataset (12.42% PO-AKI occurrence).
Abbrev.: SVM, Support Vector Machine classifier; xGBM, extreme Gradient Boosting Machine classifier.

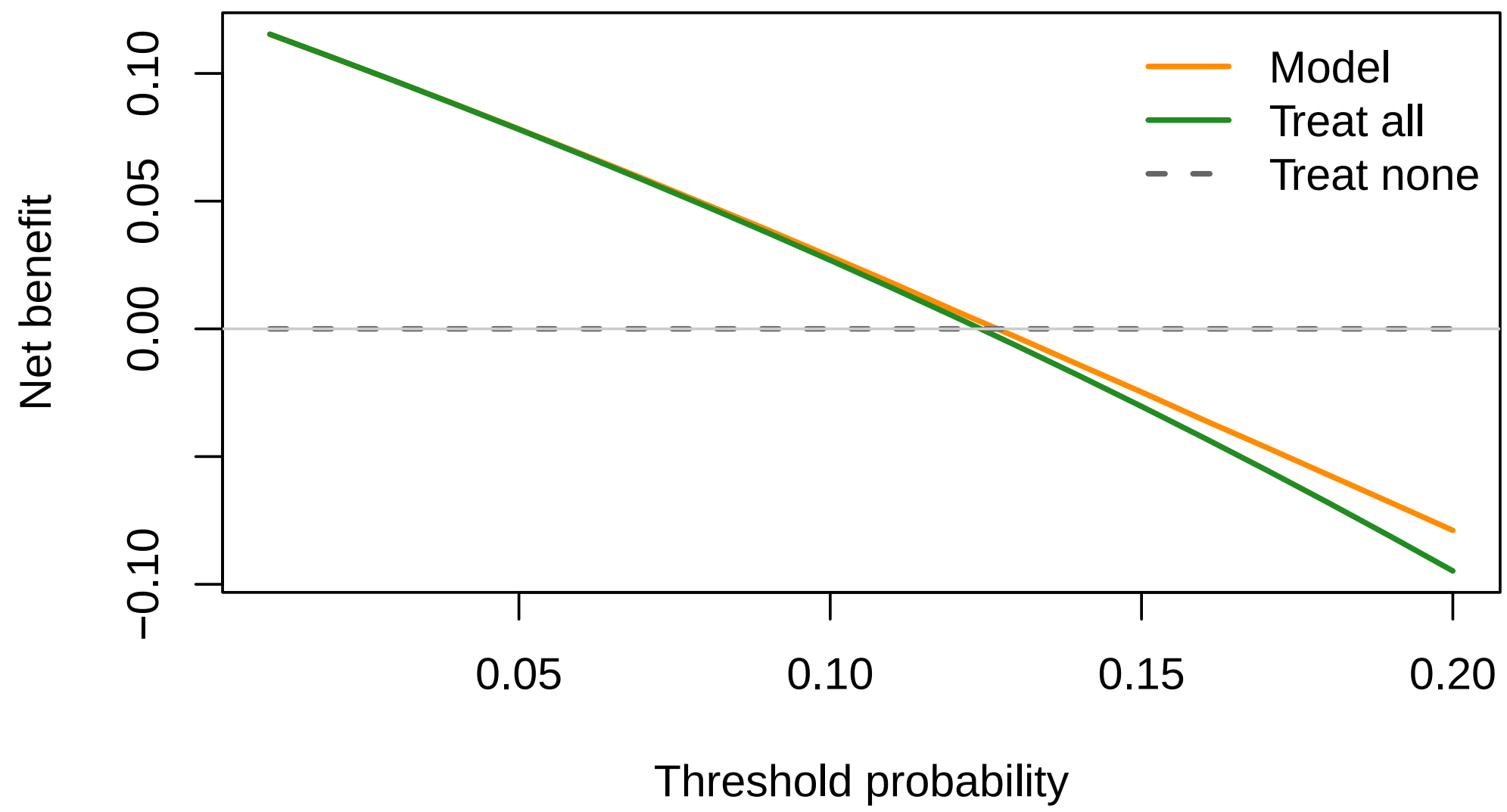

Figure 4. Decision Curve Analysis of best performing RFC model. Net benefit (y-axis) is shown for Treat all (green), Treat none (dashed) and the pure model prediction (orange), for a range of decision thresholds (x-axis) between 0.01 and 0.2. For all other thresholds, the net benefit of all three approaches remained at or below zero.

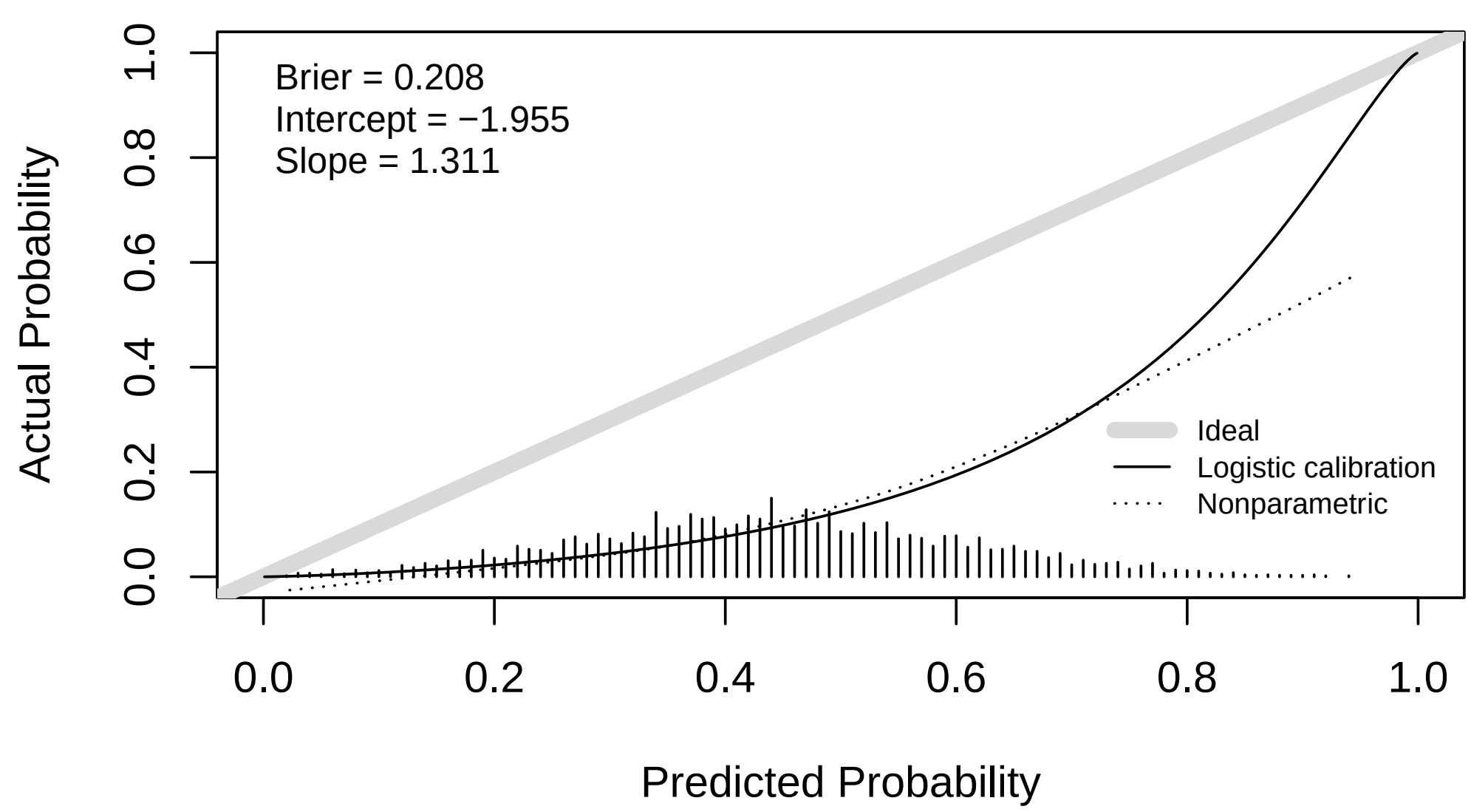

Figure 5. Calibration curve of best performing RFC model on the full validation data set. Nonparametric (dotted) and logistic (line) calibration curve display the relationship of predicted (x-axis) and actual (y-axis) probabilities, compared to an ideal model (grey). The distribution of predicted performances is displayed as histogram. Calibration Intercept, calibration slope and Brier score are supplied in the upper left corner.

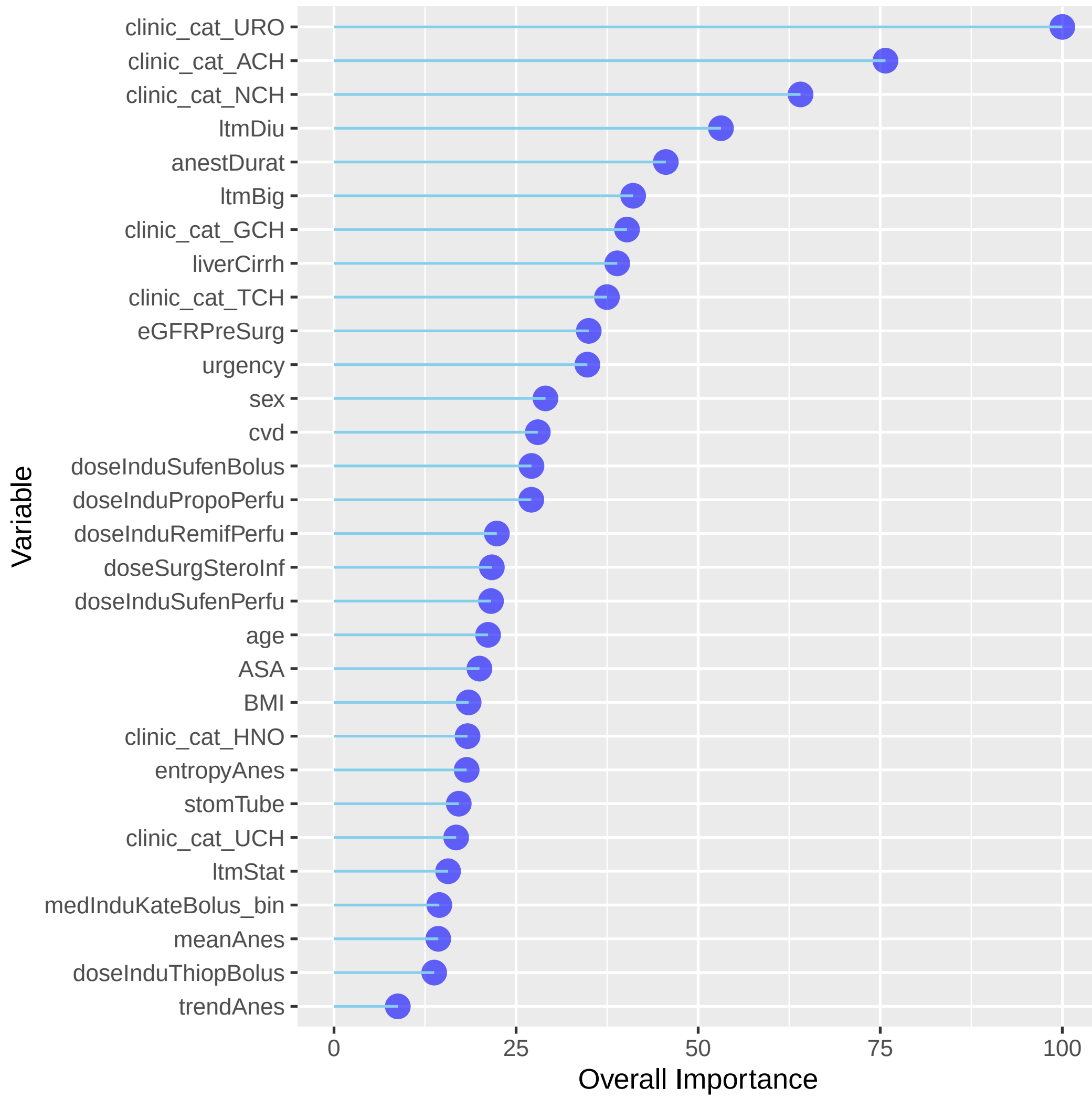

Figure 6. Scaled native Feature Importances (FIs) of the 30 most important predictors, assigned by a logistic Group-LASSO regression model trained on the complete balanced development cohort for the prediction of post-operative acute kidney injury after non-cardiac surgery. FI measures are based on native feature weights within the model equation. All weights are scaled to the highest FI, which is assigned a value of 100, for better comparability. Abbrev.: age, patient age; anestDurat, duration of anesthesia; ASA, American Society of Anesthesiology physical status; BMI, Body Mass Index of patient; clinic_cat_ACH, general surgery; clinic_cat_GCH, vascular surgery; clinic_cat_HNO, otolaryngology surgery; clinic_cat_NCH, neurology surgery; clinic_cat_TCH, thoracic surgery; clinic_cat_UCH, trauma surgery; clinic_cat_URO, urology surgery; cvd, cerebrovascular disease; doseInduPropoPerfu, dose of propofol administered via perfusor during induction; doseInduRemifPerfu, dose of remifentanil administered via perfusor during induction; doseInduSufenBolus, dose of sufentanil administered via bolus during induction; doseInduSufenPerfu, dose of sufentanil administered via perfusor during induction; doseInduThiopBolus, dose of thiopental administered via bolus during induction; doseSurgSteroInf, dose of sterofundin administered via infusion during induction; eGFRPreSurg, estimated glomerular filtration rate before surgery; entropyAnes, entropy of MAP during anesthesia; liverCirrh, liver cirrhosis; ltmBig, longterm medication with biguanides; ltmDiu, longterm medication with diuretics; ltmStat, longterm medication with statins; medInduKateBolus_bin, administration of catecholamine during induction; meanAnes, mean of MAP during anesthesia; sex, biological sex of patient; stomTube, stomach tube; trendAnes, trend of MAP during anesthesia; urgency, level of urgency of surgical intervention.

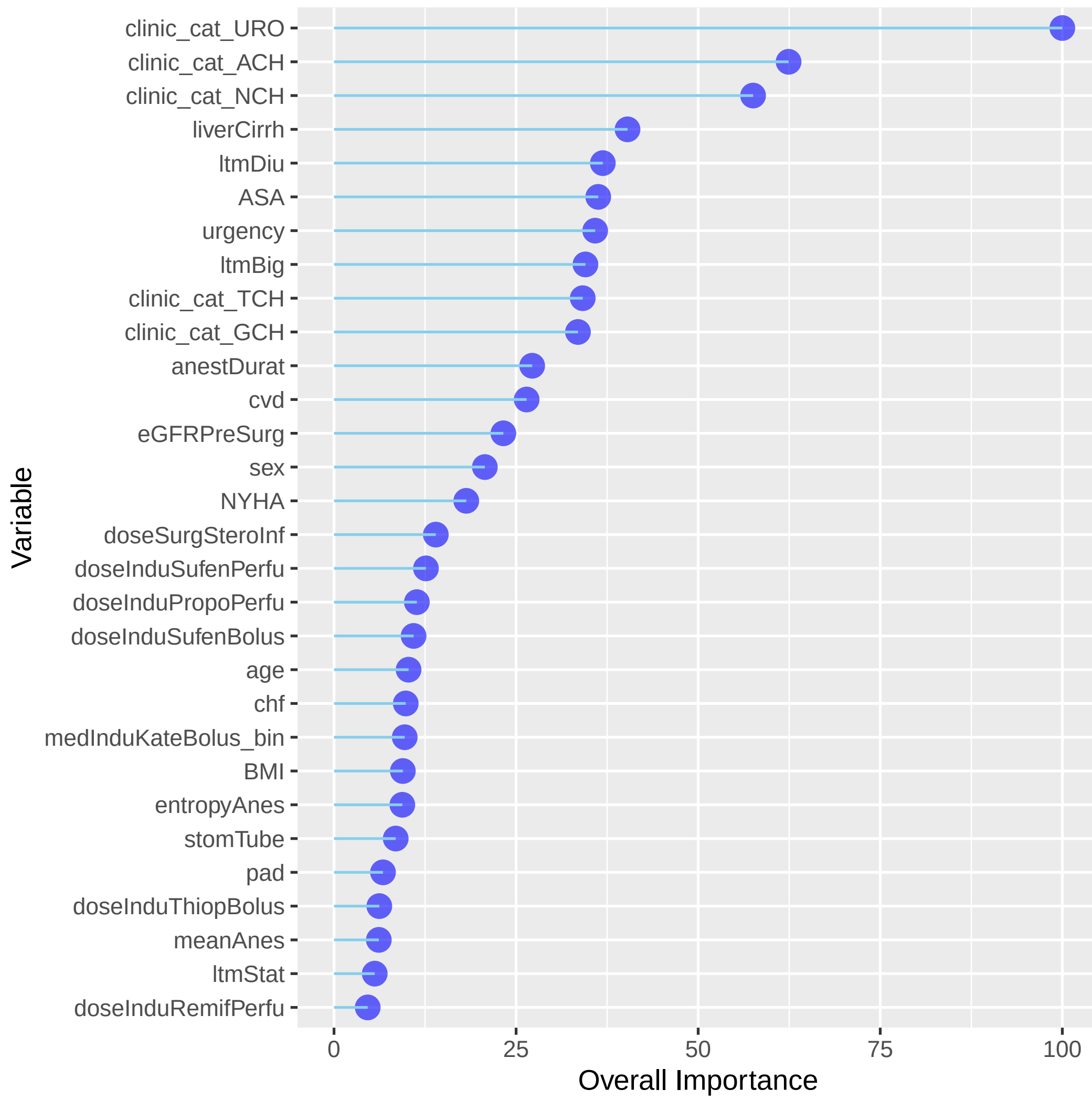

Figure 7. Scaled native Feature Importances (FIs) of the 30 most important predictors, assigned by a logistic LASSO regression model trained on the complete balanced development cohort for the prediction of post-operative acute kidney injury after non-cardiac surgery. FI measures are based on native feature weights within the model equation. FIs of one-hot encoded multinomial features were calculated as the mean of all individual levels' FI values. All weights are scaled to the highest FI, which is assigned a value of 100, for better comparability.
Abbrev.: age, patient age; anestDurat, duration of anesthesia; ASA, American Society of Anesthesiology physical status; BMI, Body Mass Index of patient; cvd, cerebrovascular Disease; chf, chronic heart failure; clinic_cat_ACH, general surgery; clinic_cat_GCH, vascular surgery; clinic_cat_NCH, neurology surgery; clinic_cat_TCH, Thoracic surgery; clinic_cat_URO, urology surgery; doseInduPropoPerfu, dose of propofol administered via perfusor during induction; doseInduRemifPerfu, dose of remifentanil administered via perfusor during induction; doseInduSufenBolus, dose of sufentanil administered via bolus during induction; doseInduSufenPerfu, dose of sufentanil administered via perfusor during induction; doseInduThiopBolus, dose of thiopental administered via bolus during induction; doseSurgSteroInf, dose of sterofundin administered via infusion during induction; eGFRPreSurg, estimated glomerular filtration rate before surgery; entropyAnes, entropy of MAP during anesthesia; liverCirrh, liver cirrhosis; ltmBig, longterm medication with biguanides; ltmDiu, longterm medication with diuretics; ltmStat, longterm medication with statins; meanAnes; Mean of MAP during anesthesia; medInduKateBolus_bin, administration of catecholamine during induction; NYHA, New York Heart Association classification of heart disease; pad, peripheral arterial disease; sex, biological sex of patient; stomTube, stomach tube; urgency, level of urgency of surgical intervention.

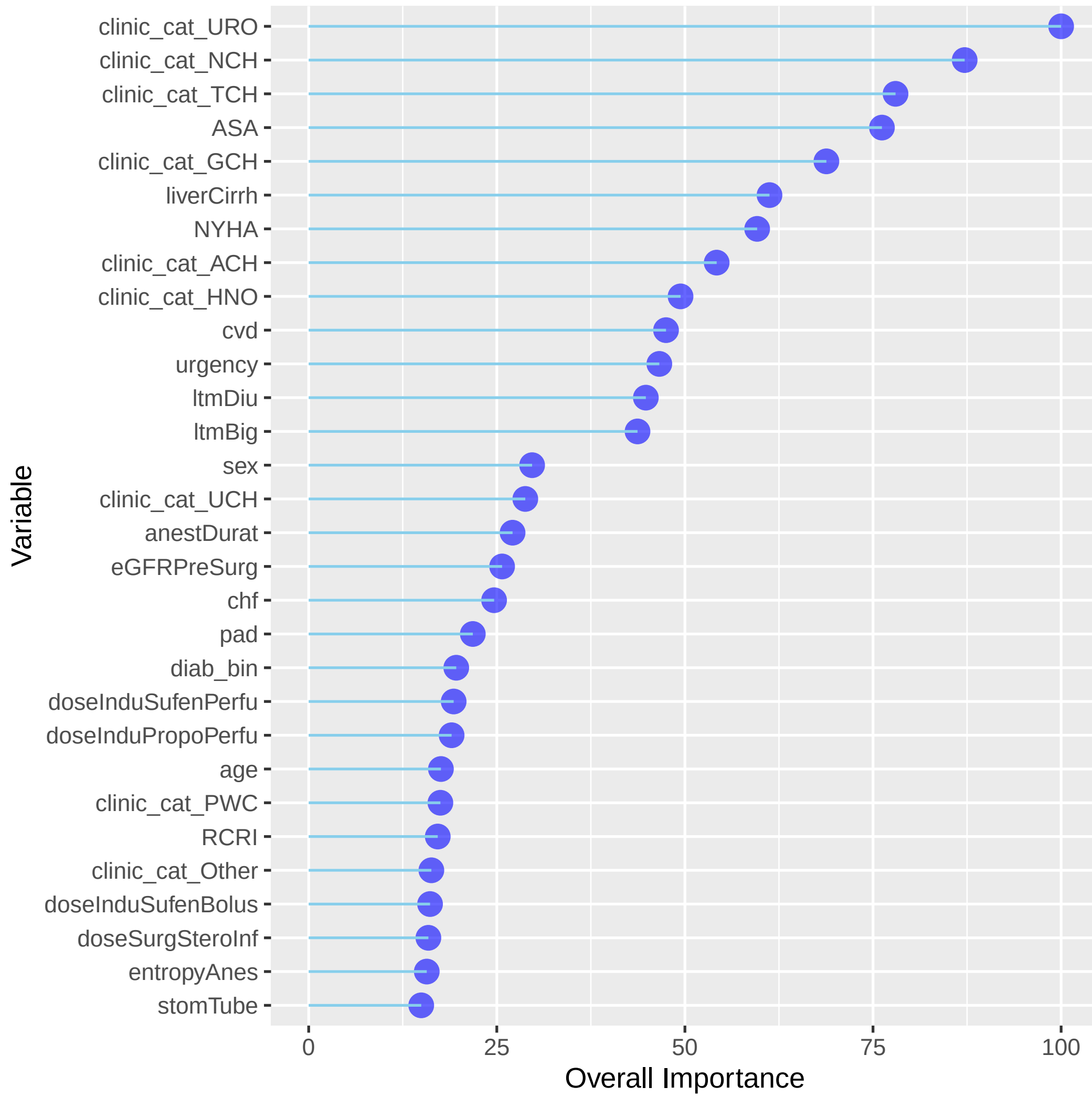

Figure 8. Scaled native Feature Importances (FIs) of the 30 most important predictors, assigned by a logistic Ridge regression model trained on the complete balanced development cohort for the prediction of post-operative acute kidney injury after non-cardiac surgery. FI measures are based on native feature weights within the model equation. FIs of one-hot encoded multinomial features were calculated as the mean of all individual levels' FI values. All weights are scaled to the highest FI, which is assigned a value of 100, for better comparability.
Abbrev.: age, patient age; ASA, American Society of Anesthesiology physical status; anestDurat, duration of anesthesia; chf, chronic heart failure; clinic_cat_ACH, general surgery; clinic_cat_GCH, vascular surgery; clinic_cat_HNO, otolaryngology surgery; clinic_cat_NCH, neurology surgery; clinic_cat_Other, other surgery department; clinic_cat_PWC, plastic surgery; clinic_cat_TCH, thoracic surgery; clinic_cat_UCH, trauma surgery; clinic_cat_URO, urology surgery; cvd, cerebrovascular disease; diab_bin, diabetes (yes/no); doseInduPropoPerfu, dose of propofol administered via perfusor during induction; doseInduSufenBolus, dose of sufentanil administered via bolus during induction; doseInduSufenPerfu, dose of sufentanil administered via perfusor during induction; doseSurgSteroInf, dose of sterofundin administered via infusion during induction; eGFRPreSurg, estimated glomerular filtration rate before surgery; entropyAnes, entropy of MAP during anesthesia; liverCirrh, liver cirrhosis; ltmBig, longterm medication with biguanides; ltmDiu, longterm medication with diuretics; NYHA, New York Heart Association classification of heart disease; pad, peripheral arterial disease; RCRI, revised cardiac risk index; sex, biological sex of patient; stomTube, stomach tube; urgency, level of urgency of surgical intervention.

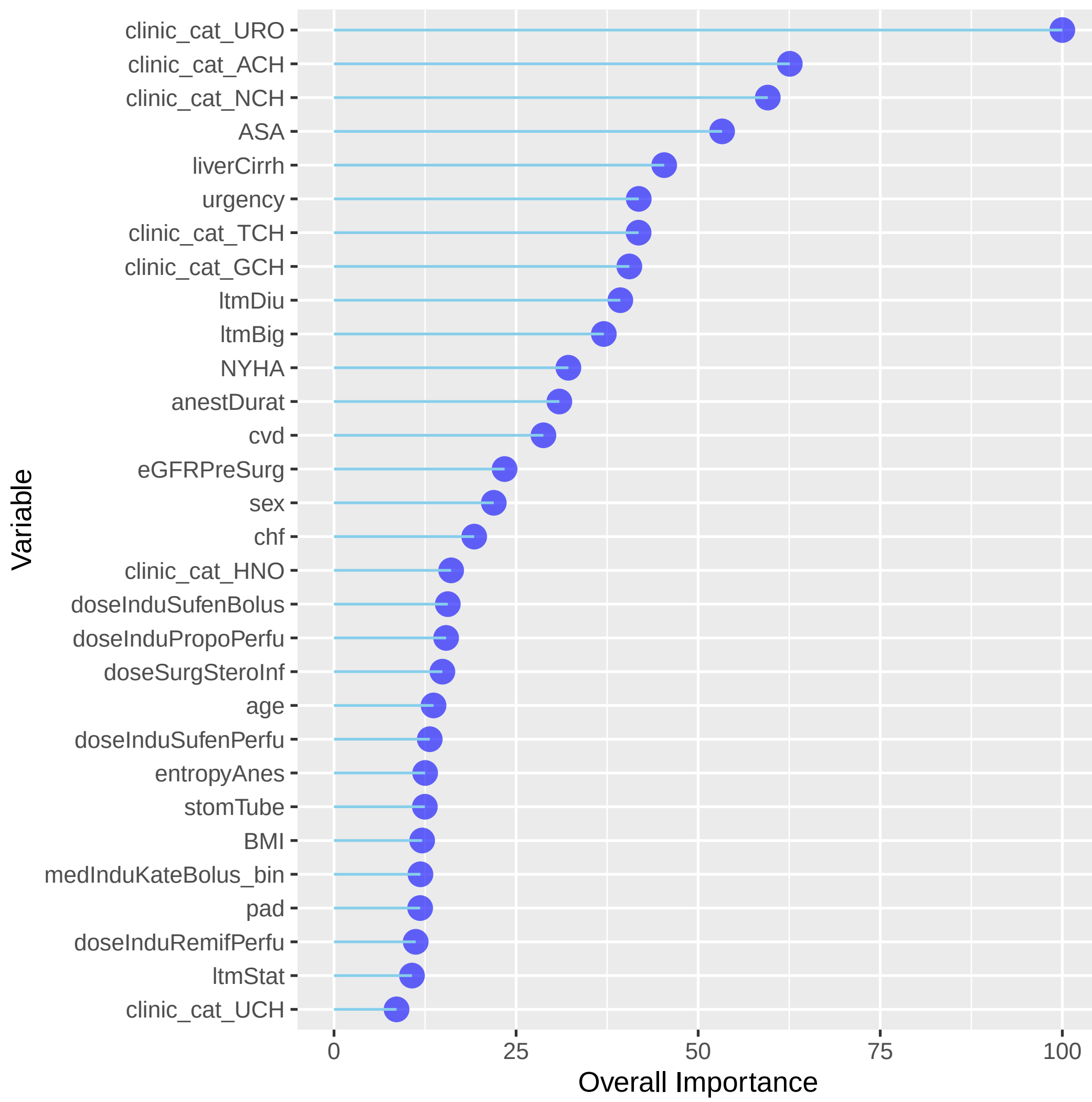

Figure 9. Scaled native Feature Importances (FIs) of the 30 most important predictors, assigned by a logistic Elastic Net regression model trained on the complete balanced development cohort for the prediction of post-operative acute kidney injury after non-cardiac surgery. FI measures are based on native feature weights within the model equation. FIs of one-hot encoded multinomial features were calculated as the mean of all individual levels' FI values. All weights are scaled to the highest FI, which is assigned a value of 100, for better comparability.
Abbrev.: age, patient age; anestDurat, duration of anesthesia; ASA, American Society of Anesthesiology physical status; BMI, Body Mass Index of patient; chf, chronic heart failure ; clinic_cat_ACH, general surgery; clinic_cat_GCH, gynaecology surgery; clinic_cat_HNO, otolaryngology surgery; clinic_cat_NCH, neurology surgery; clinic_cat_TCH, thoracic surgery; clinic_cat_UCH, trauma surgery; clinic_cat_URO, urology surgery; cvd, cerebrovascular disease; doseInduPropoPerfu, dose of propofol administered via perfusor during induction; doseInduRemifPerfu, dose of remifentanil administered via perfusor during induction; doseInduSufenBolus, dose of sufentanil administered via bolus during induction; doseInduSufenPerfu, dose of sufentanil administered via perfusor during induction; doseSurgSteroInf, dose of sterofundin administered via infusion during induction; eGFRPreSurg, estimated glomerular filtration rate before surgery; entropyAnes, entropy of MAP during anesthesia; liverCirrh, liver cirrhosis; ltmBig, longterm medication with biguanides; ltmDiu, longterm medication with diuretics; ltmStat, longterm medication with statins; medInduKateBolus_bin, administration of catecholamine during induction; NYHA, New York Heart Association classification of heart disease; pad, peripheral arterial disease; sex, biological sex of patient; stomTube, stomach tube; urgency, level of urgency of surgical intervention.

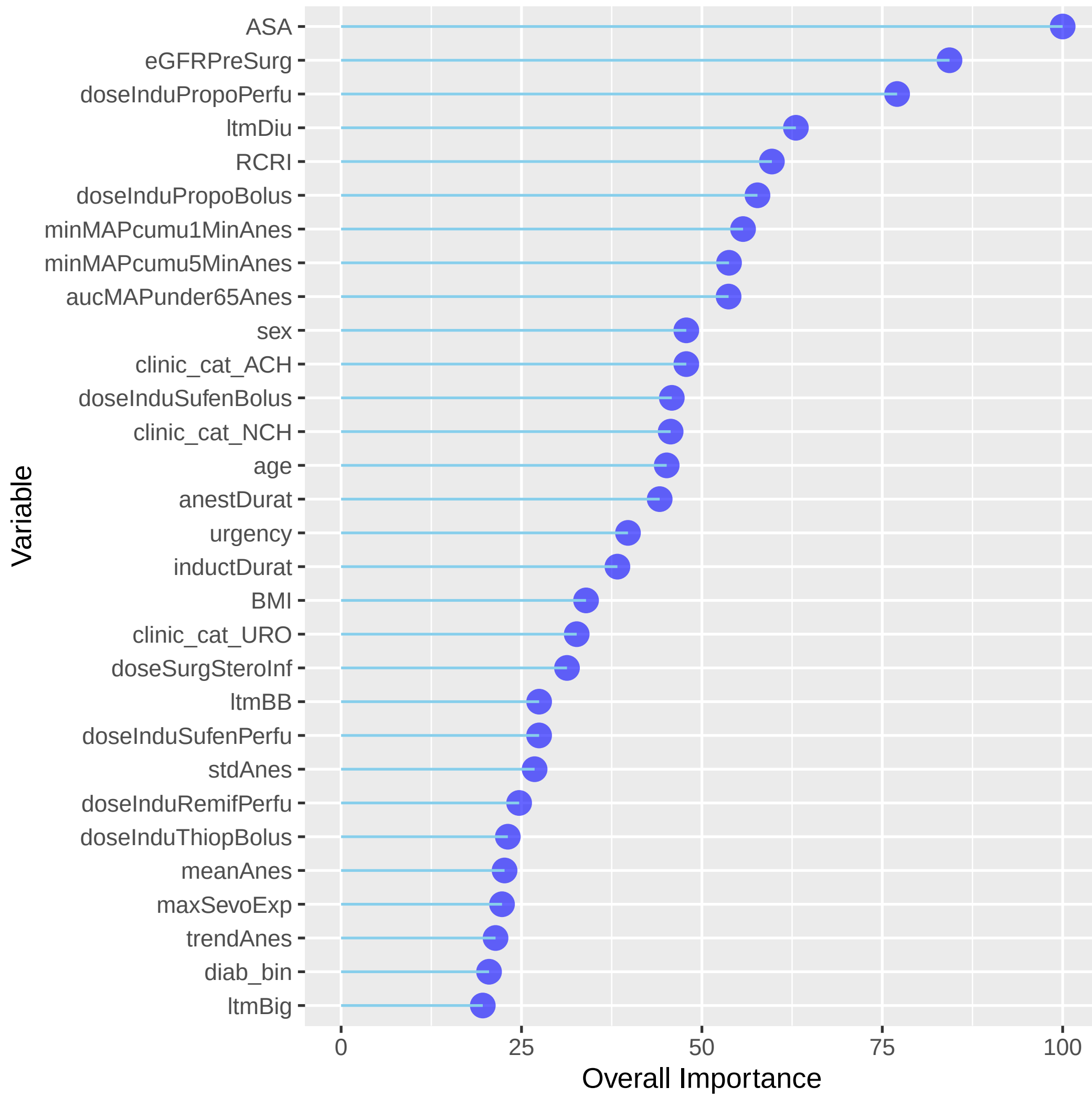

Figure 10. Scaled native Feature Importances (FIs) of the 30 most important predictors, assigned by a Support Vector Machine using a radial kernel function, trained on the complete balanced development cohort for the prediction of post-operative acute kidney injury after non-cardiac surgery. FI measures were computed by fitting a loss smoothing function between predictors and prediction outcomes and using the R^2 statistic as an indicator for a predictor's FI. All weights are scaled to the highest FI, which is assigned a value of 100, for better comparability.
Abbrev.: age, patient age; anestDurat, duration of anesthesia; ASA, American Society of Anesthesiology physical status; aucMAPunder65Anes, area under the curve for all MAP below 65mmHg during anesthesia; BMI, body mass index of patient; clinic_cat_ACH, general surgery; clinic_cat_NCH, neurology surgery; clinic_cat_URO, urology surgery; diab_bin, diabetes (yes/no); doseInduPropoBolus, dose of propofol administered via bolus during induction; doseInduPropoPerfu, dose of propofol administered via perfusor during induction; doseInduRemifPerfu, dose of remifentanil administered via perfusor during induction; doseInduSufenBolus, dose of sufentanil administered via bolus during induction; doseInduSufenPerfu, dose of sufentanil administered via perfusor during induction; doseInduThiopBolus, dose of thiopental administered via perfusor during induction; doseSurgSteroInf, dose of sterofundin administered via infusion during induction; eGFRPreSurg, estimated glomerular filtration rate before surgery; inductDurat, duration of induction of surgery; ltmBB, longterm medication with beta blockers; ltmBig, longterm medication with biguanides; ltmDiu, longterm medication with diuretics; maxSevoExp, maximum expiratory sevoflurane measurement during induction; meanAnes, Mean of MAP during anesthesia; minMAPcumu1MinAnes, minimal MAP for 1 minute during anesthesia; minMAPcumu5MinAnes, minimal MAP for 5 minutes during anesthesia; RCRI, revised cardiac risk index; sex, biological sex of patient; stdAnes, standard deviation of MAP during anesthesia; trendAnes, trend of MAP during anesthesia; urgency, level of urgency of surgical intervention.

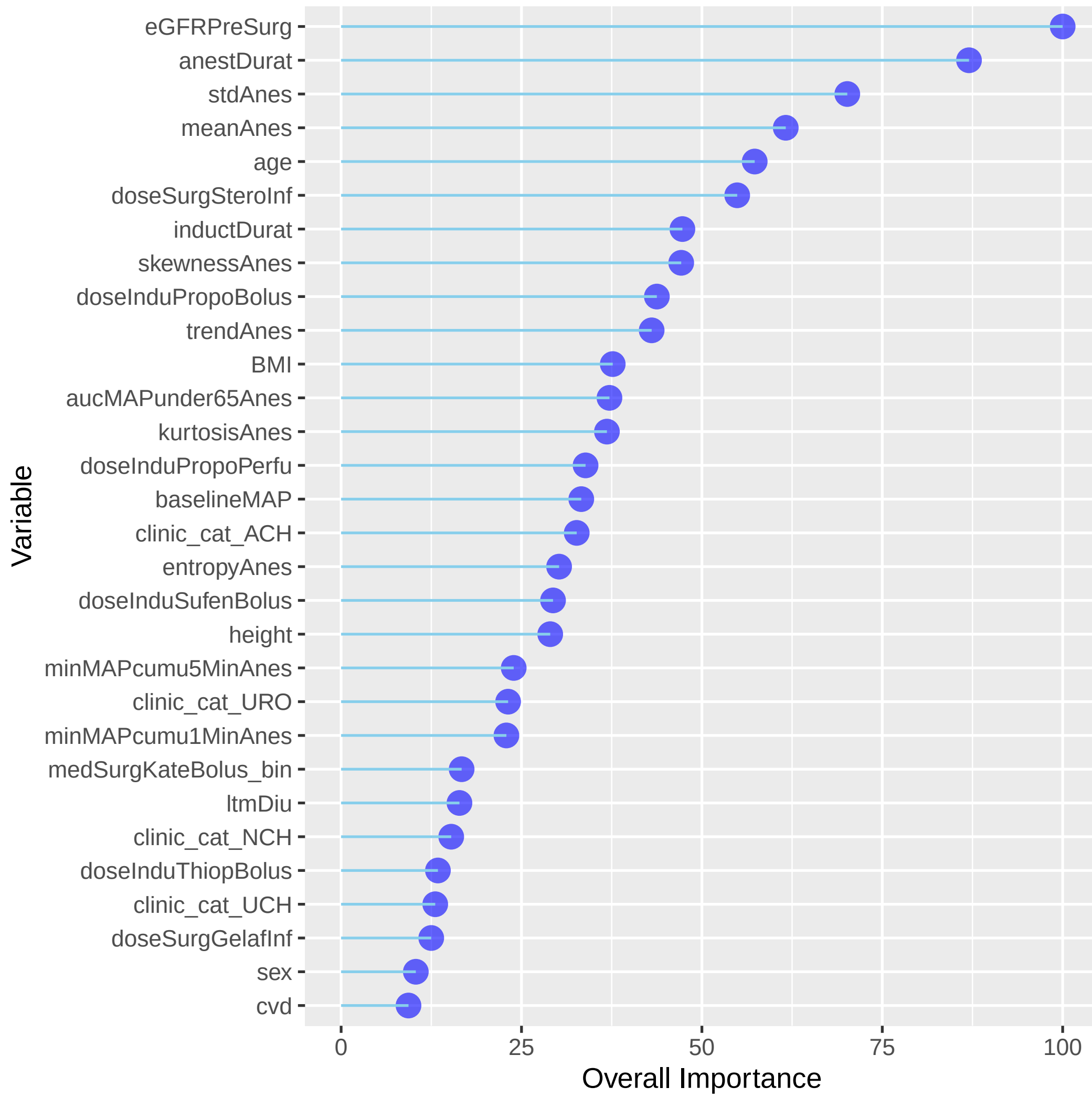

Figure 11. Scaled native Feature Importances (FIs) of the 30 most important predictors, assigned by an extreme Gradient Boosting Machine with a linear regression algorithm as base learner, trained on the complete balanced development cohort for the prediction of post-operative acute kidney injury after non-cardiac surgery. FI measures are given by the absolute magnitude of a feature's coefficient within the model's equation. All weights are scaled to the highest FI, which is assigned a value of 100, for better comparability.
Abbrev.: age, patient age; anestDurat, duration of anesthesia; aucMAPunder65Anes, area under the curve for all MAP below 65mmHg during anesthesia; baselineMAP, first measured MAP value; BMI, body mass index of patient; clinic_cat_ACH, general surgery; clinic_cat_NCH, neurology surgery; clinic_cat_UCH, trauma surgery; clinic_cat_URO, urology surgery; cvd, cerebrovascular disease; doseInduPropoBolus, dose of propofol administered via bolus during induction; doseInduPropoPerfu, dose of propofol administered via perfusor during induction; doseInduSufenBolus, dose of sufentanil administered via bolus during induction; doseInduThiopBolus, dose of thiopental administered via bolus during induction; doseSurgGelafInf, dose of gelafundin administered via infusion during induction; doseSurgSteroInf, dose of sterofundin administered via infusion during induction; eGFRPreSurg, estimated glomerular filtration rate before surgery; entropyAnes, entropy of MAP during anesthesia; height, patient height; inductDurat, duration of induction of surgery; kurtosisAnes, kurtosis of MAP during anesthesia; ltmDiu, longterm medication with diuretics; meanAnes, mean MAP during entire anesthesia; medSurgKateBolus_bin, administration of catecholamine during surgery; minMAPcumu1MinAnes, minimal MAP for 1 minute during anesthesia; minMAPcumu5MinAnes, minimal MAP for 5 minutes during anesthesia; sex, biological sex of patient; skewnessAnes, skewness of MAP during anesthesia; stdAnes, standard deviation of MAP during anesthesia; trendAnes, trend of MAP during anesthesia.

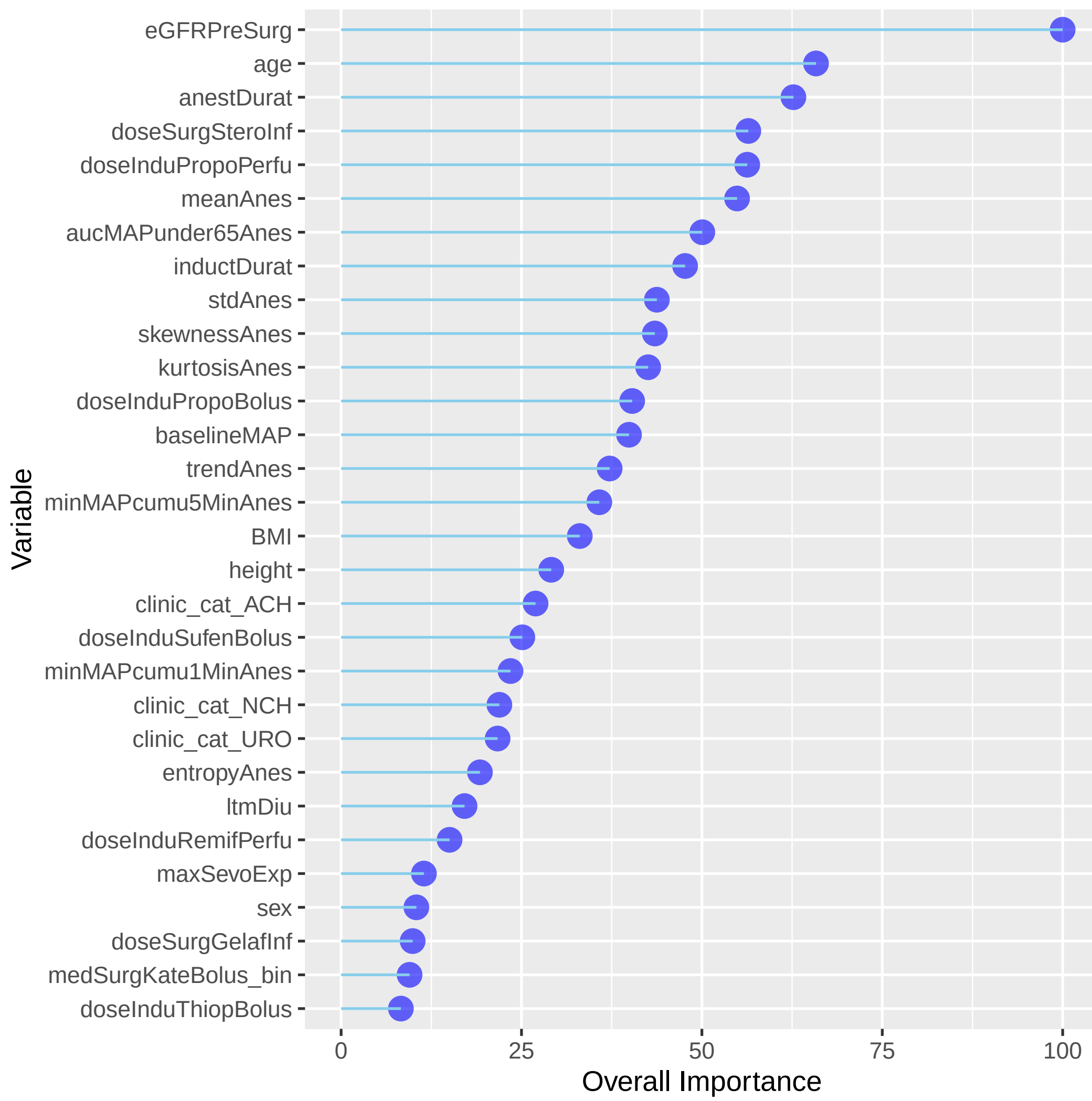

Figure 12. Scaled native Feature Importances (FIs) of the 30 most important predictors, assigned by an extreme Gradient Boosting Machine with a Decision Tree algorithm as base learner, trained on the complete balanced development cohort for the prediction of post-operative acute kidney injury after non-cardiac surgery. FI measures are based on the averaged contribution of each feature to the total gain of this feature's splits. All weights are scaled to the highest FI, which is assigned a value of 100, for better comparability.
Abbrev.: age, patient age; anestDurat, duration of anesthesia; aucMAPunder65Anes, area under the curve for all MAP below 65mmHg during anesthesia; baselineMAP, first measured MAP value; BMI, Body Mass Index of patient; clinic_cat_ACH, general surgery; clinic_cat_NCH, neurology surgery; clinic_cat_URO, urology surgery; doseInduPropoBolus, dose of propofol administered via bolus during induction; doseInduPropoPerfu, dose of propofol administered via perfusor during induction; doseInduRemifPerfu, dose of remifentanil administered via perfusor during induction; doseInduSufenBolus, dose of sufentanil administered via bolus during induction; doseInduThiopBolus, dose of thiopental administered via bolus during induction; doseSurgGelafInf, dose of gelafundin administered via infusion during induction; doseSurgSteroInf, dose of sterofundin administered via infusion during induction; eGFRPreSurg, estimated glomerular filtration rate before surgery; entropyAnes, entropy of MAP during anesthesia; height, patient height; inductDurat, duration of induction of surgery; kurtosisAnes, kurtosis of MAP during anesthesia; ltmDiu, longterm medication with diuretics; maxSevoExp, maximum expiratory sevoflurane measurement during induction; meanAnes, mean MAP during entire anesthesia; medSurgKateBolus_bin, administration of catecholamine during surgery; minMAPcumu1MinAnes, minimal MAP for 1 minute during anesthesia; minMAPcumu5MinAnes, minimal MAP for 5 minutes during anesthesia; sex, biological sex of patient; skewnessAnes, skewness of MAP during anesthesia; stdAnes, standard deviation of MAP during anesthesia; trendAnes, trend of MAP during anesthesia.

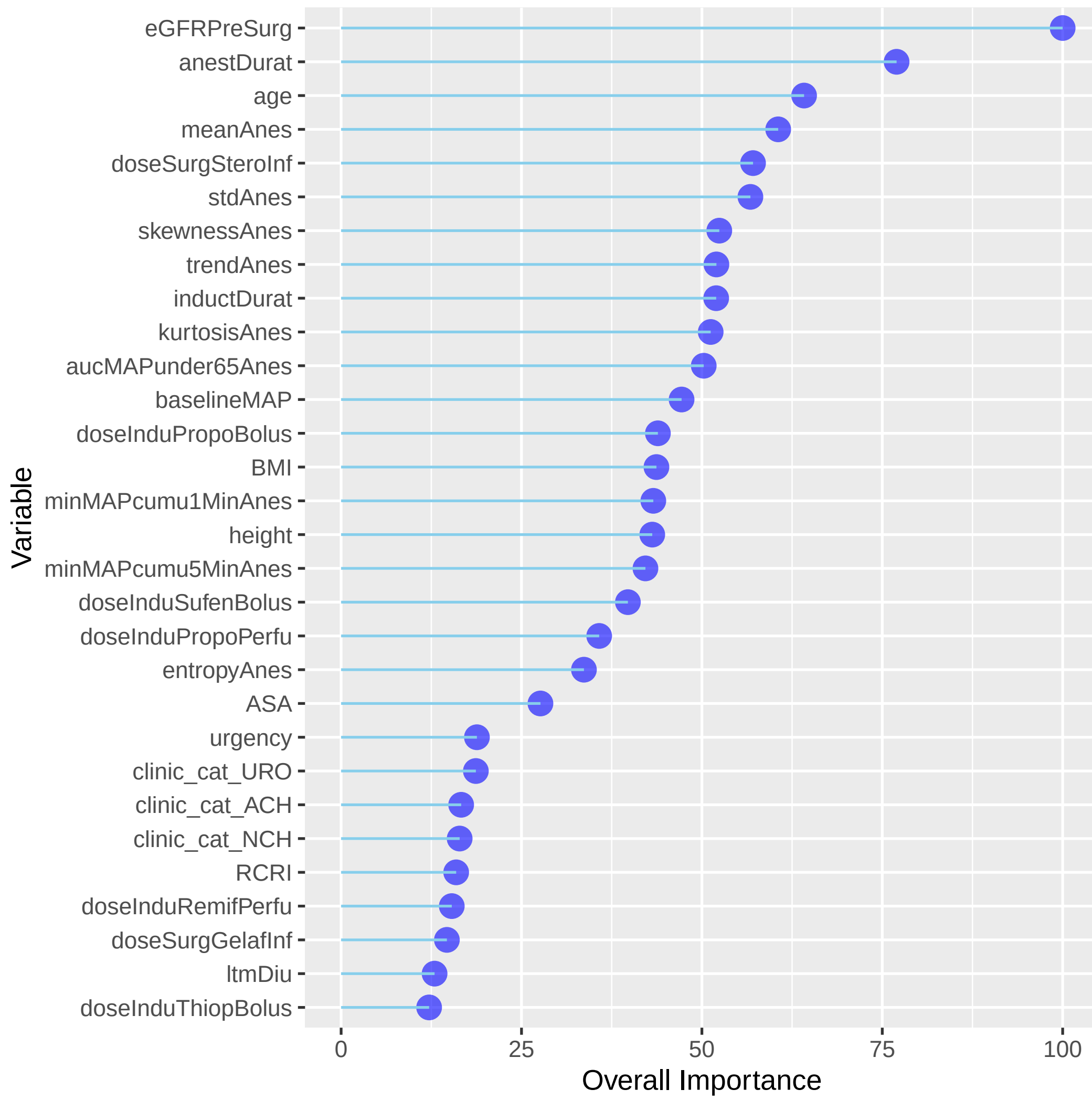

Figure 13. **Scaled native Feature Importances (FIs) of the 30 most important predictors, assigned by a Random Forest model trained on the complete balanced development cohort for the prediction of post-operative acute kidney injury after non-cardiac surgery.** FI measures are based on the contribution of all splits, where the feature is used, to the Gini-coefficient. All weights are scaled to the FI of the most important feature for better comparability. Abbrev.: age, patient age; anestDurat, duration of anesthesia; ASA, American Society of Anesthesiology physical status; aucMAPunder65Anes, area under the curve for all MAP below 65mmHg during anesthesia; baselineMAP, first measured MAP value; BMI, body mass index of patient; clinic_cat_ACH, general surgery; clinic_cat_NCH, neurology surgery; clinic_cat_URO, urology surgery; doseInduPropoBolus, dose of propofol administered via bolus during induction; doseInduPropoPerfu, dose of propofol administered via perfusor during induction; doseInduRemifPerfu, dose of remifentanil administered via perfusor during induction; doseInduSufenBolus, dose of sufentanil administered via bolus during induction; doseInduThiopBolus, dose of thiopental administered via bolus during induction; doseSurgGelafInf, dose of gelafundin administered via infusion during induction; doseSurgSteroInf, dose of sterofundin administered via infusion during induction; eGFRPreSurg, estimated glomerular filtration rate before surgery; entropyAnes, entropy of MAP during anesthesia; height, patient height; inductDurat, duration of induction of surgery; kurtosisAnes, kurtosis of MAP during anesthesia; ltmDiu, longterm medication with diuretics; meanAnes, mean MAP during entire anesthesia; minMAPcumu1MinAnes, minimal MAP for 1 minute during anesthesia; minMAPcumu5MinAnes, minimal MAP for 5 minutes during anesthesia; RCRI, revised cardiac risk index; skewnessAnes, skewness of MAP during anesthesia; stdAnes, standard deviation of MAP during anesthesia; trendAnes, trend of MAP during anesthesia; urgency, level of urgency of surgical intervention.

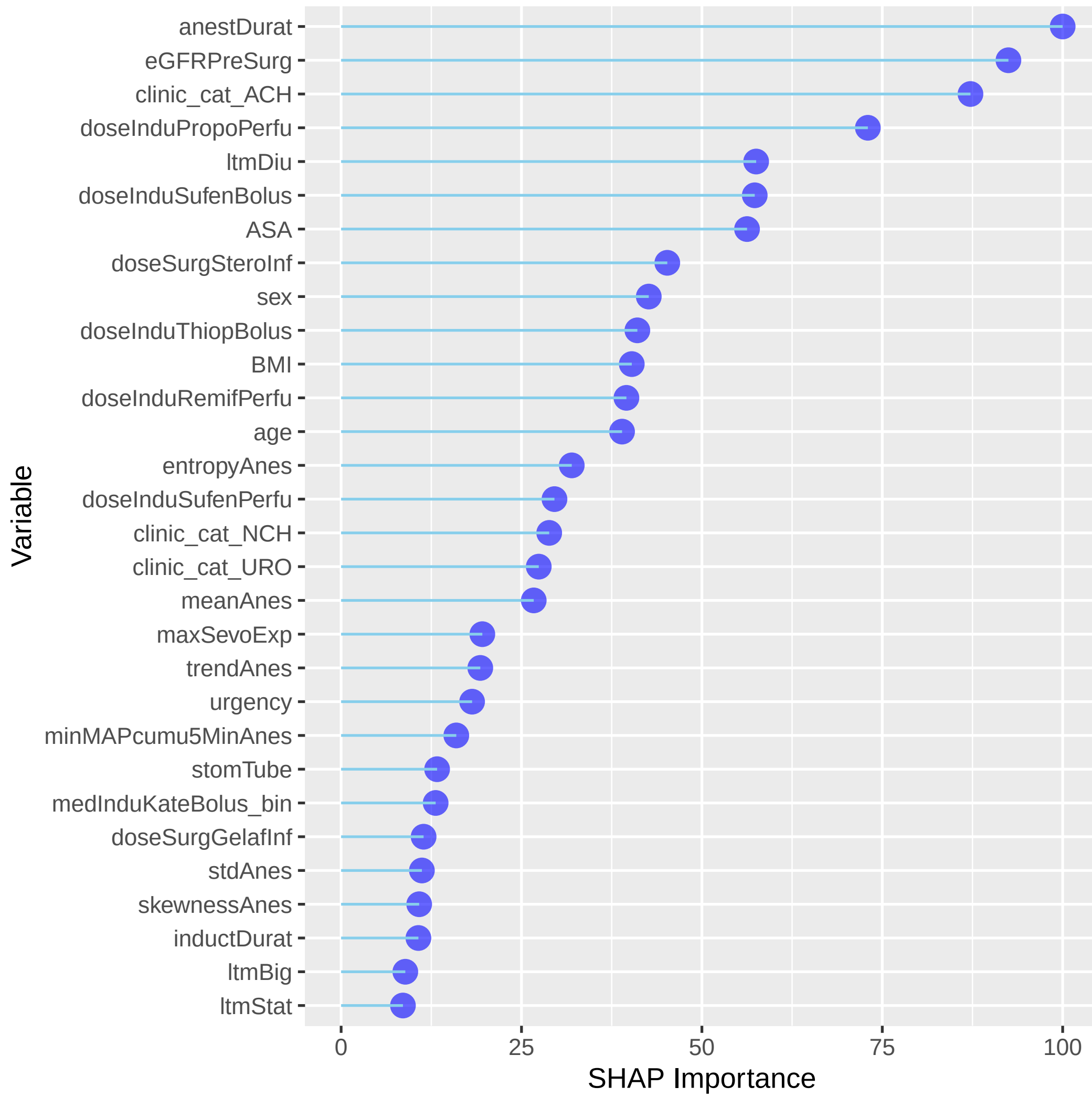

Figure 14. Scaled average Shapley values of the 30 most important predictors, assigned by a logistic Group-LASSO regression model trained on the complete balanced development cohort for the prediction of post-operative acute kidney injury after non-cardiac surgery. Shapley values were computed for all individual model predictions on the validation data set and then averaged to estimate overall feature contribution. All values are scaled to the Shapley value of the most important feature for better comparability.
Abbrev.: age, patient age; anestDurat, duration of anesthesia; ASA, American Society of Anesthesiology physical status; BMI, body mass index of patient; clinic_cat_ACH, general surgery; clinic_cat_NCH, neurology surgery; clinic_cat_URO, urology surgery; doseInduPropoPerfu, dose of propofol administered via perfusor during induction; doseInduRemifPerfu, dose of remifentanil administered via perfusor during induction; doseInduSufenBolus, dose of sufentanil administered via bolus during induction; doseInduSufenPerfu, dose of sufentanil administered via perfusor during induction; doseInduThiopBolus, dose of thiopental administered via bolus during induction; doseSurgGelafInf, dose of gelafundin administered via infusion during induction; doseSurgSteroInf, dose of sterofundin administered via infusion during induction; eGFRPreSurg, estimated glomerular filtration rate before surgery; entropyAnes, entropy of MAP during anesthesia; inductDurat, duration of induction of surgery; ltmBig, longterm medication with biguanides; ltmDiu, longterm medication with diuretics; ltmStat, longterm medication with statins; maxSevoExp, maximum expiratory sevoflurane measurement during induction; meanAnes, mean MAP during entire anesthesia; medInduKateBolus_bin, administration of catecholamine during induction; minMAPcumu5MinAnes, minimal MAP for 5 minutes during anesthesia; sex, biological sex of patient; skewnessAnes, skewness of MAP during anesthesia; stdAnes, standard deviation of MAP during anesthesia; stomTube, stomach tube; trendAnes, trend of MAP during anesthesia; urgency, level of urgency of surgical intervention.

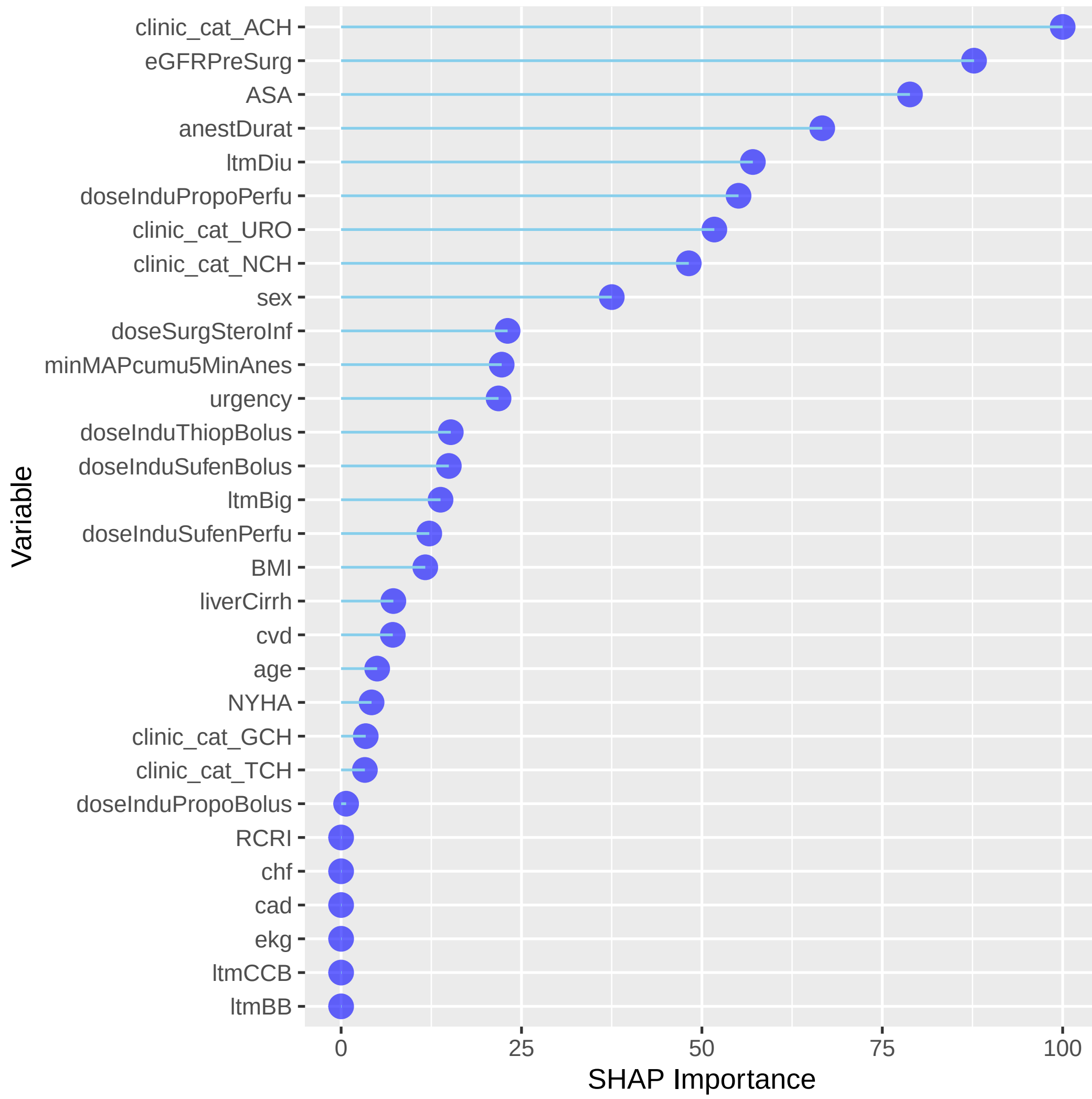

Figure 15. Scaled average Shapley values of the 30 most important predictors, assigned by a logistic LASSO regression model trained on the complete balanced development cohort for the prediction of post-operative acute kidney injury after non-cardiac surgery. Shapley values were computed for all individual model predictions on the validation data set and then averaged to estimate overall feature contribution. All values are scaled to the Shapley value of the most important feature for better comparability.

Abbrev.: age, patient age; anestDurat, duration of anesthesia; ASA, American Society of Anesthesiology physical status; BMI, body mass index of patient; cad, coronary heart disease; chf, chronic heart failure; clinic_cat_ACH, general surgery; clinic_cat_GCH, vascular surgery; clinic_cat_NCH, neurology surgery; clinic_cat_TCH, thoracic surgery; clinic_cat_URO, urology surgery; cvd, cerebrovascular disease; doseInduPropoBolus, dose of propofol administered via bolus during induction; doseInduPropoPerfu, dose of propofol administered via perfusor during induction; doseInduSufenBolus, dose of sufentanil administered via bolus during induction; doseInduSufenPerfu, dose of sufentanil administered via perfusor during induction; doseInduThiopPerfu, dose of thiopental administered via perfusor during induction; doseSurgSteroInf, dose of sterofundin administered via infusion during induction; eGFRPreSurg, estimated glomerular filtration rate before surgery; ekg, atrial fibrillation; liverCirrh, liver cirrhosis; ltmBB, longterm medication with beta-blockers; ltmBig, longterm medication with biguanides; ltmCCB, longterm medication with calcium channel blockers; ltmDiu, longterm medication with diuretics; minMAPcumu5MinAnes, minimal MAP for 5 minutes during anesthesia; NYHA, New York Heart Association classification of heart disease; RCRI, revised cardiac risk index; sex, biological sex of patient; urgency, level of urgency of surgical intervention.

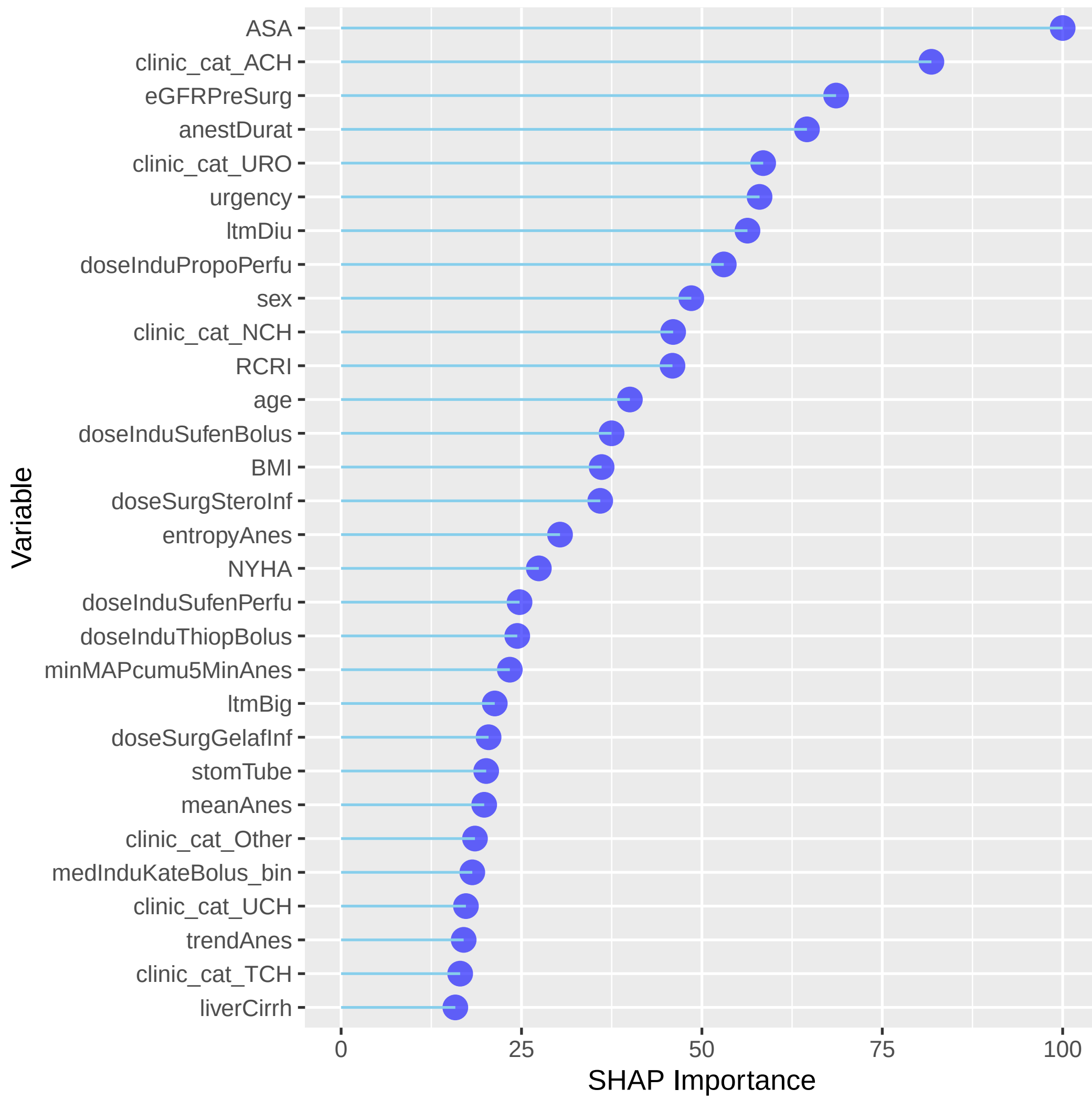

Figure 16. Scaled average Shapley values of the 30 most important predictors, assigned by a logistic Ridge regression model trained on the complete balanced development cohort for the prediction of post-operative acute kidney injury after non-cardiac surgery. Shapley values were computed for all individual model predictions on the validation data set and then averaged to estimate overall feature contribution. All values are scaled to the Shapley value of the most important feature for better comparability.
Abbrev.: age, patient age; anestDurat, duration of anesthesia; ASA, American Society of Anesthesiology physical status; BMI, body mass index of patient; clinic_cat_ACH, general surgery; clinic_cat_NCH, neurology surgery; clinic_cat_TCH, thoracic surgery; clinic_cat_UCH, trauma surgery; clinic_cat_URO, urology surgery; clinic_cat_Other, other surgery department; doseInduPropoPerfu, dose of propofol administered via perfusor during induction; doseInduSufenBolus, dose of sufentanil administered via bolus during induction; doseInduSufenPerfu, dose of sufentanil administered via perfusor during induction; doseInduThiopBolus, dose of thiopental administered via bolus during induction; doseSurgGelafInf, dose of gelafundin administered via infusion during induction; doseSurgSterofInf, dose of sterofundin administered via infusion during induction; eGFRPreSurg, estimated glomerular filtration rate before surgery; entropyAnes, entropy of MAP during anesthesia; liverCirrh, liver cirrhosis; ltmBig, longterm medication with biguanides; ltmDiu, longterm medication with diuretics; meanAnes, mean MAP during entire anesthesia; medSurgKateBolus_bin, administration of catecholamine during surgery; minMAPcumu5MinAnes, minimal MAP for 5 minutes during anesthesia; NYHA, New York Heart Association classification of heart disease; RCRI, revised cardiac risk index; sex, biological sex of patient; stomTube, stomach tube; trendAnes, trend of MAP during anesthesia; urgency, level of urgency of surgical intervention.

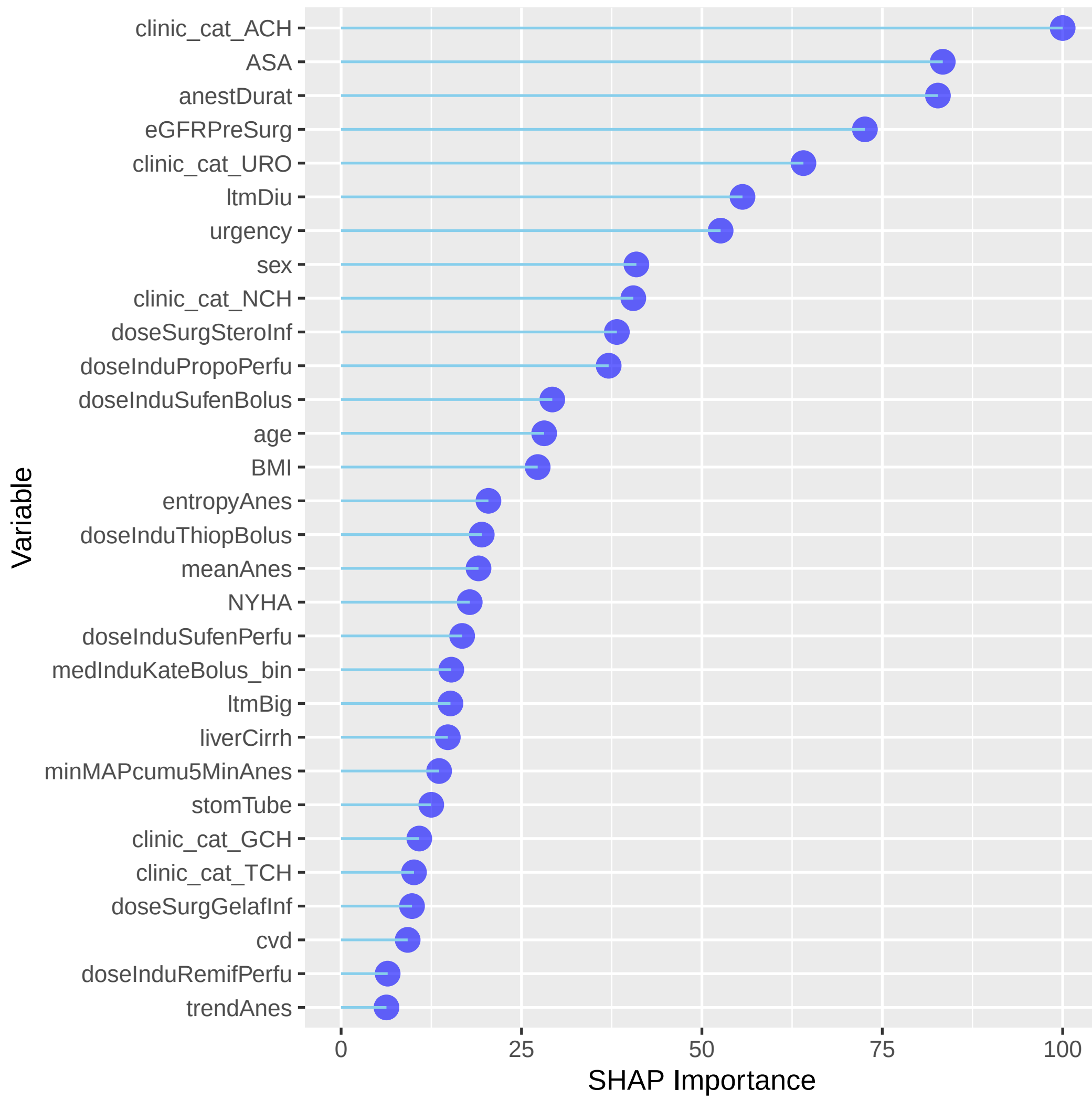

Figure 17. Scaled average Shapley values of the 30 most important predictors, assigned by a logistic Elastic Net regression model trained on the complete balanced development cohort for the prediction of post-operative acute kidney injury after non-cardiac surgery. Shapley values were computed for all individual model predictions on the validation data set and then averaged to estimate overall feature contribution. All values are scaled to the Shapley value of the most important feature for better comparability.

Abbrev.: age, patient age; anestDurat, duration of anesthesia; ASA, American Society of Anesthesiology physical status; BMI, body mass index of patient; clinic_cat_ACH, general surgery; clinic_cat_GCH, gynaecology surgery; clinic_cat_NCH, neurology surgery; clinic_cat_TCH, thoracic surgery; clinic_cat_URO, urology surgery; cvd, cerebrovascular disease; doseInduPropoPerfu, dose of propofol administered via perfusor during induction; doseInduRemifPerfu, dose of remifentanil administered via perfusor during induction; doseInduSufenBolus, dose of sufentanil administered via bolus during induction; doseInduSufenPerfu, dose of sufentanil administered via perfusor during induction; doseInduThiopBolus, dose of thiopental administered via bolus during induction; doseSurgGelafInf, gose of gelafundin administered via infusion during induction; doseSurgSteroInf, gose of sterofundin administered via infusion during induction; eGFRPreSurg, estimated glomerular filtration rate before surgery; entropyAnes, entropy of MAP during anesthesia; liverCirrh, liver cirrhosis; ltmBig, longterm medication with biguanides; ltmDiu, longterm medication with diuretics; meanAnes, mean MAP during entire anesthesia; medInduKateBolus_bin, administration of catecholamine during induction; minMAPcumu5MinAnes, minimal MAP for 5 minutes during anesthesia; NYHA, New York Heart Association classification of heart disease; sex, biological sex of patient; stomTube, stomach tube; trendAnes, trend of MAP during anesthesia; urgency, level of urgency of surgical intervention.

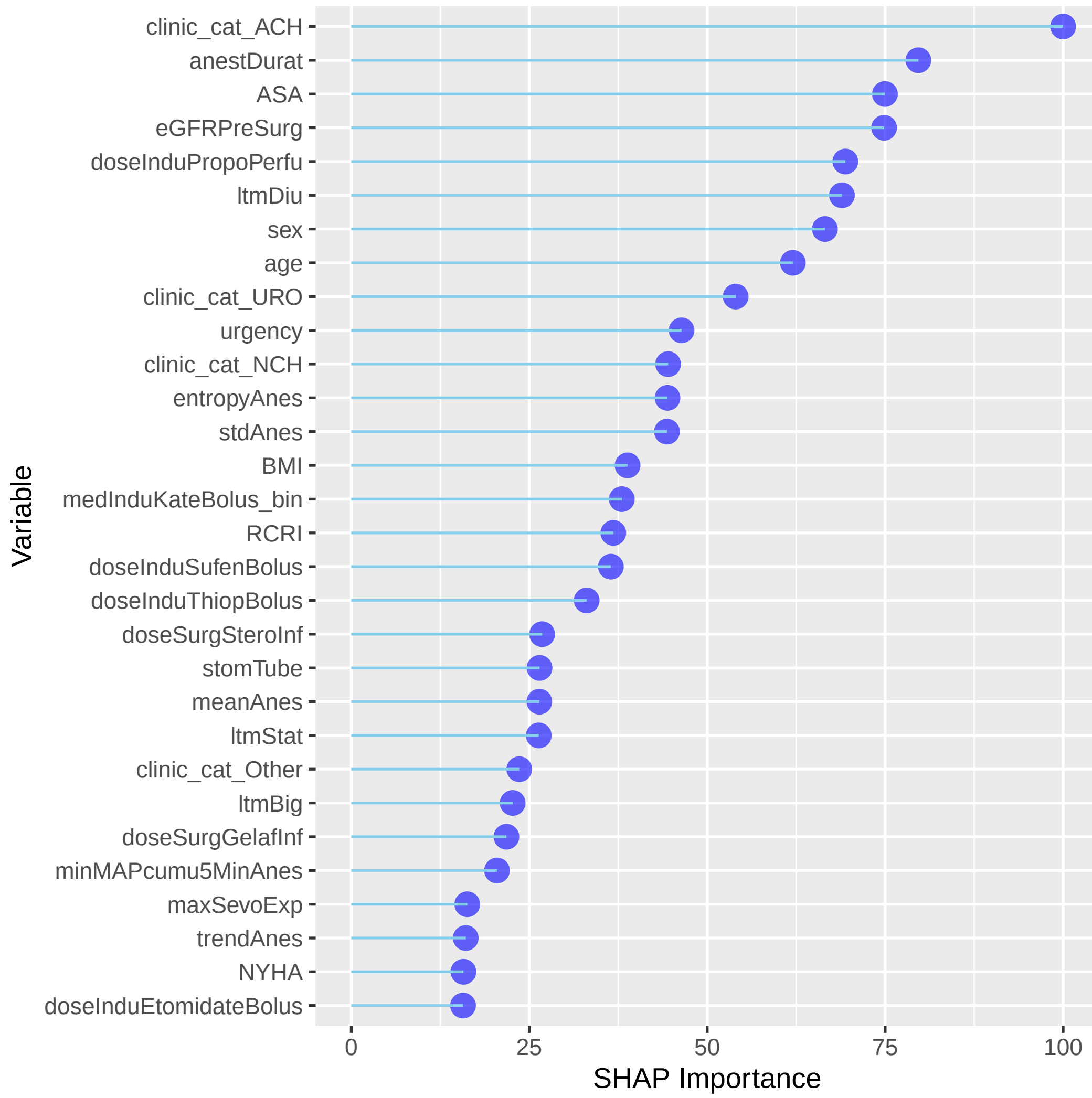

Figure 18. **Scaled average Shapley values of the 30 most important predictors, assigned by a SVM model trained on the complete balanced development cohort for the prediction of post-operative acute kidney injury after non-cardiac surgery.** Shapley values were computed for all individual model predictions on the validation data set and then averaged to estimate overall feature contribution. All values are scaled to the Shapley value of the most important feature for better comparability.

Abbrev.: age, patient age; anestDurat, duration of anesthesia; ASA, American Society of Anesthesiology physical status; BMI, body mass index of patient; clinic_cat_ACH, general surgery; clinic_cat_NCH, neurology surgery; clinic_cat_Other, other type of surgery; clinic_cat_URO, urology surgery; doseInduEtomidateBolus, dose of etomidate administered via bolus during induction; doseInduPropoPerfu, dose of propofol administered via perfusor during induction; doseInduSufenBolus, dose of sufentanil administered via bolus during induction; doseInduThiopBolus, dose of thiopental administered via bolus during induction; doseSurgGelafInf, dose of gelafundin administered via infusion during induction; doseSurgSteroInf, dose of sterofundin administered via infusion during induction; eGFRPreSurg, estimated glomerular filtration rate before surgery; entropyAnes, entropy of MAP during anesthesia; ltmBig, longterm medication with biguanides; ltmDiu, longterm medication with diuretics; ltmStat, longterm medication with statins; maxSevoExp, maximum expiratory sevoflurane measurement during induction; meanAnes, mean MAP during entire anesthesia; medInduKateBolus_bin, administration of catecholamine during induction; minMAPcumu5MinAnes, minimal MAP for 5 minutes during anesthesia; NYHA, New York Heart Association classification of heart disease; RCRI, revised cardiac risk index; sex, biological sex of patient; stdAnes, standard deviation of MAP during anesthesia; stomTube, stomach tube; trendAnes, trend of MAP during anesthesia; urgency, level of urgency of surgical intervention.

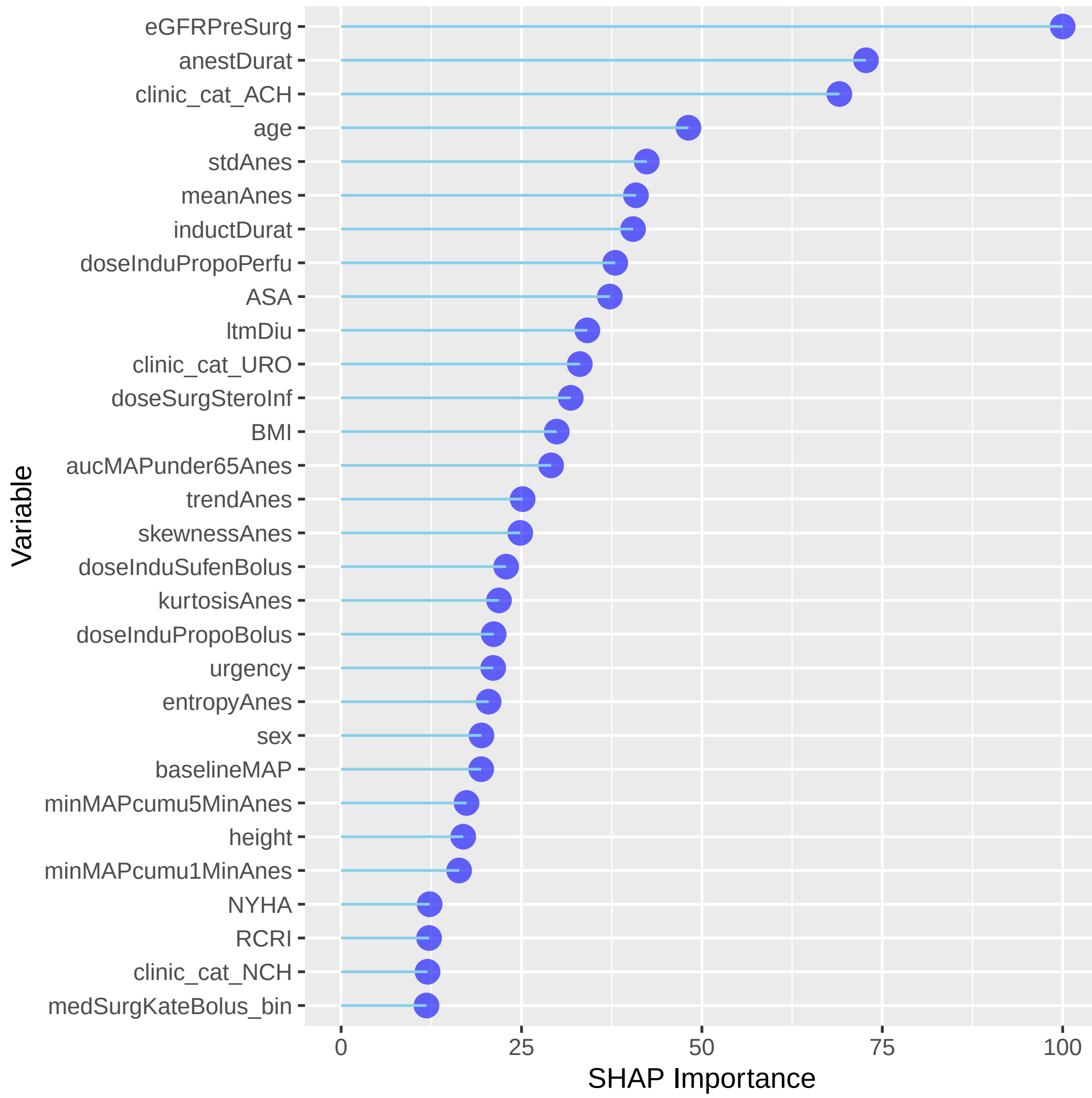

Figure 19. Scaled average Shapley values of the 30 most important predictors, assigned by a linear xGBM model trained on the complete balanced development cohort for the prediction of post-operative acute kidney injury after non-cardiac surgery. Shapley values were computed for all individual model predictions on the validation data set and then averaged to estimate overall feature contribution. All values are scaled to the Shapley value of the most important feature for better comparability.

Abbrev.: age, patient age; anestDurat, duration of anesthesia; ASA, American Society of Anesthesiology physical status; aucMAPunder65Anes, area under the curve for all MAP below 65mmHg during anesthesia; baselineMAP, first measured MAP value; BMI, body mass index of patient; clinic_cat_ACH, general surgery; clinic_cat_NCH, neurology surgery; clinic_cat_URO, urology surgery; doseInduPropoBolus, dose of propofol administered via bolus during induction; doseInduPropoPerfu, dose of propofol administered via perfusor during induction; doseInduSufenBolus, dose of sufentanil administered via bolus during induction; doseSurgSteroInf, dose of sterofundin administered via infusion during induction; eGFRPreSurg, estimated glomerular filtration rate before surgery; entropyAnes, entropy of MAP during anesthesia; height, patient height; inductDurat, duration of induction of surgery; kurtosisAnes, kurtosis of MAP during anesthesia; ltmDiu, longterm medication with diuretics; meanAnes, mean MAP during entire anesthesia; medSurgKateBolus_bin, administration of catecholamine during surgery; minMAPcumu1MinAnes, minimal MAP for 1 minute during anesthesia; minMAPcumu5MinAnes, minimal MAP for 5 minutes during anesthesia; NYHA, New York Heart Association classification of heart disease; RCRT, revised cardiac risk index; sex, biological sex of patient; skewnessAnes, skewness of MAP during anesthesia; stdAnes, standard deviation of MAP during anesthesia; trendAnes, trend of MAP during anesthesia; urgency, level of urgency of surgical intervention.

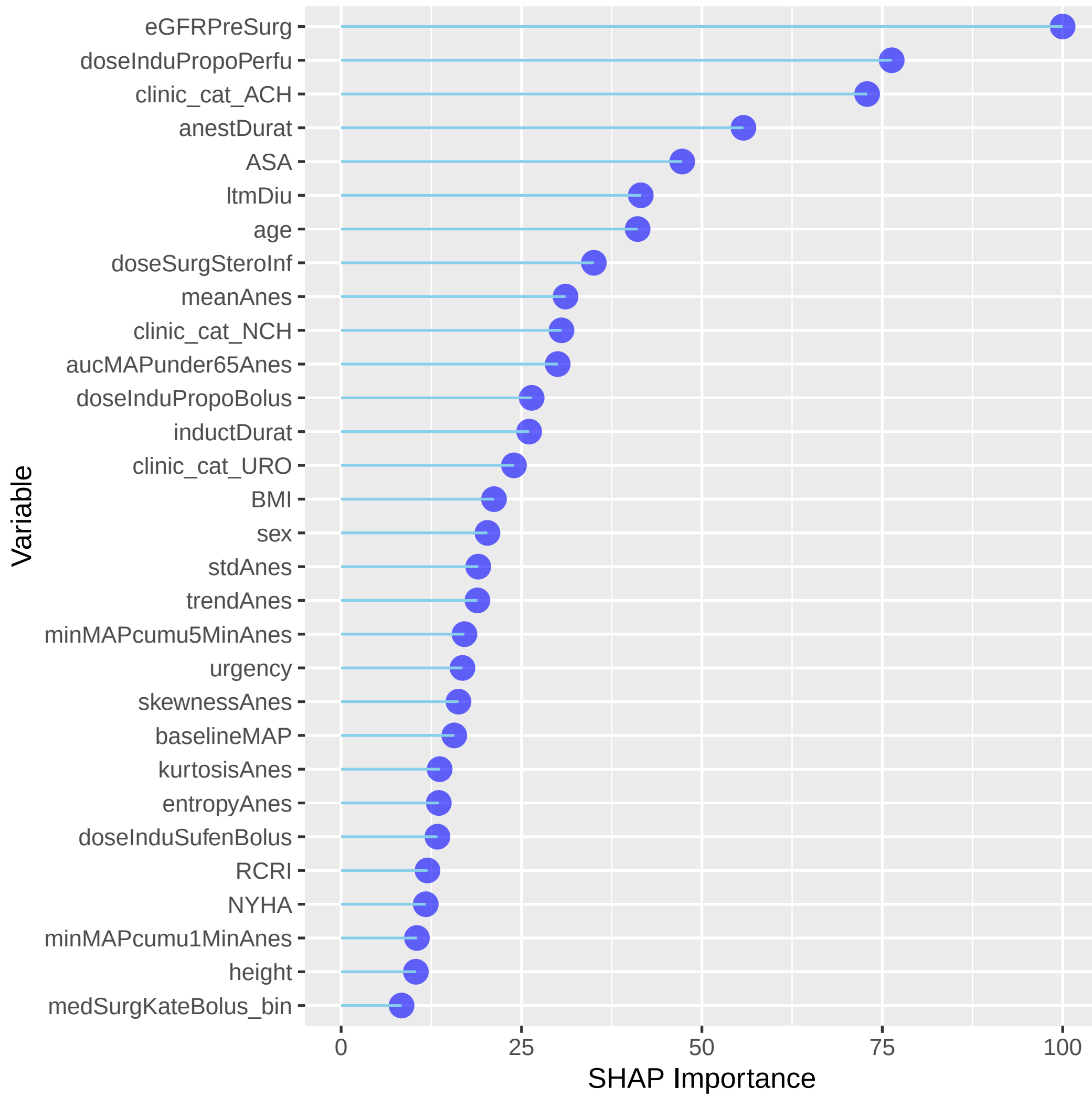

Figure 20. **Scaled average Shapley values of the 30 most important predictors, assigned by a tree-based xGBM model trained on the complete balanced development cohort for the prediction of post-operative acute kidney injury after non-cardiac surgery.** Shapley values were computed for all individual model predictions on the validation data set and then averaged to estimate overall feature contribution. All values are scaled to the Shapley value of the most important feature for better comparability.
Abbrev.: age, patient age; anestDurat, duration of anesthesia; ASA, American Society of Anesthesiology physical status; aucMAPunder65Anes, area under the curve for all MAP below 65mmHg during anesthesia; baselineMAP, first measured MAP value; BMI, body mass index of patient; clinic_cat_ACH, general surgery; clinic_cat_NCH, neurology surgery; clinic_cat_URO, urology surgery; doseInduPropoBolus, dose of propofol administered via bolus during induction; doseInduPropoPerfu, dose of propofol administered via perfusor during induction; doseInduSufenBolus, dose of sufentanil administered via bolus during induction; doseSurgSteroInf, dose of sterofundin administered via infusion during induction; eGFRPreSurg, estimated glomerular filtration rate before surgery; entropyAnes, entropy of MAP during anesthesia; height, patient height; inductDurat, duration of induction of surgery; kurtosisAnes, kurtosis of MAP during anesthesia; ltmDiu, longterm medication with diuretics; meanAnes, mean MAP during entire anesthesia; medSurgKateBolus_bin, administration of catecholamine during surgery; minMAPcumu1MinAnes, minimal MAP for 1 minute during anesthesia; minMAPcumu5MinAnes, minimal MAP for 5 minutes during anesthesia; NYHA, New York Heart Association classification of heart disease; RCRI, revised cardiac risk index; sex, biological sex of patient; skewnessAnes, skewness of MAP during anesthesia; stdAnes, standard deviation of MAP during anesthesia; trendAnes, trend of MAP during anesthesia; urgency, level of urgency of surgical intervention.

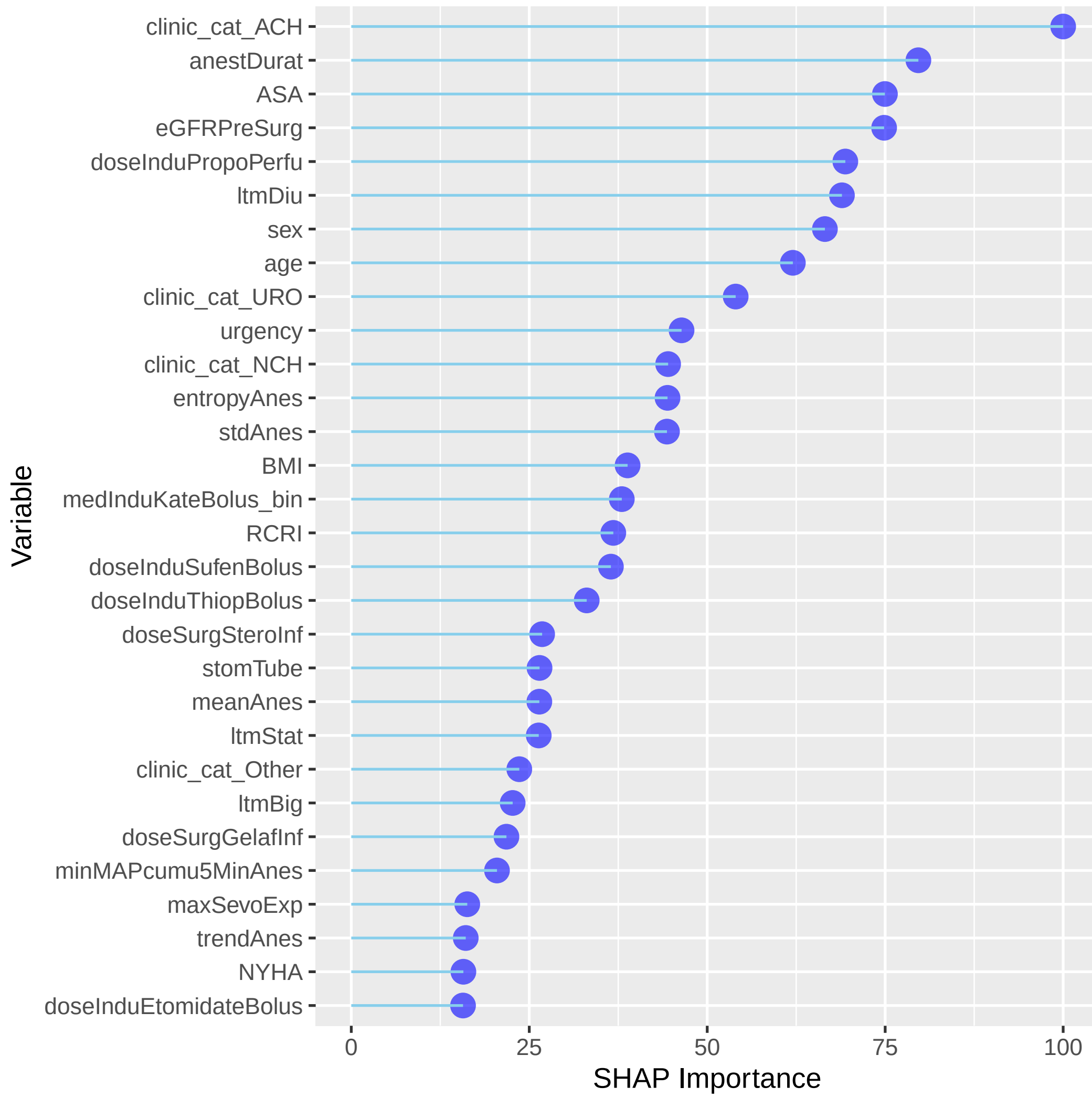

Figure 21. Scaled average Shapley values of the 30 most important predictors, assigned by a Random Forest model trained on the complete balanced development cohort for the prediction of post-operative acute kidney injury after non-cardiac surgery. Shapley values were computed for all individual model predictions on the validation data set and then averaged to estimate overall feature contribution. All values are scaled to the Shapley value of the most important feature for better comparability.

Abbrev.: age, patient age; anestDurat, duration of anesthesia; ASA, American Society of Anesthesiology physical status; BMI, body mass index of patient; clinic_cat_ACH, general surgery; clinic_cat_NCH, neurology surgery; clinic_cat_Other, other surgery department; clinic_cat_URO, urology surgery; doseInduEtomidateBolus, dose of etomidate administered via bolus during induction; doseInduPropoPerfu, dose of propofol administered via perfusor during induction; doseInduSufenBolus, dose of sufentanil administered via bolus during induction; doseInduThiopBolus, dose of thiopental administered via bolus during induction; doseSurgGelafInf, dose of gelafundin administered via infusion during induction; doseSurgSteroInf, dose of sterofundin administered via infusion during induction; eGFRPreSurg, estimated glomerular filtration rate before surgery; entropyAnes, entropy of MAP during anesthesia; ltmBig, longterm medication with biguanides; ltmDiu, longterm medication with diuretics; ltmStat, longterm medication with statins; maxSevoExp, maximum expiratory sevoflurane measurement during induction; meanAnes, mean MAP during entire anesthesia; medInduKateBolus_bin, administration of catecholamine during induction; minMAPcumu5MinAnes, minimal MAP for 5 minutes during anesthesia; NYHA, New York Heart Association classification of heart disease; RCRI, revised cardiac risk index; sex, biological sex of patient; stdAnes, standard deviation of MAP during anesthesia; stomTube, stomach tube; trendAnes, trend of MAP during anesthesia; urgency, level of urgency of surgical intervention.

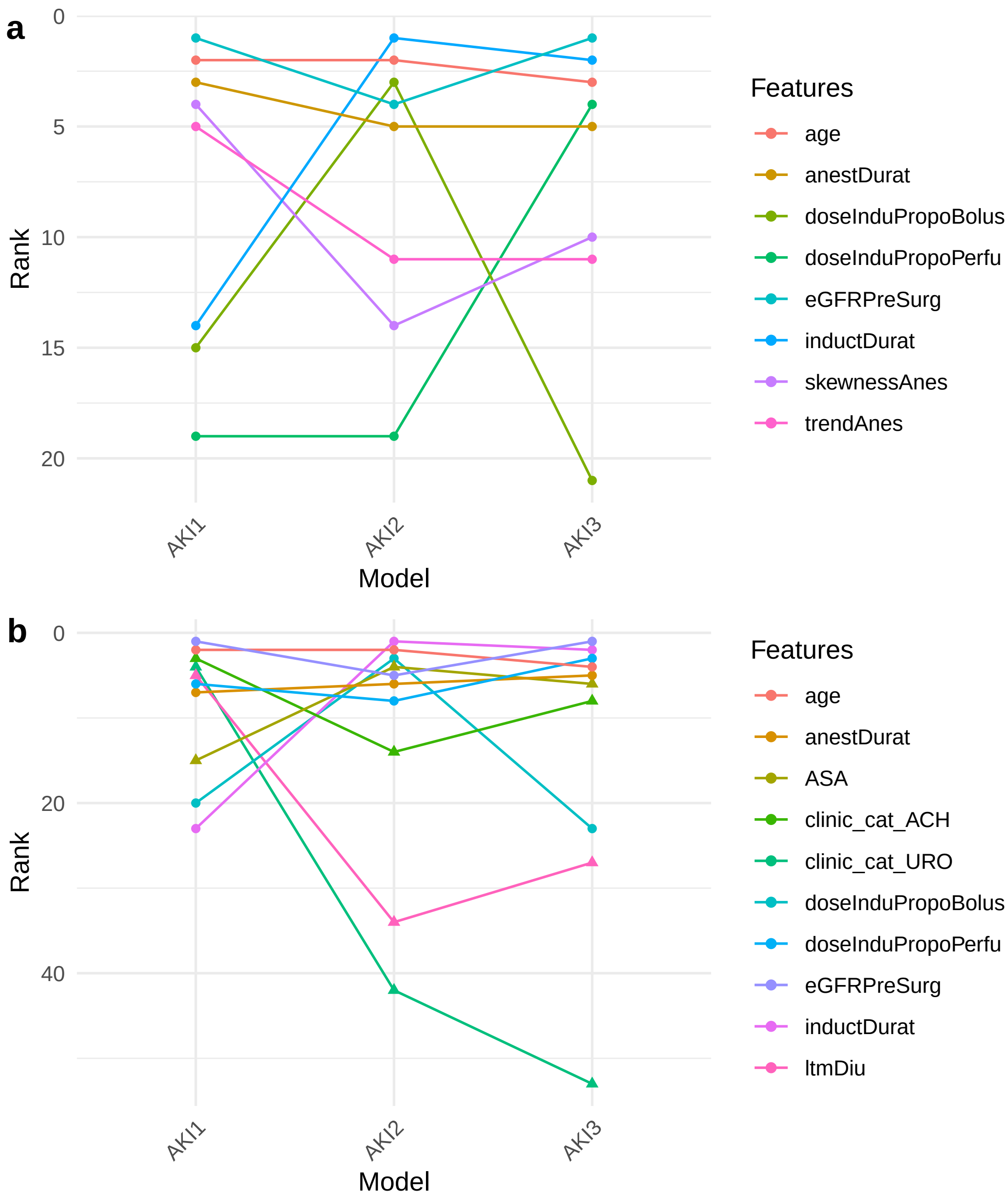

Figure 22. Ranks of native (a) and post-hoc assessed (b) Feature Importance (FI) assigned by PO-AKI prediction models trained with different PO-AKI stages as outcome variable (x-axis). The FI ranks across all models (y-axis) are displayed for the most important features (ranks 1-5). Predictors were ranked based on the absolute native FI that was estimated by the respective model (a) and the largest average Shapley Additive Explanations Value assigned to a feature within 100 bootstrap replicates (b). Lower rank values indicate higher importance with rank 1 representing highest importance. Continuous features are shown as dots, categorical features are displayed as triangles, respectively.
Abbrev.: age, patient age; anestDurat, duration of anesthesia; ASA, American Society of Anesthesiology physical status; clinic_cat_ACH, general surgery; clinic_cat_URO, urology surgery; doseInduPropoBolus, dose of propofol administered via bolus during induction; doseInduPropoPerfu, dose of propofol administered via perfusor during induction; eGFRPreSurg, estimated glomerular filtration rate before surgery; inductDurat, duration of induction of surgery; ltmDiu, longterm medication with diuretics; skewnessAnes, skewness of MAP during anesthesia; trendAnes, trend of MAP during anesthesia.

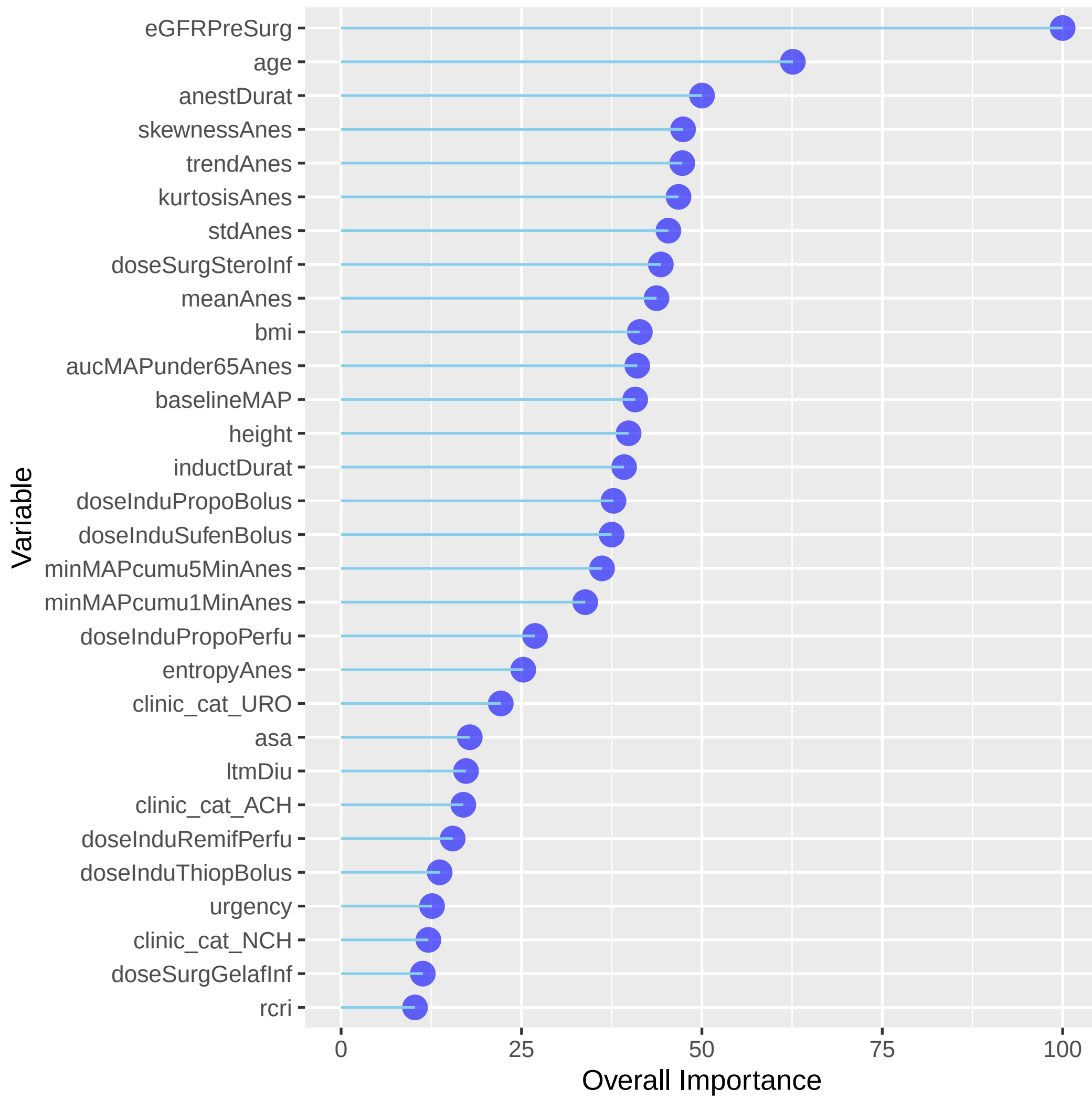

Figure 23. Scaled native Feature Importances (FIs) of the 30 most important predictors, assigned by a Random Forest Classifier (RFC), trained on a balanced development cohort for the prediction of post-operative acute kidney injury stage 1 after non-cardiac surgery. FI measures are based on the averaged contribution of each feature to the total gain of this feature's splits. All weights are scaled to the highest FI, which is assigned a value of 100, for better comparability. Abbrev.: asa, American Society of Anesthesiology physical status; age, patient age; anestDurat, duration of anesthesia; aucMAPunder65Anes, area under the curve for all MAP below 65mmHg during anesthesia; baselineMAP, first measured MAP value; BMI, Body Mass Index of patient; clinic_cat_ACH, general surgery; clinic_cat_NCH, neurology surgery; clinic_cat_URO, urology surgery; doseInduPropoBolus, dose of propofol administered via bolus during induction; doseInduPropoPerfu, dose of propofol administered via perfusor during induction; doseInduRemifPerfu, dose of remifentanil administered via perfusor during induction; doseInduSufenBolus, dose of sufentanil administered via bolus during induction; doseInduThiopBolus, dose of thiopental administered via bolus during induction; doseSurgGelafInf, dose of gelafundin administered via infusion during induction; doseSurgSteroInf, dose of sterofundin administered via infusion during induction; eGFRPreSurg, estimated glomerular filtration rate before surgery; entropyAnes, entropy of MAP during anesthesia; height, patient height; inductDurat, duration of induction of surgery; kurtosisAnes, kurtosis of MAP during anesthesia; ltmDiu, longterm medication with diuretics; meanAnes, mean MAP during entire anesthesia; minMAPcumu1MinAnes, minimal MAP for 1 minute during anesthesia; minMAPcumu5MinAnes, minimal MAP for 5 minutes during anesthesia; rcri, revised cardiac risk index; skewnessAnes, skewness of MAP during anesthesia; stdAnes, standard deviation of MAP during anesthesia; trendAnes, trend of MAP during anesthesia; urgency, level of urgency of surgical intervention.

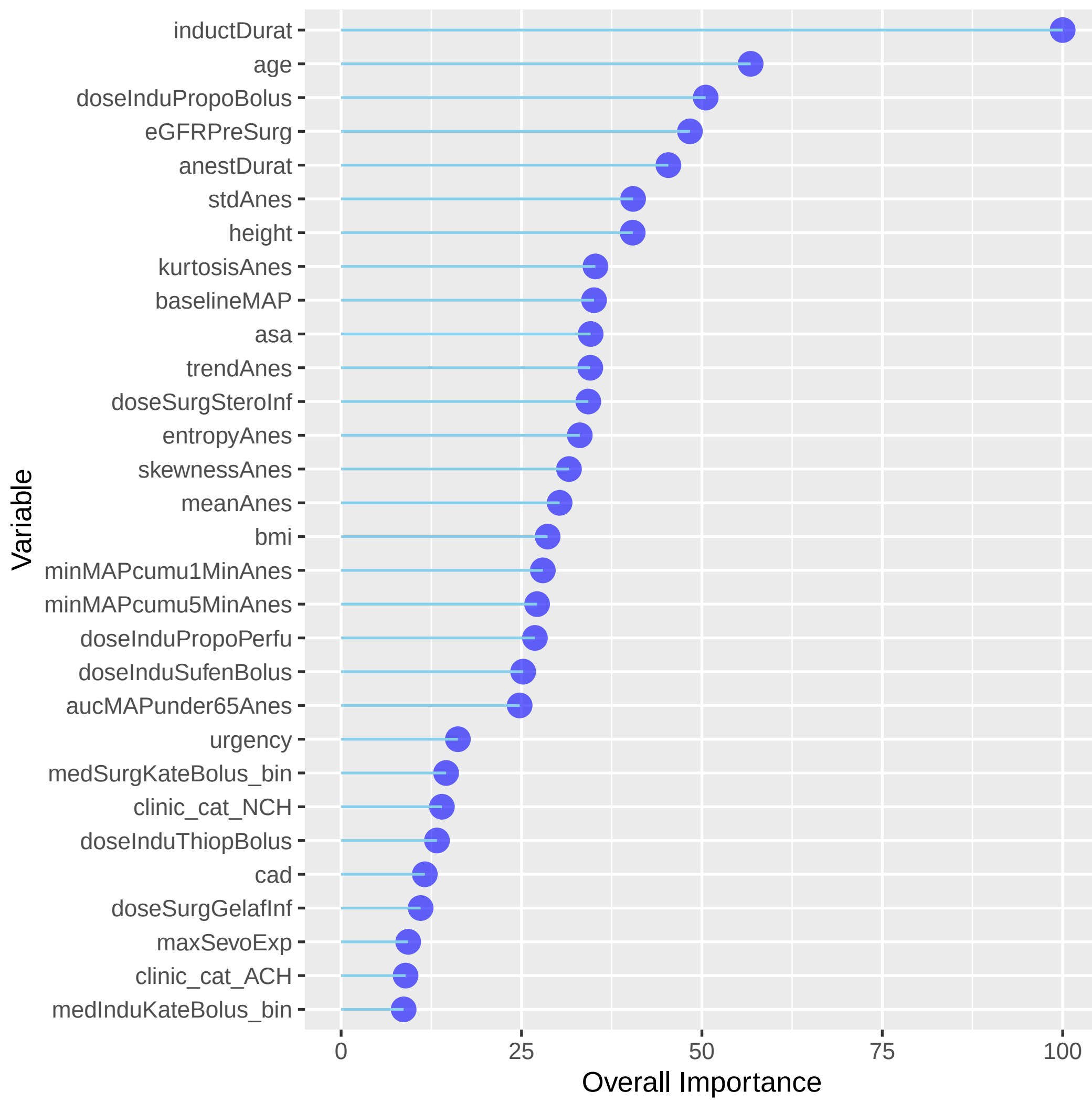

Figure 24. Scaled native Feature Importances (FIs) of the 30 most important predictors, assigned by a Random Forest Classifier (RFC), trained on a balanced development cohort for the prediction of post-operative acute kidney injury stage 2 after non-cardiac surgery. FI measures are based on the averaged contribution of each feature to the total gain of this feature's splits. All weights are scaled to the highest FI, which is assigned a value of 100, for better comparability. Abbrev.: asa, American Society of Anesthesiology physical status; age, patient age; anestDurat, duration of anesthesia; aucMAPunder65Anes, area under the curve for all MAP below 65mmHg during anesthesia; baselineMAP, first measured MAP value; bmi, Body Mass Index of patient; clinic_cat_ACH, general surgery; clinic_cat_NCH, neurology surgery; cad, coronary heart disease; doseInduPropoBolus, dose of propofol administered via bolus during induction; doseInduPropoPerfu, dose of propofol administered via perfusor during induction; doseInduThiopBolus, dose of thiopental administered via bolus during induction; doseSurgGelafInf, dose of gelafundin administered via infusion during induction; doseSurgSteroInf, dose of sterofundin administered via infusion during induction; eGFRPreSurg, estimated glomerular filtration rate before surgery; entropyAnes, entropy of MAP during anesthesia; height, patient height; inductDurat, duration of induction of surgery; kurtosisAnes, kurtosis of MAP during anesthesia; maxSevoExp, maximum expiratory sevoflurane measurement during induction; meanAnes, mean MAP during entire anesthesia; medSurgKateBolus_bin, administration of catecholamine during surgery; minMAPcumu1MinAnes, minimal MAP for 1 minute during anesthesia; minMAPcumu5MinAnes, minimal MAP for 5 minutes during anesthesia; skewnessAnes, skewness of MAP during anesthesia; stdAnes, standard deviation of MAP during anesthesia; trendAnes, trend of MAP during anesthesia; urgency, level of urgency of surgical intervention.

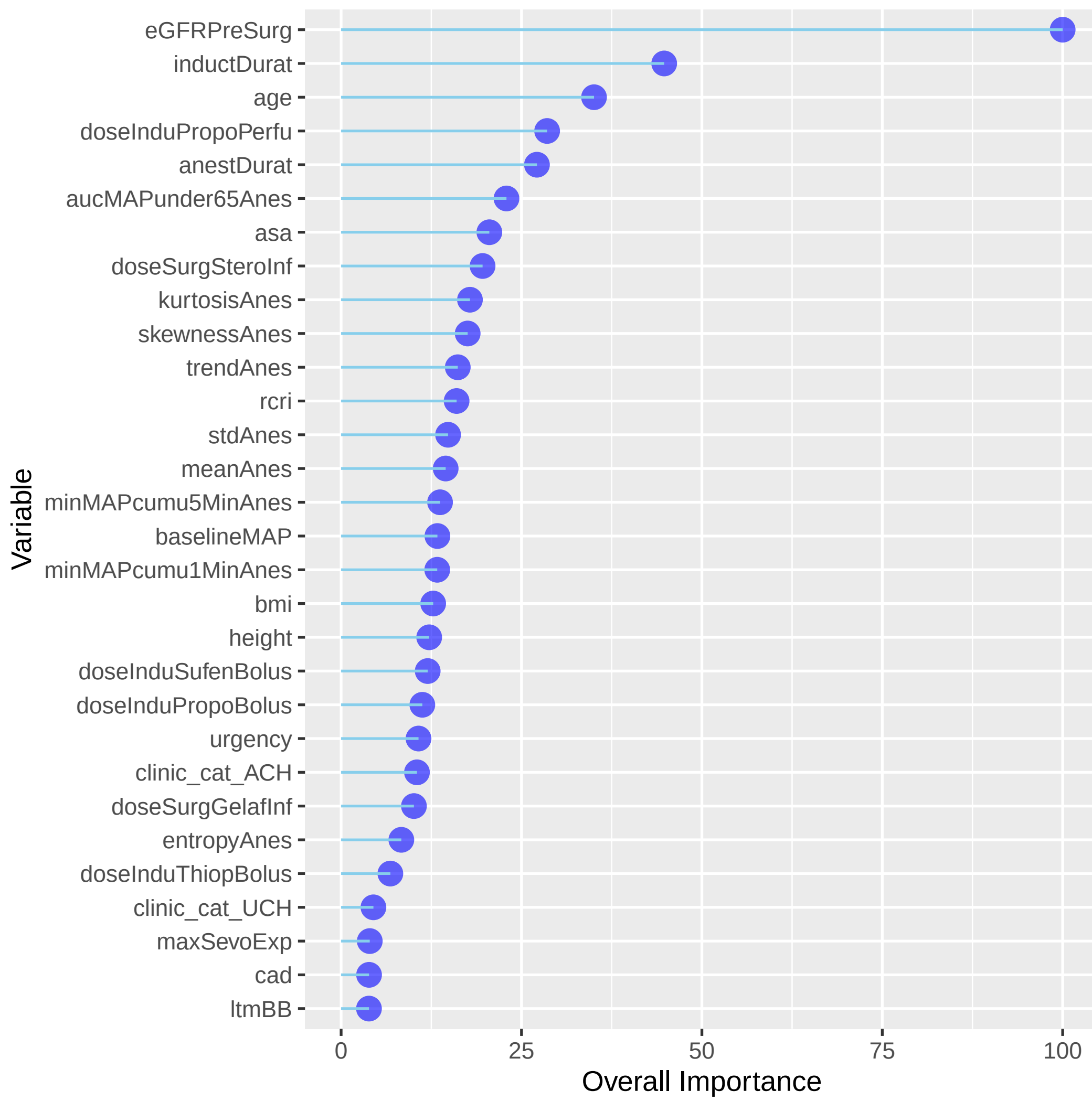

Figure 25. **Scaled native Feature Importances (FIs) of the 30 most important predictors, assigned by a Random Forest Classifier (RFC), trained on a balanced development cohort for the prediction of post-operative acute kidney injury stage 3 after non-cardiac surgery.** FI measures are based on the averaged contribution of each feature to the total gain of this feature's splits. All weights are scaled to the highest FI, which is assigned a value of 100, for better comparability. Abbrev.: asa, American Society of Anesthesiology physical status; age, patient age; anestDurat, duration of anesthesia; aucMAPunder65Anes, area under the curve for all MAP below 65mmHg during anesthesia; baselineMAP, first measured MAP value; BMI, Body Mass Index of patient; clinic_cat_ACH, general surgery; clinic_cat_NCH, neurology surgery; clinic_cat_URO, urology surgery; cad, coronary heart disease; doseInduPropoBolus, dose of propofol administered via bolus during induction; doseInduPropoPerfu, dose of propofol administered via perfusor during induction; doseInduRemifPerfu, dose of remifentanil administered via perfusor during induction; doseInduSufenBolus, dose of sufentanil administered via bolus during induction; doseInduThiopBolus, dose of thiopental administered via bolus during induction; doseSurgGelafInf, dose of gelafundin administered via infusion during induction; doseSurgSteroInf, dose of sterofundin administered via infusion during induction; eGFRPreSurg, estimated glomerular filtration rate before surgery; entropyAnes, entropy of MAP during anesthesia; height, patient height; inductDurat, duration of induction of surgery; kurtosisAnes, kurtosis of MAP during anesthesia; ltmBB, longterm medication with beta-blockers; maxSevoExp, maximum expiratory sevoflurane measurement during induction; meanAnes, mean MAP during entire anesthesia; medSurgKateBolus_bin, administration of catecholamine during surgery; minMAPcumu1MinAnes, minimal MAP for 1 minute during anesthesia; minMAPcumu5MinAnes, minimal MAP for 5 minutes during anesthesia; rcri, revised cardiac risk index; skewnessAnes, skewness of MAP during anesthesia; stdAnes, standard deviation of MAP during anesthesia; trendAnes, trend of MAP during anesthesia; urgency, level of urgency of surgical intervention.

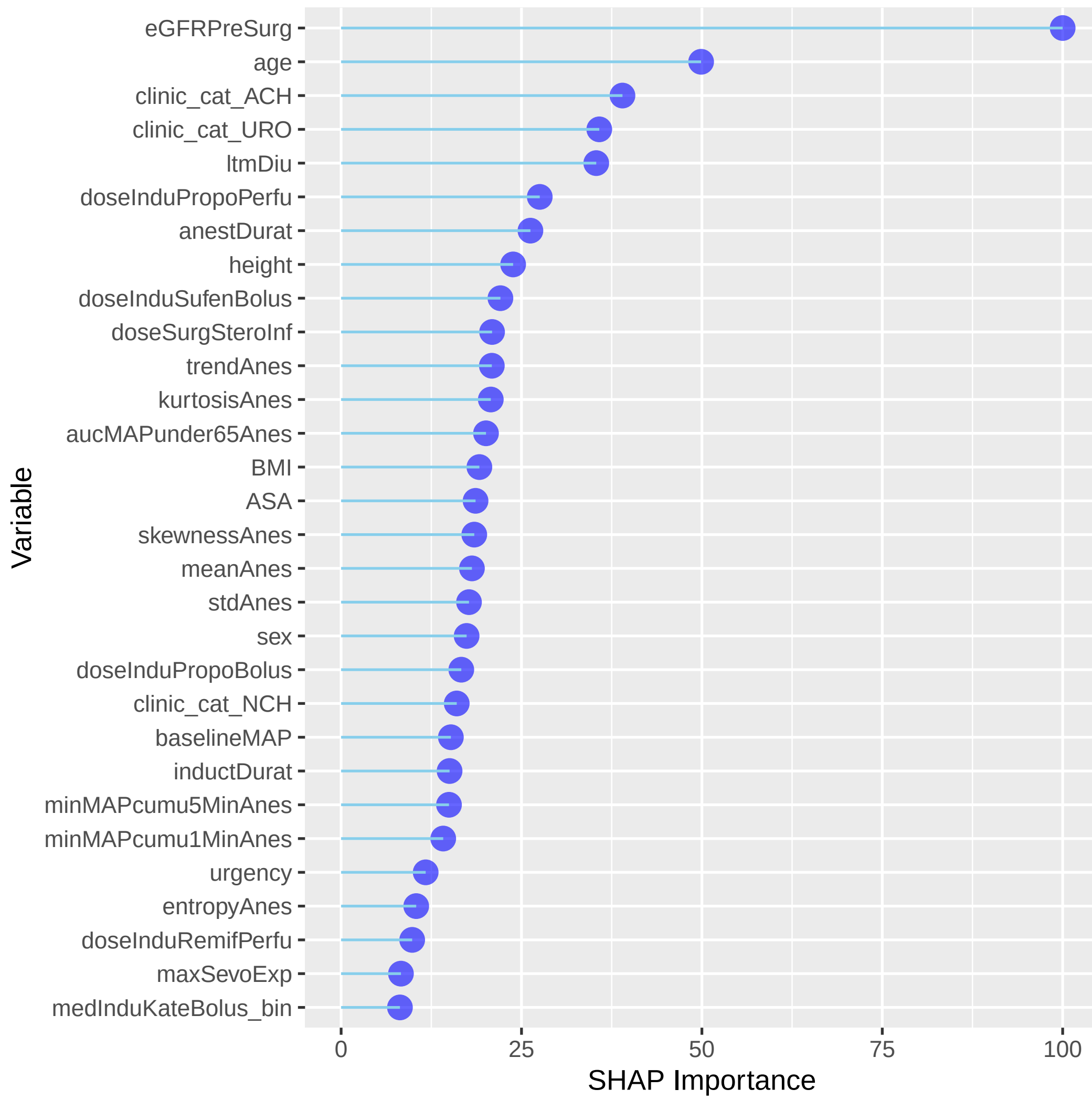

Figure 26. Scaled average Shapley values of the 30 most important predictors, assigned by a Random Forest Classifier (RFC), trained on a balanced development cohort for the prediction of post-operative acute kidney injury stage 1 after non-cardiac surgery. Shapley values were computed for all individual model predictions on the validation data set and then averaged to estimate overall feature contribution. All values are scaled to the Shapley value of the most important feature for better comparability.

Abbrev.: age, patient age; anestDurat, duration of anesthesia; ASA, American Society of Anesthesiology physical status; aucMAPunder65Anes, area under the curve for all MAP below 65mmHg during anesthesia; baselineMAP, first measured MAP value; BMI, body mass index of patient; clinic_cat_ACH, general surgery; clinic_cat_NCH, neurology surgery; doseInduPropoBolus, dose of propofol administered via bolus during induction; doseInduPropoPerfu, dose of propofol administered via perfusor during induction; doseInduSufenBolus, dose of sufentanil administered via bolus during induction; doseInduRemifPerfu, dose of remifentanil administered via perfusor during induction; eGFRPreSurg, estimated glomerular filtration rate before surgery; entropyAnes, entropy of MAP during anesthesia; height, patient height; inductDurat, duration of induction of surgery; kurtosisAnes, kurtosis of MAP during anesthesia; ltmDiu, longterm medication with diuretics; maxSevoExp, maximum expiratory sevoflurane measurement during induction; meanAnes, mean MAP during entire anesthesia; medInduKateBolus_bin, administration of catecholamine during induction; minMAPcumu5MinAnes, minimal MAP for 5 minutes during anesthesia; NYHA, New York Heart Association classification of heart disease; RCRI, revised cardiac risk index; sex, biological sex of patient; stdAnes, standard deviation of MAP during anesthesia; skewnessAnes, skewness of MAP during anesthesia; trendAnes, trend of MAP during anesthesia; urgency, level of urgency of surgical intervention.

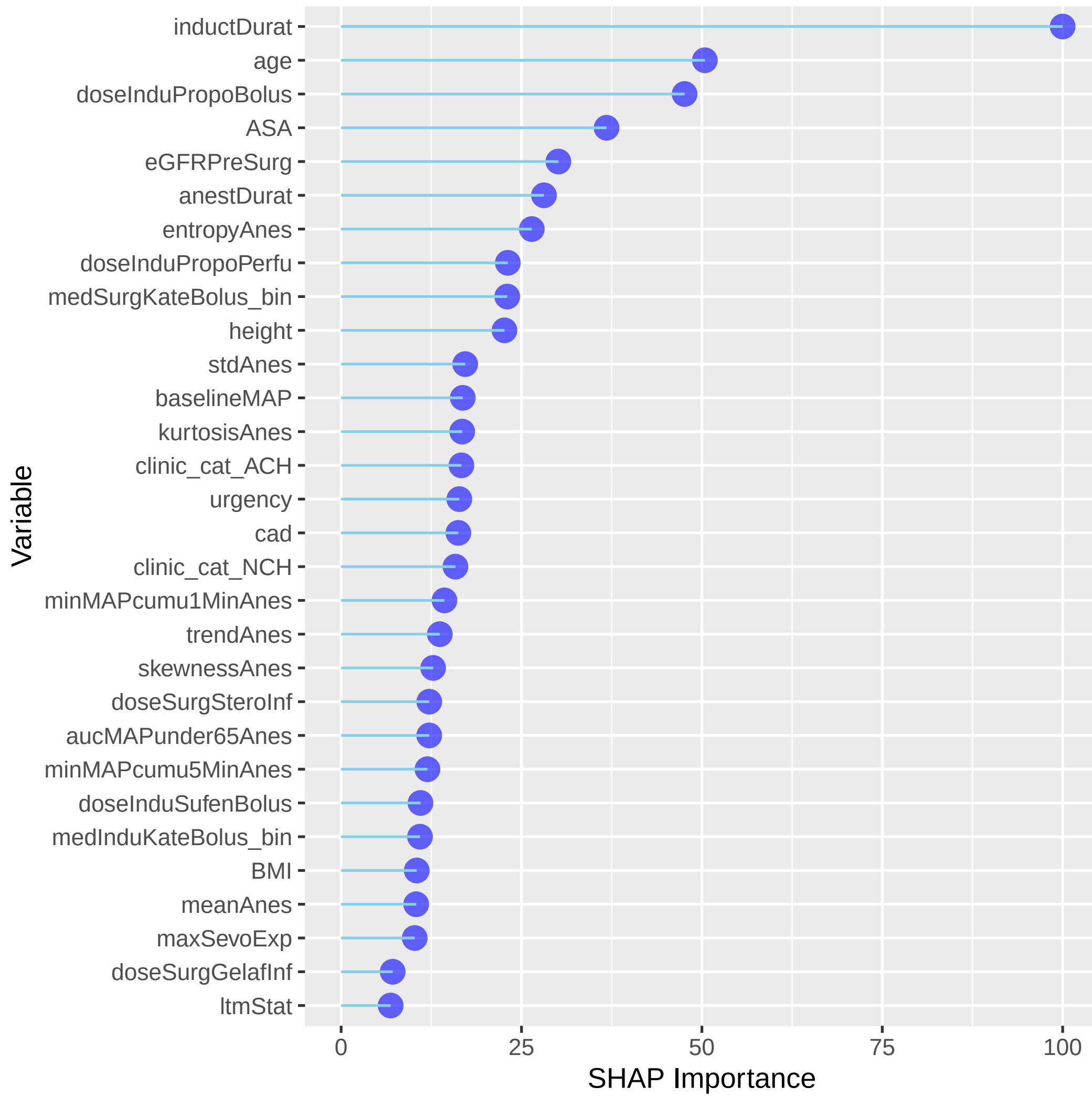

Figure 27. Scaled average Shapley values of the 30 most important predictors, assigned by a Random Forest Classifier (RFC), trained on a balanced development cohort for the prediction of post-operative acute kidney injury stage 2 after non-cardiac surgery. Shapley values were computed for all individual model predictions on the validation data set and then averaged to estimate overall feature contribution. All values are scaled to the Shapley value of the most important feature for better comparability.
Abbrev.: age, patient age; anestDurat, duration of anesthesia; ASA, American Society of Anesthesiology physical status; aucMAPunder65Anes, area under the curve for all MAP below 65mmHg during anesthesia; baselineMAP, first measured MAP value; BMI, body mass index of patient; cad, coronary heart disease; clinic_cat_ACH, general surgery; clinic_cat_NCH, neurology surgery; doseInduPropoPerfu, dose of propofol administered via perfusor during induction; doseInduSufenBolus, dose of sufentanil administered via bolus during induction; doseInduThiopBolus, dose of thiopental administered via bolus during induction; doseSurgGelafInf, dose of gelafundin administered via infusion during induction; doseSurgSteroInf, dose of sterofundin administered via infusion during induction; eGFRPreSurg, estimated glomerular filtration rate before surgery; entropyAnes, entropy of MAP during anesthesia; height, patient height; inductDurat, duration of induction of surgery; kurtosisAnes, kurtosis of MAP during anesthesia; ltmStat, longterm medication with statins; maxSevoExp, maximum expiratory sevoflurane measurement during induction; meanAnes, mean MAP during entire anesthesia; medInduKateBolus_bin, administration of catecholamine during induction; medSurgKateBolus_bin, administration of catecholamine during surgery; minMAPcumu5MinAnes, minimal MAP for 5 minutes during anesthesia; stdAnes, standard deviation of MAP during anesthesia; skewnessAnes, skewness of MAP during anesthesia; trendAnes, trend of MAP during anesthesia; urgency, level of urgency of surgical intervention.

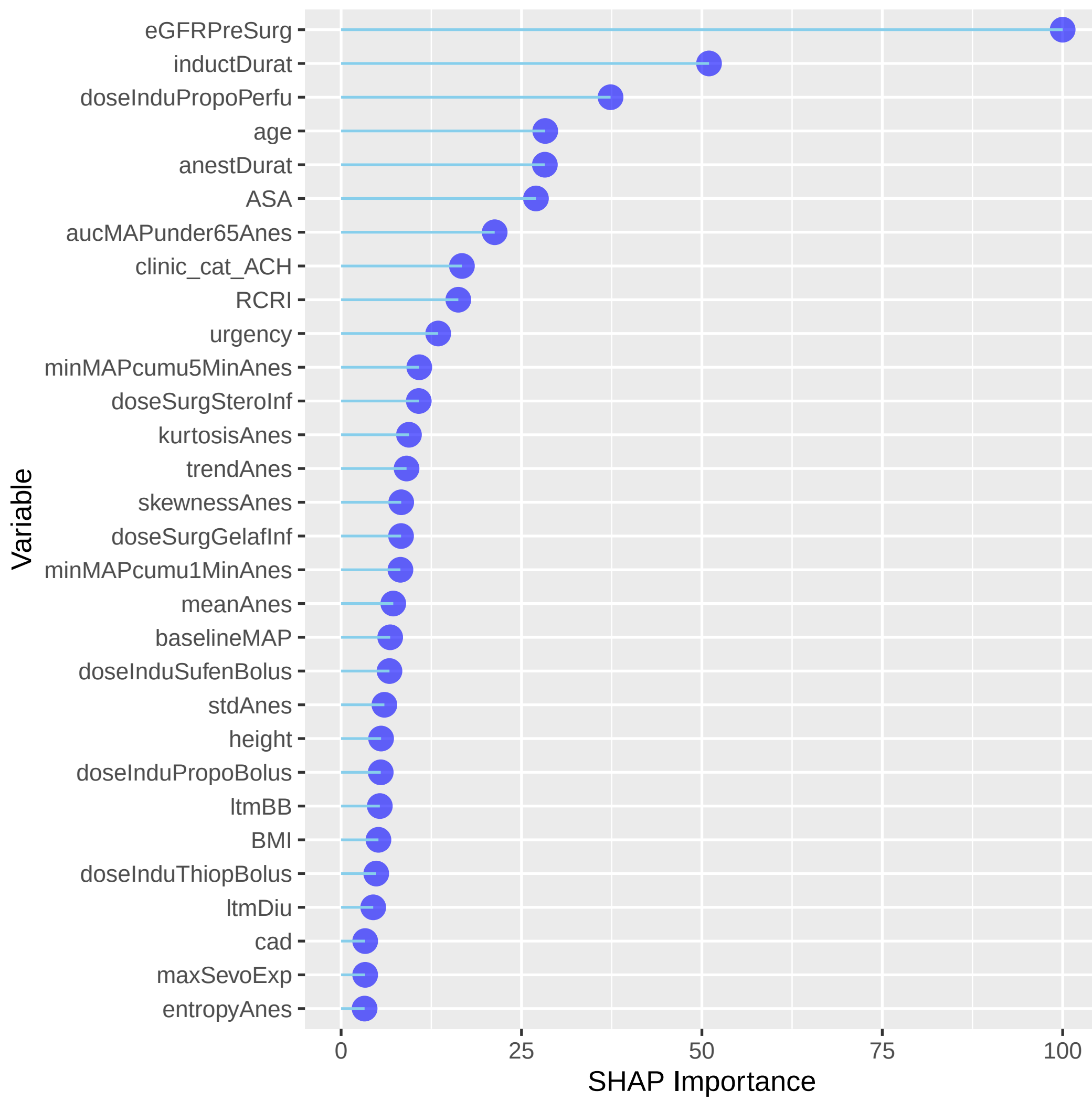

Figure 28. Scaled average Shapley values of the 30 most important predictors, assigned by a Random Forest Classifier (RFC), trained on a balanced development cohort for the prediction of post-operative acute kidney injury stage 3 after non-cardiac surgery. Shapley values were computed for all individual model predictions on the validation data set and then averaged to estimate overall feature contribution. All values are scaled to the Shapley value of the most important feature for better comparability.

Abbrev.: age, patient age; anestDurat, duration of anesthesia; ASA, American Society of Anesthesiology physical status; aucMAPunder65Anes, area under the curve for all MAP below 65mmHg during anesthesia; baselineMAP, first measured MAP value; BMI, body mass index of patient; clinic_cat_ACH, general surgery; doseInduEtomidateBolus, dose of etomidate administered via bolus during induction; doseInduPropoPerfu, dose of propofol administered via perfusor during induction; doseInduSufenBolus, dose of sufentanil administered via bolus during induction; doseInduThiopBolus, dose of thiopental administered via bolus during induction; doseSurgGelafInf, dose of gelafundin administered via infusion during induction; doseSurgSteroInf, dose of sterofundin administered via infusion during induction; eGFRPreSurg, estimated glomerular filtration rate before surgery; entropyAnes, entropy of MAP during anesthesia; height, patient height; inductDurat, duration of induction of surgery; kurtosisAnes, kurtosis of MAP during anesthesia; ltmBB, longterm medication with beta-blockers; maxSevoExp, maximum expiratory sevoflurane measurement during induction; meanAnes, mean MAP during entire anesthesia; medInduKateBolus_bin, administration of catecholamine during induction; minMAPcumu1MinAnes, minimal MAP for 1 minute during anesthesia; minMAPcumu5MinAnes, minimal MAP for 5 minutes during anesthesia; NYHA, New York Heart Association classification of heart disease; RCRI, revised cardiac risk index; stdAnes, standard deviation of MAP during anesthesia; skewnessAnes, skewness of MAP during anesthesia; trendAnes, trend of MAP during anesthesia; urgency, level of urgency of surgical intervention.

a

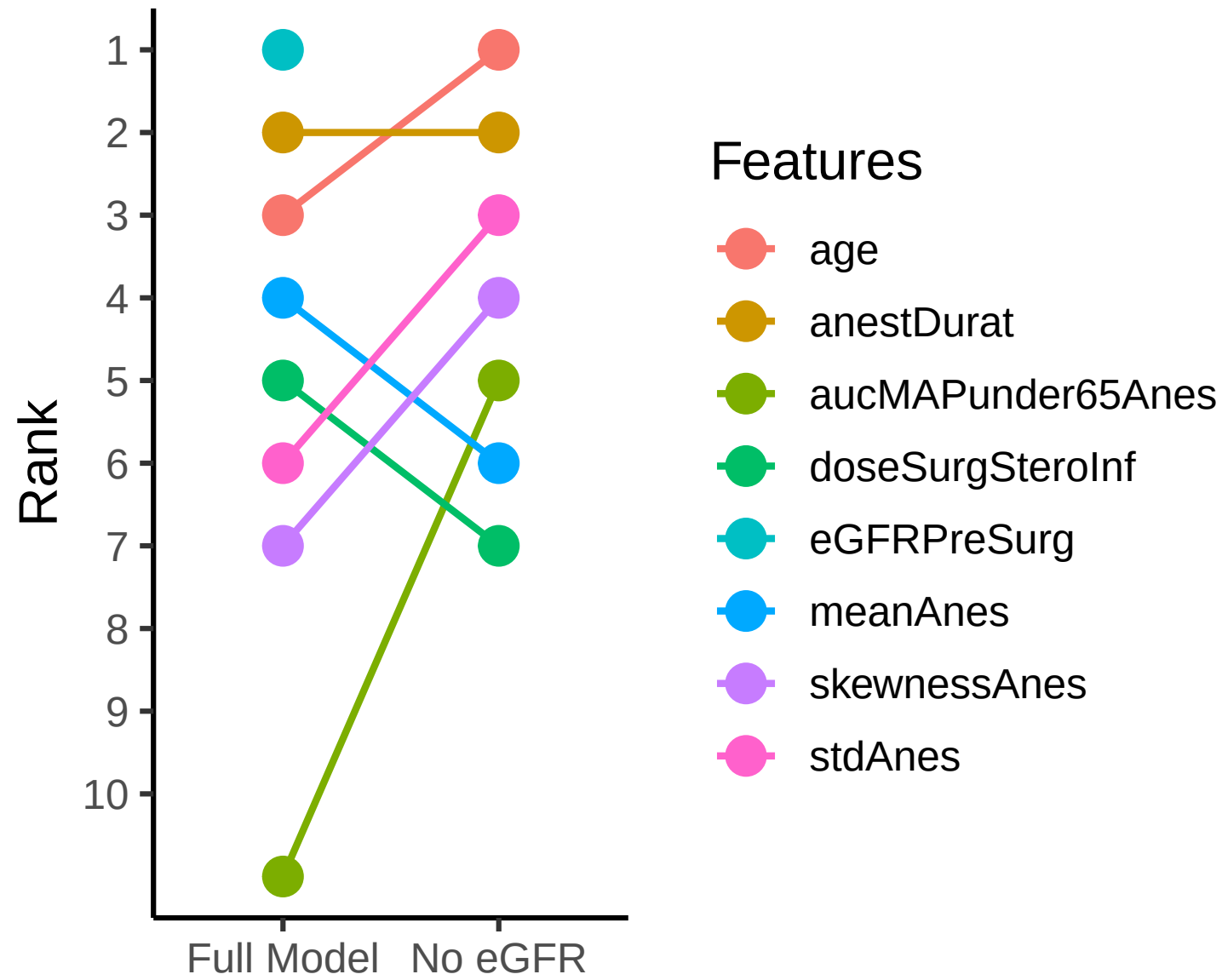

b

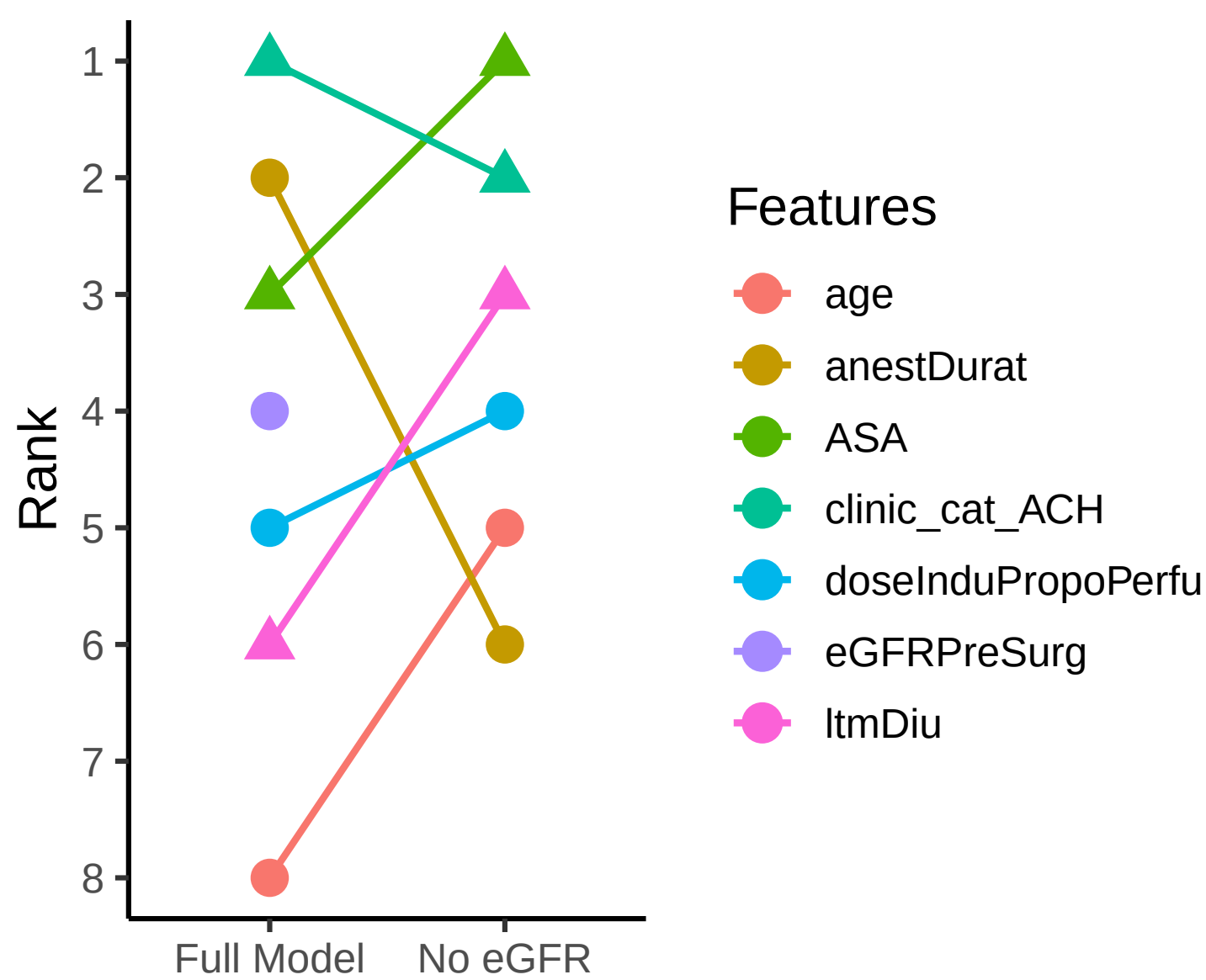

Figure 29. Ranks of native (a) and post-hoc assessed (b) Feature Importance (FI) assigned by PO-AKI prediction models trained on the balanced development cohort without preoperative eGFR measurements (left) in comparison to models trained on the original balanced development cohort with the complete predictor set (right). The FI ranks across all models (y-axis) are displayed for the most important features (ranks 1-5). Predictors were ranked based on the absolute native FI that was estimated by the respective model (a) and the largest average Shapley Additive Explanations Value assigned to a feature within 100 bootstrap replicates (b). Lower rank values indicate higher importance with rank 1 representing highest importance. Continuous features are shown as dots, categorical features are displayed as triangles, respectively.
Abbrev.: age, patient age; anestDurat, duration of anesthesia; ASA, American Society of Anesthesiology physical status; aucMAPunder65Anes, area under the curve for all MAP below 65mmHg during anesthesia; clinic_cat_ACH, general surgery; doseInduPropoPerfu, dose of propofol administered via perfusor during induction; doseSurgSteroInf, dose of sterofundin administered via infusion during induction; eGFRPreSurg, estimated glomerular filtration rate before surgery; ltmDiu, longterm medication with diuretics; meanAnes, mean MAP during entire anesthesia; skewnessAnes, skewness of MAP during anesthesia, stdAnes, standard deviation of MAP during anesthesia.

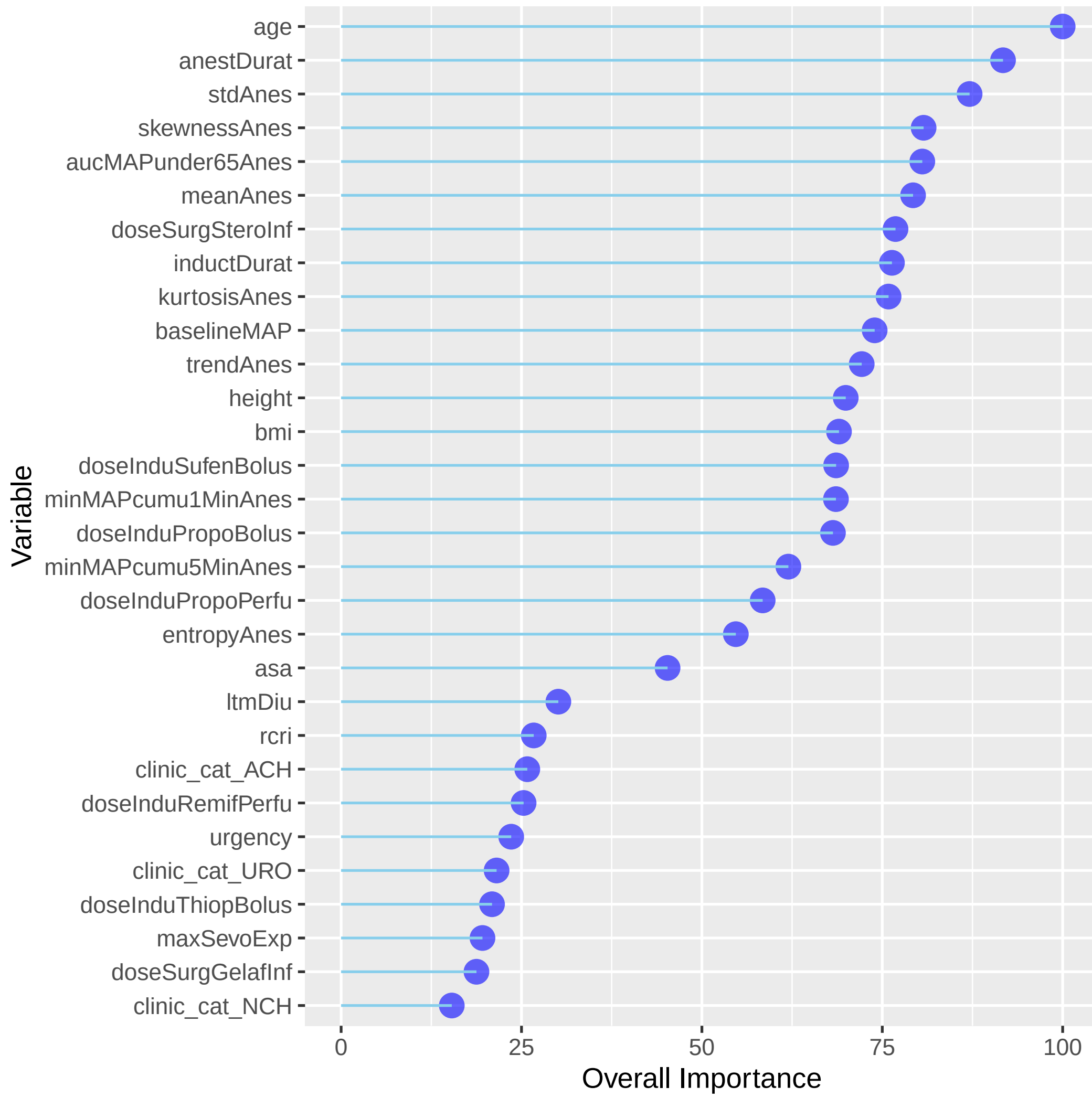

Figure 30. Scaled native Feature Importances (FIs) of the 30 most important predictors, assigned by a Random Forest Classifier (RFC) trained on the balanced development cohort without preoperative eGFR measurements.
FI measures are based on the averaged contribution of each feature to the total gain of this feature's splits. All weights are scaled to the highest FI, which is assigned a value of 100, for better comparability.
Abbrev.: asa, American Society of Anesthesiology physical status; age, patient age; anestDurat, duration of anesthesia; aucMAPunder65Anes, area under the curve for all MAP below 65mmHg during anesthesia; baselineMAP, first measured MAP value; bmi, Body Mass Index of patient; clinic_cat_ACH, general surgery; clinic_cat_NCH, neurology surgery; clinic_cat_URO, urology surgery; doseInduPropoBolus, dose of propofol administered via bolus during induction; doseInduPropoPerfu, dose of propofol administered via perfusor during induction; doseInduRemifPerfu, dose of remifentanil administered via perfusor during induction; doseInduSufenBolus, dose of sufentanil administered via bolus during induction; doseInduThiopBolus, dose of thiopental administered via bolus during induction; doseSurgGelafInf, dose of gelafundin administered via infusion during induction; doseSurgSteroInf, dose of sterofundin administered via infusion during induction; entropyAnes, entropy of MAP during anesthesia; height, patient height; inductDurat, duration of induction of surgery; kurtosisAnes, kurtosis of MAP during anesthesia; ltmDiu, longterm medication with diuretics; maxSevoExp, maximum expiratory sevoflurane measurement during induction; meanAnes, mean MAP during entire anesthesia; minMAPcumu1MinAnes, minimal MAP for 1 minute during anesthesia; minMAPcumu5MinAnes, minimal MAP for 5 minutes during anesthesia; rcri, revised cardiac risk index; skewnessAnes, skewness of MAP during anesthesia; stdAnes, standard deviation of MAP during anesthesia; trendAnes, trend of MAP during anesthesia; urgency, level of urgency of surgical intervention.

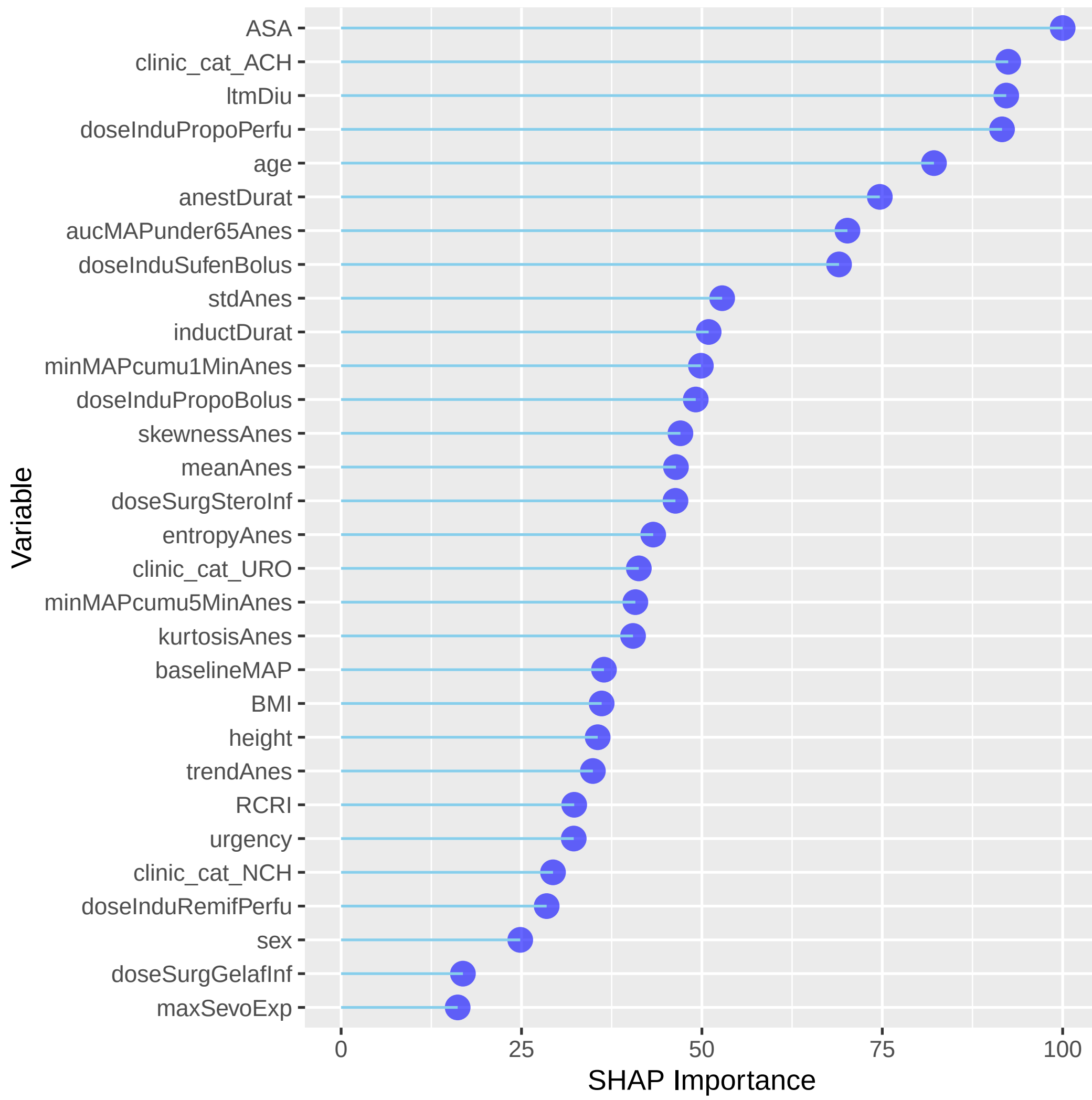

Figure 31. Scaled average Shapley values of the 30 most important predictors, assigned by a Random Forest Classifier (RFC) trained on the balanced development cohort without preoperative eGFR measurements. Shapley values were computed for all individual model predictions on the validation data set and then averaged to estimate overall feature contribution. All values are scaled to the Shapley value of the most important feature for better comparability.
Abbrev.: ASA, American Society of Anesthesiology physical status; age, patient age; anestDurat, duration of anesthesia; aucMAPunder65Anes, area under the curve for all MAP below 65mmHg during anesthesia; baselineMAP, first measured MAP value; BMI, Body Mass Index of patient; clinic_cat_ACH, general surgery; clinic_cat_NCH, neurology surgery; clinic_cat_URO, urology surgery; doseInduPropoBolus, dose of propofol administered via bolus during induction; doseInduPropoPerfu, dose of propofol administered via perfusor during induction; doseInduRemifPerfu, dose of remifentanil administered via perfusor during induction; doseInduSufenBolus, dose of sufentanil administered via bolus during induction; doseInduThiopBolus, dose of thiopental administered via bolus during induction; doseSurgGelafInf, dose of gelafundin administered via infusion during induction; doseSurgSteroInf, dose of sterofundin administered via infusion during induction; entropyAnes, entropy of MAP during anesthesia; height, patient height; inductDurat, duration of induction of surgery; kurtosisAnes, kurtosis of MAP during anesthesia; ltmDiu, longterm medication with diuretics; meanAnes, mean MAP during entire anesthesia; minMAPcumu1MinAnes, minimal MAP for 1 minute during anesthesia; minMAPcumu5MinAnes, minimal MAP for 5 minutes during anesthesia; RCRI, revised cardiac risk index; sex, biological sex of patient; skewnessAnes, skewness of MAP during anesthesia; stdAnes, standard deviation of MAP during anesthesia; trendAnes, trend of MAP during anesthesia; urgency, level of urgency of surgical intervention.

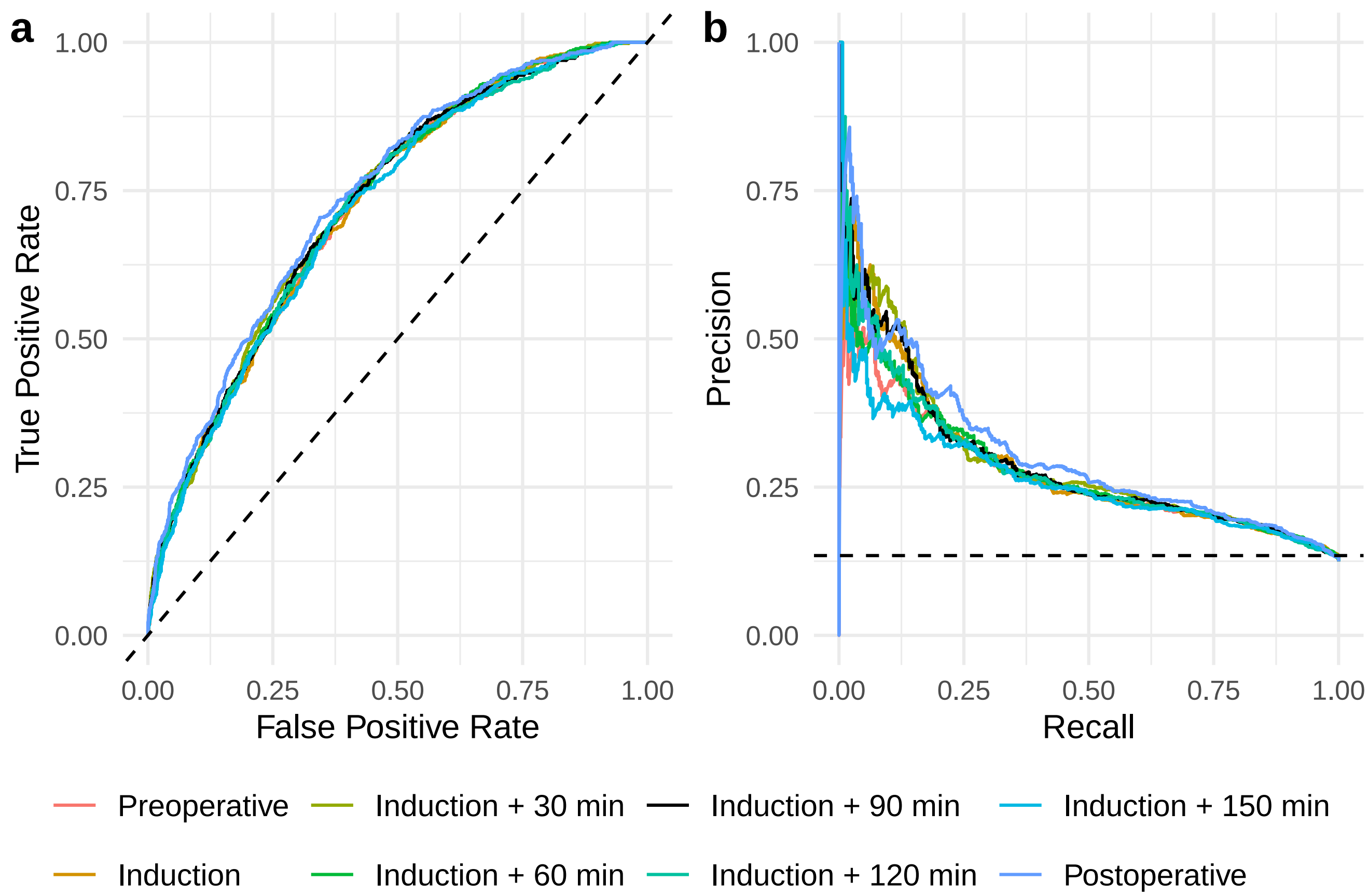

Figure 32. Receiver Operating Characteristics and Precision-Recall curves of Random Forest classifiers (RFCs), trained on the balanced development data set at different times points of anesthesia, and evaluated on the validation data set. The dashed lines indicate the expected curves for a random classifier, given for the Precision-Recall curve by the outcome distribution within the validation dataset (12.45% PO-AKI occurrence).
Abbrev.: SVM, Support Vector Machine classifier; xGBM, extreme Gradient Boosting Machine classifier.

3 Supplementary Tables

Table 1. Comparison of the complete cohort and the final study cohort. Baseline characteristics of all patients meeting the study protocol eligibility criteria who provided written informed consent (complete study cohort) before application of analysis-specific exclusion criteria, data quality criteria, and data cleaning, and the final study cohort included in model development (see Supplementary File S1: Study population selection). Continuous variables are presented as mean ± SD, whereas categorical and binary variables are presented as frequency (%). Missing values are reported separately whenever present. Differences between cohorts are described using the absolute standardized mean difference (SMD). Abbrev.: ASA, American Society of Anesthesiologists physical status; BMI, body mass index; eGFR, estimated glomerular filtration rate; NYHA, New York Heart Association; PO-AKI, postoperative acute kidney injury; RCRI, Revised Cardiac Risk Index; SD, standard deviation; SMD, standardized mean difference.

**Variable**	**Complete cohort** (n = 111,345)	**Final study cohort** (n = 13,890)	**Absolute SMD**
**Outcome**			
PO-AKI	9,715 (19.7%)	1,718 (12.4%)	0.2003
Missing	61,979 (55.7%)	0 (0.0%)	
**Demography**			
Age [years]	56.59 ± 18.20	58.77 ± 17.29	0.1228
Height [cm]	171.85 ± 55.23	171.81 ± 9.81	0.0010
Missing	4,377 (3.9%)	0 (0.0%)	
Weight [kg]	79.38 ± 34.89	79.25 ± 18.76	0.0044
Missing	2,428 (2.2%)	0 (0.0%)	
BMI [kg/m^2]	27.11 ± 23.84	26.75 ± 5.59	0.0208
Missing	4,490 (4.0%)	0 (0.0%)	
Gender (male)	57,427 (51.6%)	7,407 (53.3%)	0.0351
**Preoperative risk assessment**			
RCRI			0.4961
0	37,700 (33.9%)	2,088 (15.0%)	
1	49,747 (44.7%)	9,016 (64.9%)	
2	14,985 (13.5%)	2,049 (14.8%)	
3	8,913 (8.0%)	737 (5.3%)	
Surgical urgency levels			0.0976
Elective	92,923 (83.5%)	11,234 (80.9%)	
Urgent	9,553 (8.6%)	1,439 (10.4%)	
Emergency	6,126 (5.5%)	966 (7.0%)	
Immediate	2,730 (2.5%)	251 (1.8%)	
Missing	13 (0.0%)	0 (0.0%)	
ASA			0.1598
1	21,6156 (14.5%)	1,367 (9.8%)	
2	52,335 (47.0%)	6,765 (48.7%)	
3	38,043 (34.2%)	5,286 (38.1%)	
4	4,686 (4.2%)	468 (3.4%)	
5	108 (0.1%)	4 (0.0%)	
6	4 (0.0%)	0 (0.0%)	
Missing	13 (0.0%)	0 (0.0%)	

**Variable**	**Complete cohort** (n = 111,345)	**Final study cohort** (n = 13,890)	**Absolute SMD**
Estimated glomerular filtration rate preoperative [mL/min/1.73 m^2]	82.56 ± 28.16	84.07 ± 25.05	0.0567
Missing	8,217 (7.4%)	0 (0.0%)	
**Surgical department**			
General surgery	10,611 (9.5%)	4,113 (29.6%)	0.5232
Vascular surgery	1,819 (1.6%)	512 (3.7%)	0.1278
Cardiac surgery	8,123 (7.3%)	600 (4.3%)	0.1275
Otolaryngology surgery	12,334 (11.1%)	415 (3.0%)	0.3204
Neurosurgery	6,696 (6.0%)	1,659 (11.9%)	0.2086
Thoracic surgery	1,606 (1.4%)	468 (3.4%)	0.1260
Trauma surgery	10,498 (9.4%)	1,643 (11.8%)	0.0779
Urology surgery	7,648 (6.9%)	813 (5.9%)	0.0416
Other surgery department	40,080 (36.0%)	3,369 (24.3%)	0.2580
**Duration of the anesthesia process**			
Duration of induction [min]	17.90 ± 21.37	25.29 ± 15.49	0.3959
Duration of anesthesia [min]	156.75 ± 118.08	207.08 ± 107.79	0.4452
**Pre-existing conditions**			
NYHA			0.1018
0	102,015 (91.6%)	13,038 (93.9%)	
1	526 (0.5%)	63 (0.5%)	
2	4,524 (4.1%)	449 (3.2%)	
3	3,657 (3.3%)	317 (2.3%)	
4	623 (0.6%)	23 (0.2%)	
Chronic heart failure	5,758 (5.2%)	481 (3.5%)	0.0841
Coronary heart disease	11,833 (10.6%)	1,243 (8.9%)	0.0565
Cerebrovascular disease	5,128 (4.6%)	765 (5.5%)	0.0412
Peripheral arterial disease	4,137 (3.7%)	566 (4.1%)	0.0186
Diabetes	9,551 (8.6%)	1,252 (9.0%)	0.0154
Liver cirrhosis	1,280 (1.1%)	307 (2.2%)	0.0826
Atrial fibrillation	5,822 (5.2%)	695 (5.0%)	0.0102
**Continuous medication**			
ACE inhibitors	21,813 (19.6%)	2,858 (20.6%)	0.0246
Sartans	17,002 (15.3%)	2,210 (15.9%)	0.0177
Beta-blockers	26,870 (24.1%)	3,505 (25.2%)	0.0256
Diuretics	20,695 (18.6%)	2,625 (18.9%)	0.0080
Statins	18,283 (16.4%)	2,360 (17.0%)	0.0153
Biguanides	5,678 (5.1%)	819 (5.9%)	0.0350
Insulins	5,875 (5.3%)	686 (4.9%)	0.0153
Calcium channel blockers	12,951 (11.6%)	1,716 (12.4%)	0.0222

Table 2. Baseline characteristics of the balanced development cohort. Summary statistics are presented as mean ± SD for continuous variables and frequency (%) for categorical/binary variables, respectively. The β coefficients were determined using logistic regression analysis. Group differences were tested using t-tests for continuous and chi-square tests for categorical/binary variables, respectively. Bonferroni adjusted p-values <0.05 were considered statistically significant, corresponding to a confidence level of 95%.
Abbrev.: age, patient age; ASA, American Society of Anesthesiology physical status; AUC, area under the curve; BMI, Body Mass Index of patient; height, patient height; MAP, mean arterial pressure; NYHA, New York Heart Association classification of heart disease; RCRI, Revised Cardiac Risk Index.

**Variable**	**No PO-AKI** (n = 1,143)	**PO-AKI** (n = 1,143)	β	**Adjusted** p**-value**
**Demography**				
Age [years]	57.71 ± 17.47	61.72 ± 15.22	0.015	<0.001
Height [cm]	171.54 ± 9.93	172.01 ± 9.68	0.005	1.0000
BMI [kg/m²]	26.78 ± 5.92	27.38 ± 6.24	0.016	1.0000
Gender (male)	567 (49.6%)	694 (60.7%)	0.451	<0.001
**Preoperative risk assessment**				
RCRI				
0	159 (13.9%)	108 (9.4%)	-0.437	0.0718
1	783 (68.5%)	707 (61.9%)	-0.294	0.0645
2	149 (13.0%)	239 (20.9%)	0.567	<0.001
3	52 (4.5%)	89 (7.8%)	0.572	0.1103
Surgical urgency levels				
Elective	954 (83.5%)	845 (73.9%)	-0.577	<0.001
Urgent	105 (9.2%)	138 (12.1%)	0.306	1.0000
Emergency	72 (6.3%)	129 (11.3%)	0.638	0.0024
Immediate	12 (1.0%)	31 (2.7%)	0.966	0.3605
ASA				
1	120 (10.5%)	66 (5.8%)	-0.649	0.0035
2	593 (51.9%)	430 (37.6%)	-0.581	<0.001
3	400 (35.0%)	555 (48.6%)	0.562	<0.001
4	30 (2.6%)	90 (7.9%)	1.154	<0.001
5	0 (0.0%)	2 (0.2%)	12.231	1.0000
Estimated glomerular filtration rate preoperative [mL/min/1.73 m²]	86.02 ± 23.45	75.41 ± 30.07	-0.015	<0.001
Indwelling urinary catheter	556 (48.6%)	609 (53.3%)	0.186	1.0000
Stomach tube	637 (55.7%)	650 (56.9%)	0.046	1.0000
Baseline MAP (start of induction) [mm Hg]	98.22 ± 19.29	98.91 ± 21.72	0.002	1.0000
**Pre-existing conditions**				
NYHA				
0	1,079 (94.4%)	1,044 (91.3%)	-0.469	0.3560
1	4 (0.3%)	5 (0.4%)	0.224	1.0000
2	40 (3.5%)	46 (4.0%)	0.145	1.0000
3	17 (1.5%)	44 (3.8%)	0.975	0.0548

	No PO-AKI (n = 1,143)	PO-AKI (n = 1,143)	β	Adjusted p-value
4	3 (0.3%)	4 (0.3%)	0.289	1.0000
Chronic heart failure	37 (3.2%)	64 (5.6%)	0.572	0.4966
Coronary Heart Disease	83 (7.3%)	141 (12.3%)	0.586	0.0041
Cerebrovascular disease	57 (5.0%)	51 (4.5%)	-0.117	1.0000
Peripheral Arterial Disease	42 (3.7%)	51 (4.5%)	0.202	1.0000
Diabetes	89 (7.8%)	133 (11.6%)	0.444	0.1506
Liver Cirrhosis	15 (1.3%)	59 (5.2%)	1.409	<0.001
Atrial fibrillation	47 (4.1%)	74 (6.5%)	0.479	0.9269
**Surgical department**				
General surgery	336 (29.4%)	433 (37.9%)	0.382	0.0014
Vascular surgery	44 (3.8%)	30 (2.6%)	-0.396	1.0000
Cardiac surgery	40 (3.5%)	70 (6.1%)	0.587	0.2839
Otolaryngology surgery	38 (3.3%)	16 (1.4%)	-0.885	0.2469
Neurology surgery	140 (12.2%)	52 (4.5%)	-1.075	<0.001
Plastic surgery	15 (1.3%)	23 (2.0%)	0.435	1.0000
Thoracic surgery	46 (4.0%)	24 (2.1%)	-0.670	0.6490
Trauma surgery	137 (12.0%)	111 (9.7%)	-0.236	1.0000
Urology surgery	57 (5.0%)	131 (11.5%)	0.903	<0.001
Other surgery department	290 (25.4%)	253 (22.1%)	-0.179	1.0000
**Duration of the anesthesia process**				
Duration of induction [min]	25.48 ± 14.43	27.52 ± 18.48	0.008	0.2724
Duration of anesthesia [min]	209.83 ± 111.58	218.55 ± 106.00	0.001	1.0000
**Continuous medication**				
ACE inhibitors	222 (19.4%)	263 (23.0%)	0.215	1.0000
Sartans	163 (14.3%)	234 (20.5%)	0.437	0.0072
Beta-blockers	270 (23.6%)	356 (31.1%)	0.380	0.0043
Calcium channel blockers	123 (10.8%)	177 (15.5%)	0.418	0.0654
Biguanides	78 (6.8%)	104 (9.1%)	0.312	1.0000
Insulins	59 (5.2%)	79 (6.9%)	0.311	1.0000
Diuretics	173 (15.1%)	360 (31.5%)	0.947	<0.001
Statins	169 (14.8%)	218 (19.1%)	0.306	0.4798
**Anesthesia administration**				
Catecholamine bolus (induction)	431 (37.7%)	402 (35.2%)	-0.110	1.0000
Atropin bolus (induction)	74 (6.5%)	77 (6.7%)	0.043	1.0000
Catecholamine bolus (surgery)	550 (48.1%)	470 (41.1%)	-0.284	0.0578
Etomidate bolus (induction) [µg/kg bw/min i.v.]	23.31 ± 85.18	32.73 ± 99.13	0.001	1.0000
Propofol bolus (induction) [mg/kg bw i.v.]	1.84 ± 1.14	1.60 ± 1.17	-0.178	<0.001
Propofol perfusor (induction) [mg/kg bw/h i.v.]	1.55 ± 2.35	0.85 ± 1.93	-0.153	<0.001
Remifentanil perfusor (induction) [µg/kg bw/min i.v.]	3.56 ± 7.51	2.60 ± 7.03	-0.018	0.1371

	**No PO-AKI** (n = 1,143)	**PO-AKI** (n = 1,143)	β	**Adjusted** p**-value**
Sufentanil bolus (induction) [µg/kg bw i.v.]	0.27 ± 0.19	0.31 ± 0.21	0.909	0.0013
Sufentanil Perfusor (induction) [µg/kg bw/h i.v.]	0.04 ± 0.15	0.01 ± 0.08	-2.595	<0.001
Thiopental bolus (induction) [mg/kg bw]	1.01 ± 2.32	1.21 ± 2.46	0.035	1.0000
Gelafundin Infusion (surgery) [ml/kg bw/h]	1.33 ± 4.01	1.82 ± 4.50	0.028	0.5185
Sterofundin Infusion (surgery) [ml/kg bw/h]	8.02 ± 6.50	7.62 ± 6.28	-0.010	1.0000
Max. expiratory sevoflurane concentration [%]	0.24 ± 0.18	0.25 ± 0.18	0.434	1.0000
**MAP features**				
Lowest MAP value for 1 minute [mmHg]	50.20 ± 13.87	47.79 ± 14.02	-0.012	0.0031
Lowest MAP value for 5 cumulative minutes [mmHg]	58.10 ± 8.77	56.07 ± 9.45	-0.025	<0.001
AUC for MAP values <65mmHg	206.75 ± 307.56	297.83 ± 472.44	0.001	<0.001
Mean	78.59 ± 7.97	78.19 ± 8.35	-0.006	1.0000
Standard deviation	12.93 ± 4.02	13.60 ± 4.39	0.038	0.0131
Entropy	0.92 ± 0.03	0.93 ± 0.03	3.006	1.0000
Trend	-0.06 ± 0.25	-0.08 ± 0.23	-0.421	1.0000
Kurtosis	6.06 ± 6.95	5.54 ± 5.76	-0.013	1.0000
Skewness	0.85 ± 1.04	0.77 ± 0.96	-0.087	1.0000

Table 3. Baseline characteristics of validation cohort. Summary statistics are presented as mean ± SD for continuous variables and frequency (%) for categorical/binary variables, respectively. The β coefficients were determined using logistic regression analysis. Group differences were tested using t-tests for continuous and chi-square tests for categorical/binary variables, respectively. Bonferroni adjusted p-values <0.05 were considered statistically significant, corresponding to a confidence level of 95%. Abbrev.: age, patient age; ASA, American Society of Anesthesiology physical status; AUC, area under the curve; BMI, body mass index of patient; height, patient height; MAP, mean arterial pressure; NYHA, New York Heart Association classification of heart disease; RCRI, Revised Cardiac Risk Index.

	**No PO-AKI** (n = 4,055)	**PO-AKI** (n = 575)	β	**Adjusted p-value**
**Demography**				
Age [years]	58.00 ± 17.53	63.48 ± 14.71	0.020	<0.001
Height [cm]	172.01 ± 9.83	171.70 ± 9.16	-0.003	1.0000
BMI [kg/m²]	26.83 ± 5.62	26.64 ± 5.34	-0.006	1.0000
Gender (male)	2,158 (53.2%)	335 (58.3%)	0.205	1.0000
**Preoperative risk assessment**				
RCRI				
0	650 (16.0%)	52 (9.0%)	-0.652	0.0013
1	2,664 (65.7%)	348 (60.5%)	-0.223	1.0000
2	561 (13.8%)	127 (22.1%)	0.569	<0.001
3	180 (4.4%)	48 (8.3%)	0.673	0.0051
Surgical urgency levels				
Elective	3,313 (81.7%)	418 (72.7%)	-0.517	<0.001
Urgent	396 (9.8%)	77 (13.4%)	0.357	0.5616
Emergency	280 (6.9%)	54 (9.4%)	0.335	1.0000
Immediate	66 (1.6%)	26 (4.5%)	1.052	<0.001
ASA				
1	454 (11.2%)	30 (5.2%)	-0.829	0.0015
2	2,008 (49.5%)	203 (35.3%)	-0.587	<0.001
3	1,487 (36.7%)	302 (52.5%)	0.647	<0.001
4	104 (2.6%)	40 (7.0%)	1.044	<0.001
5	2 (0.0%)	0 (0.0%)	-23.636	1.0000
Estimated glomerular filtration rate preoperative [mL/min/1.73 m²]	86.11 ± 23.69	74.89 ± 29.35	-0.018	<0.001
Indwelling urinary catheter	1986 (49.0%)	314 (54.6%)	0.226	0.8697
Stomach tube	2,309 (56.9%)	346 (60.2%)	0.133	1.0000
Baseline MAP (Start of induction) [mm Hg]	98.97 ± 20.12	96.70 ± 19.99	-0.006	0.8346
**Pre-existing conditions**				
NYHA				
0	3,828 (94.4%)	530 (92.2%)	-0.359	1.0000
1	19 (0.5%)	2 (0.3%)	-0.299	1.0000
2	120 (3.0%)	26 (4.5%)	0.440	1.0000
3	84 (2.1%)	17 (3.0%)	0.365	1.0000
4	4 (0.1%)	0 (0.0%)	-10.518	1.0000

Variable	No PO-AKI (n = 4,055)	PO-AKI (n = 575)	β	Adjusted p-value
Chronic heart failure	130 (3.2%)	22 (3.8%)	0.183	1.0000
Coronary Heart Disease	341 (8.4%)	68 (11.8%)	0.379	0.5393
Cerebrovascular disease	217 (5.4%)	39 (6.8%)	0.252	1.0000
Peripheral Arterial Disease	178 (4.4%)	30 (5.2%)	0.181	1.0000
Diabetes	362 (8.9%)	66 (11.5%)	0.280	1.0000
Liver Cirrhosis	65 (1.6%)	35 (6.1%)	1.381	<0.001
Atrial fibrillation	190 (4.7%)	32 (5.6%)	0.181	1.0000
**Surgical department**				
General surgery	1,110 (27.4%)	229 (39.8%)	0.563	<0.001
Vascular surgery	178 (4.4%)	15 (2.6%)	-0.539	1.0000
Cardiac surgery	166 (4.1%)	35 (6.1%)	0.418	1.0000
Otolaryngology surgery	130 (3.2%)	9 (1.6%)	-0.734	1.0000
Neurology surgery	557 (13.7%)	14 (2.4%)	-1.853	<0.001
Plastic surgery	84 (2.1%)	10 (1.7%)	-0.178	1.0000
Thoracic surgery	136 (3.4%)	19 (3.3%)	-0.015	1.0000
Trauma surgery	508 (12.5%)	54 (9.4%)	-0.323	1.0000
Urology surgery	212 (5.2%)	56 (9.7%)	0.671	0.0015
Other surgery department	974 (24.0%)	134 (23.3%)	-0.040	1.0000
**Duration of the anesthesia process**				
Duration of induction [min]	24.96 ± 15.40	28.18 ± 16.63	0.012	<0.001
Duration of anesthesia [min]	205.08 ± 107.15	213.44 ± 104.65	0.001	1.0000
**Continuous medication**				
ACE inhibitors	807 (19.9%)	154 (26.8%)	0.387	0.0113
Sartans	628 (15.5%)	100 (17.4%)	0.139	1.0000
Beta-blockers	958 (23.6%)	178 (31.0%)	0.372	0.0105
Calcium channel blockers	465 (11.5%)	77 (13.4%)	0.177	1.0000
Biguanides	224 (5.5%)	37 (6.4%)	0.162	1.0000
Insulins	179 (4.4%)	46 (8.0%)	0.633	0.0171
Diuretics	676 (16.7%)	165 (28.7%)	0.699	<0.001
Statins	666 (16.4%)	107 (18.6%)	0.151	1.0000
**Anesthesia administration**				
Catecholamine bolus (induction)	1,490 (36.7%)	212 (36.9%)	0.005	1.0000
Atropin bolus (induction)	234 (5.8%)	30 (5.2%)	-0.107	1.0000
Catecholamine bolus (surgery)	1,914 (47.2%)	235 (40.9%)	-0.257	0.3345
Etomidate bolus (induction) [µg/kg bw/min i.v.]	19.92 ± 76.55	31.60 ± 95.31	0.002	0.0803
Propofol bolus (induction) [mg/kg bw i.v.]	1.87 ± 1.14	1.58 ± 1.16	-0.217	<0.001
Propofol perfusor (induction) [mg/kg bw/h i.v.]	1.54 ± 2.33	0.81 ± 1.77	-0.176	<0.001
Remifentanil perfusor (induction) [µg/kg bw/min i.v.]	3.70 ± 7.66	2.38 ± 6.28	-0.028	0.0070
Sufentanil bolus (induction) [µg/kg bw i.v.]	0.26 ± 0.19	0.30 ± 0.20	1.164	<0.001

**Variable**	**No PO-AKI** (n = 4,055)	**PO-AKI** (n = 575)	β	**Adjusted p-value**
Sufentanil Perfusor (induction) [µg/kg bw/h i.v.]	0.04 ± 0.14	0.01 ± 0.06	-4.196	<0.001
Thiopental bolus (induction) [mg/kg bw]	0.97 ± 2.25	1.30 ± 2.58	0.056	0.1114
Gelafundin Infusion (surgery) [ml/kg bw/h]	1.19 ± 3.55	1.89 ± 4.43	0.043	0.0020
Sterofundin Infusion (surgery) [ml/kg bw/h]	7.90 ± 6.53	8.50 ± 7.25	0.013	1.0000
Max. expiratory sevoflurane concentration [%]	0.23 ± 0.18	0.26 ± 0.17	0.815	0.0855
**MAP features**				
Lowest MAP value for 1 minute [mmHg]	50.61 ± 13.35	47.49 ± 13.44	-0.017	<0.001
Lowest MAP value for 5 cumulative minutes [mmHg]	58.09 ± 8.85	55.62 ± 9.10	-0.031	<0.001
AUC for MAP values <65mmHg	200.84 ± 308.87	298.05 ± 432.11	0.001	<0.001
Mean	78.69 ± 8.20	77.36 ± 7.92	-0.021	0.0187
Standard deviation	12.99 ± 4.15	13.22 ± 4.02	0.013	1.0000
Entropy	0.92 ± 0.04	0.92 ± 0.04	0.381	1.0000
Trend	-0.06 ± 0.24	-0.07 ± 0.23	-0.223	1.0000
Kurtosis	5.95 ± 6.64	5.44 ± 5.71	-0.014	1.0000
Skewness	0.86 ± 1.01	0.73 ± 0.96	-0.135	0.2925

Table 4. Model performances of eight different machine learning algorithms in the resampling approach. Mean and standard deviation of different performance metrics, estimated in 100 different splits of the balanced (Bal) or original (Unbal) development data set, respectively, into training and test data sets to estimate training set dependent performance variance.
Abbrev.: BACC, Balanced Accuracy; Bal, balanced development data set; Neg Pred Value, negative predictive value; RFC, Random Forest Classifier; ROC-AUC, area under receiver operating characteristics curve; SVM, Support Vector Machine; Unbal, original development data set; xGBM, extreme Gradient Boosting Machine.

Data	Model	ROC-AUC	Sensitivity	Specificity	Neg Pred Value	Precision	F1-score	BACC
Bal	Group LASSO	0.718 ± 0.022	0.653 ± 0.031	0.661 ± 0.031	0.656 ± 0.024	0.659 ± 0.025	0.656 ± 0.025	0.657 ± 0.023
Bal	LASSO	0.719 ± 0.022	0.647 ± 0.030	0.670 ± 0.030	0.655 ± 0.022	0.662 ± 0.024	0.654 ± 0.023	0.659 ± 0.022
Bal	Ridge	0.720 ± 0.020	0.652 ± 0.032	0.675 ± 0.028	0.660 ± 0.023	0.667 ± 0.022	0.659 ± 0.024	0.663 ± 0.021
Bal	Elastic Net	0.719 ± 0.021	0.649 ± 0.031	0.670 ± 0.029	0.657 ± 0.022	0.663 ± 0.022	0.656 ± 0.023	0.659 ± 0.021
Bal	SVM	0.731 ± 0.021	0.665 ± 0.030	0.673 ± 0.028	0.668 ± 0.021	0.671 ± 0.021	0.667 ± 0.021	0.669 ± 0.020
Bal	Linear xGBM	0.707 ± 0.023	0.638 ± 0.033	0.656 ± 0.030	0.645 ± 0.022	0.650 ± 0.021	0.643 ± 0.023	0.647 ± 0.020
Bal	Tree xGBM	0.722 ± 0.023	0.657 ± 0.034	0.661 ± 0.030	0.659 ± 0.026	0.660 ± 0.023	0.658 ± 0.025	0.659 ± 0.023
Bal	RFC	0.740 ± 0.022	0.679 ± 0.030	0.670 ± 0.027	0.676 ± 0.022	0.673 ± 0.020	0.675 ± 0.022	0.674 ± 0.020
Unbal	Group LASSO	0.662 ± 0.098	0.034 ± 0.025	0.995 ± 0.004	0.879 ± 0.008	0.488 ± 0.105	0.084 ± 0.029	0.515 ± 0.011
Unbal	LASSO	0.679 ± 0.086	0.036 ± 0.023	0.995 ± 0.004	0.879 ± 0.008	0.500 ± 0.133	0.085 ± 0.025	0.516 ± 0.010
Unbal	Ridge	0.679 ± 0.083	0.023 ± 0.022	0.997 ± 0.003	0.878 ± 0.008	0.452 ± 0.174	0.064 ± 0.030	0.510 ± 0.009
Unbal	Elastic Net	0.681 ± 0.084	0.036 ± 0.024	0.995 ± 0.004	0.879 ± 0.008	0.498 ± 0.122	0.084 ± 0.026	0.515 ± 0.010
Unbal	SVM	0.707 ± 0.017	0.056 ± 0.015	0.993 ± 0.003	0.882 ± 0.002	0.540 ± 0.103	0.101 ± 0.025	0.525 ± 0.007
Unbal	Linear xGBM	0.705 ± 0.016	0.105 ± 0.019	0.982 ± 0.004	0.887 ± 0.007	0.456 ± 0.065	0.169 ± 0.027	0.543 ± 0.009
Unbal	Tree xGBM	0.729 ± 0.017	0.064 ± 0.018	0.993 ± 0.002	0.883 ± 0.007	0.564 ± 0.100	0.115 ± 0.029	0.529 ± 0.009
Unbal	RFC	0.731 ± 0.017	0.057 ± 0.015	0.994 ± 0.002	0.882 ± 0.002	0.575 ± 0.103	0.103 ± 0.026	0.526 ± 0.007

Table 5. Native Feature Importances (FI) of final classification models trained on the balanced development cohort. For each model, the FI was extracted from the respective classifier. Shown are the scaled native FI values and their respective ranks (in parentheses) for each model. Features are ordered by their mean ranks across all models. In case an FI could not be evaluated for a specific feature due to missing values, the corresponding FI value was set to 0.
Abbrev.: age, patient age; ASA, American Society of Anesthesiology physical status; AUC, area under the curve; AucMAPunder65Anes, area under the Curve for all MAP below 65mmHg during anesthesia; BMI, body mass index of patient; DoseInduEtomidateBolus, dose of etomidate administered via bolus during induction; DoseInduPropoBolus, dose of propofol administered via bolus during induction; DoseInduPropoPerfu, dose of propofol administered via perfusor during induction DoseInduRemifPerfu, dose of remifentanil administered via perfusor during induction DoseInduSufenBolus, dose of sufentanil administered via bolus during induction; DoseInduSufenPerfu, dose of sufentanil administered via perfusor during induction; DoseInduThiopBolus, dose of thiopental administered via bolus during induction; DoseSurgGelafInf, dose of gelafundin administered via infusion during induction; DoseSurgSteroInf, dose of sterofundin administered via infusion during induction; EGFRPreSurg, estimated Glomerular Filtration Rate before surgery; height, patient height; MAP, mean arterial pressure; MaxSevoExp, maximum expiratory sevoflurane measurement during induction; MedInduKateBolus_bin, administration of catecholamine during induction; MedSurgAtroBolus_bin, administration of atropine during surgery; MedSurgKateBolus_bin, administration of catecholamine during surgery; MinMAPcumu1MinAnes, minimal MAP for 1 minute during anesthesia; MinMAPcumu5MinAnes, minimal MAP for 5 minutes during anesthesia; NYHA, New York Heart Association classification of heart disease; RCRI, Revised Cardiac Risk Index.

Features	Group LASSO	LASSO	Ridge	Elastic Net	SVM	Linear xGBM	Tree xGBM	RFC
Urology surgery	100.00 (1)	100.00 (1)	100.00 (1)	100.00 (1)	32.65 (19)	23.14 (21)	21.69 (22)	18.67 (23)
eGFRPreSurg	34.98 (10)	23.25 (13)	25.70 (17)	23.42 (14)	84.31 (2)	100.00 (1)	100.00 (1)	100.00 (1)
Duration of anesthesia	45.56 (5)	27.21 (11)	27.10 (16)	30.94 (12)	44.15 (15)	87.02 (2)	62.68 (3)	76.98 (2)
General surgery	75.72 (2)	62.41 (2)	54.22 (8)	62.58 (2)	47.84 (11)	32.65 (16)	26.95 (18)	16.64 (24)
Neurology surgery	64.05 (3)	57.54 (3)	87.18 (2)	59.56 (3)	45.67 (13)	15.26 (25)	21.93 (21)	16.43 (25)
ASA	19.96 (20)	36.28 (6)	76.19 (4)	53.28 (4)	100.00 (1)	4.51 (40)	6.29 (35)	27.62 (21)
Age	21.15 (19)	10.24 (20)	17.57 (23)	13.66 (21)	45.12 (14)	57.32 (5)	65.82 (2)	64.16 (3)
Diuretics	53.13 (4)	36.91 (5)	44.81 (12)	39.31 (9)	63.03 (4)	16.40 (24)	17.11 (24)	12.95 (29)
doseInduPropoPerfu	27.09 (15)	11.37 (18)	19.00 (22)	15.39 (19)	77.06 (3)	33.88 (14)	56.29 (5)	35.77 (19)
doseSurgSteroInf	21.67 (17)	13.98 (16)	15.91 (28)	14.88 (20)	31.30 (20)	54.89 (6)	56.44 (4)	57.09 (5)
Mean	14.31 (28)	6.15 (28)	7.75 (39)	6.16 (33)	22.65 (26)	61.62 (4)	54.88 (6)	60.57 (4)
Surgical urgency	34.79 (11)	35.86 (7)	46.60 (11)	41.84 (6)	39.76 (16)	6.67 (34)	4.70 (37)	18.82 (22)
Standard deviation	3.22 (39)	1.31 (37)	6.12 (46)	4.32 (39)	26.81 (23)	70.15 (3)	43.76 (9)	56.72 (6)
doseInduSufenBolus	27.11 (14)	10.92 (19)	16.13 (27)	15.62 (18)	45.83 (12)	29.37 (18)	25.11 (19)	39.74 (18)
Thoracic surgery	37.48 (9)	34.15 (9)	77.99 (3)	41.82 (7)	10.95 (43)	1.19 (54)	1.27 (56)	1.88 (54)
Liver Cirrhosis	38.89 (8)	40.30 (4)	61.24 (6)	45.33 (5)	13.56 (37)	3.75 (41)	1.06 (58)	1.56 (56)
BMI	18.48 (21)	9.45 (23)	14.39 (32)	12.10 (25)	33.95 (18)	37.64 (11)	33.07 (16)	43.70 (14)
Duration of induction	8.47 (32)	0.39 (39)	1.74 (54)	4.39 (38)	38.29 (17)	47.30 (7)	47.68 (8)	51.98 (9)
aucMAPunder65Anes	6.54 (36)	0.00 (41)	1.57 (55)	0.46 (45)	53.70 (9)	37.19 (12)	50.06 (7)	50.25 (11)
Vascular surgery	40.23 (7)	33.50 (10)	68.80 (5)	40.55 (8)	5.31 (56)	3.25 (45)	2.81 (50)	2.25 (53)
doseInduPropoBolus	0.00 (47)	0.00 (41)	1.77 (53)	0.00 (46)	57.70 (6)	43.76 (9)	40.34 (12)	43.90 (13)
Biguanides	41.07 (6)	34.52 (8)	43.70 (13)	37.05 (10)	19.63 (30)	2.56 (48)	3.99 (42)	3.54 (49)
minMAPcumu5MinAnes	7.27 (35)	4.28 (32)	9.66 (36)	5.76 (34)	53.76 (8)	23.92 (20)	35.79 (15)	42.17 (17)
gender	29.03 (12)	20.71 (14)	29.69 (14)	21.93 (15)	47.84 (10)	10.35 (29)	10.43 (27)	7.91 (32)
Trend	8.76 (30)	2.32 (36)	6.64 (43)	4.64 (37)	21.40 (28)	43.04 (10)	37.22 (14)	52.01 (8)
Skewness	7.74 (34)	0.00 (41)	3.82 (48)	0.55 (44)	8.76 (48)	47.13 (8)	43.48 (10)	52.41 (7)
Cerebrovascular Disease	27.98 (13)	26.45 (12)	47.49 (10)	28.75 (13)	7.92 (51)	9.34 (30)	6.72 (33)	4.62 (39)
Entropy	18.21 (23)	9.38 (24)	15.69 (29)	12.51 (23)	19.26 (33)	30.21 (17)	19.24 (23)	33.65 (20)
minMAPcumu1MinAnes	0.00 (47)	0.00 (41)	1.22 (56)	0.00 (46)	55.70 (7)	22.91 (22)	23.48 (20)	43.27 (15)
Kurtosis	2.69 (40)	0.00 (41)	3.39 (49)	0.00 (46)	0.00 (61)	36.84 (13)	42.55 (11)	51.22 (10)
NYHA	8.61 (31)	18.16 (15)	59.60 (7)	32.18 (11)	12.09 (41)	0.53 (58)	0.48 (61)	3.87 (44)
baselineMAP	0.00 (47)	0.00 (41)	0.02 (61)	0.00 (46)	5.09 (57)	33.28 (15)	39.90 (13)	47.18 (12)
height	0.00 (47)	0.00 (41)	0.56 (58)	0.00 (46)	17.74 (34)	28.98 (19)	29.12 (17)	43.12 (16)
RCRI	6.14 (37)	0.94 (38)	17.15 (25)	6.22 (32)	59.70 (5)	2.22 (49)	3.85 (43)	15.95 (26)
doseInduSufenPerfu	21.56 (18)	12.62 (17)	19.27 (21)	13.14 (22)	27.44 (22)	2.61 (47)	6.73 (32)	7.02 (33)
doseInduRemifPerfu	22.36 (16)	4.63 (30)	8.76 (38)	11.21 (28)	24.67 (24)	8.30 (31)	15.03 (25)	15.34 (27)
Otolaryngology surgery	18.33 (22)	4.48 (31)	49.42 (9)	16.08 (17)	6.62 (54)	0.38 (60)	1.60 (54)	0.00 (61)
doseInduThiopBolus	13.76 (29)	6.22 (27)	9.02 (37)	7.18 (31)	23.11 (25)	13.42 (26)	8.28 (30)	12.20 (30)
Trauma surgery	16.77 (25)	2.77 (34)	28.79 (15)	8.59 (30)	6.62 (55)	13.04 (27)	7.43 (31)	4.18 (42)
doseSurgGelafInf	5.32 (38)	2.61 (35)	6.89 (42)	3.76 (40)	19.58 (32)	12.49 (28)	9.91 (28)	14.66 (28)
medInduKateBolus_bin	14.46 (27)	9.71 (22)	13.12 (35)	11.87 (26)	7.92 (53)	7.83 (32)	4.41 (39)	5.43 (36)

Features	Group LASSO	LASSO	Ridge	Elastic Net	SVM	Linear xGBM	Tree xGBM	RFC
Statins	15.66 (26)	5.59 (29)	14.15 (33)	10.70 (29)	10.52 (45)	6.31 (35)	6.58 (34)	5.10 (37)
Stomach tube	17.13 (24)	8.47 (25)	14.96 (30)	12.47 (24)	7.92 (52)	5.43 (36)	3.62 (44)	4.38 (40)
Chronic heart failure	2.62 (41)	9.85 (21)	24.64 (18)	19.25 (16)	11.39 (42)	0.91 (57)	0.67 (60)	0.73 (59)
maxSevoExp	8.14 (33)	0.10 (40)	3.20 (51)	2.91 (41)	22.30 (27)	7.41 (33)	11.47 (26)	11.37 (31)
Diabetes	0.41 (45)	2.93 (33)	19.61 (20)	5.15 (36)	20.50 (29)	4.53 (39)	3.11 (46)	3.67 (45)
Peripheral Arterial Disease	0.00 (47)	6.72 (26)	21.81 (19)	11.82 (27)	0.11 (59)	3.07 (46)	2.35 (52)	1.85 (55)
medSurgKateBolus_bin	0.00 (47)	0.00 (41)	2.20 (52)	0.63 (43)	12.69 (39)	16.70 (23)	9.49 (29)	5.62 (35)
Beta-blockers	0.00 (47)	0.00 (41)	4.29 (47)	0.00 (46)	27.44 (21)	5.38 (37)	4.69 (38)	4.05 (43)
Indwelling urinary catheter	1.40 (43)	0.00 (41)	7.31 (40)	0.00 (46)	19.63 (31)	3.47 (43)	5.23 (36)	4.81 (38)
Coronary Heart Disease	0.00 (47)	0.00 (41)	13.79 (34)	2.45 (42)	12.26 (40)	1.15 (55)	2.77 (51)	2.58 (51)
Other surgery department	0.38 (46)	0.00 (41)	16.30 (26)	0.00 (46)	8.78 (47)	1.53 (52)	2.89 (49)	3.61 (46)
Atrial fibrillation	0.00 (47)	0.00 (41)	14.71 (31)	5.34 (35)	8.78 (46)	1.73 (51)	1.26 (57)	1.46 (57)
doseInduEtomidateBolus	0.00 (47)	0.00 (41)	0.02 (60)	0.00 (46)	16.29 (35)	5.21 (38)	4.38 (40)	5.82 (34)
Calcium Channel Blockers	0.00 (47)	0.00 (41)	3.31 (50)	0.00 (46)	13.99 (36)	3.60 (42)	4.00 (41)	3.06 (50)
Sartans	0.00 (47)	0.00 (41)	1.18 (57)	0.00 (46)	13.12 (38)	1.79 (50)	3.10 (47)	3.59 (48)
Insulins	0.00 (47)	0.00 (41)	7.29 (41)	0.00 (46)	8.35 (49)	0.53 (59)	2.30 (53)	2.50 (52)
Plastic surgery	0.68 (44)	0.00 (41)	17.50 (24)	0.00 (46)	0.11 (60)	0.00 (61)	1.30 (55)	0.75 (58)
Cardiac surgery	1.63 (42)	0.00 (41)	6.35 (45)	0.00 (46)	8.35 (50)	1.49 (53)	0.84 (59)	0.58 (60)
ACE inhibitors	0.00 (47)	0.00 (41)	0.26 (59)	0.00 (46)	10.52 (44)	1.13 (56)	2.92 (48)	4.22 (41)
medSurgAtroBolus_bin	0.00 (47)	0.00 (41)	6.44 (44)	0.00 (46)	0.97 (58)	3.37 (44)	3.16 (45)	3.60 (47)

Table 6. Average Shapley values of model predictions on the validation data set by final PO-AKI prediction models. Shapley values were computed for all individual model predictions on the validation data set and then averaged to estimate overall feature contribution. Shown are the average Shapley values and their respective ranks (in parentheses) for each model. Features are ordered by their mean ranks across all models.
Abbrev.: age, patient age; ASA, American Society of Anesthesiology physical status; AUC, area under the curve; AucMAPunder65Anes, area under the Curve for all MAP below 65mmHg during anesthesia; BMI, body mass index of patient; DoseInduEtomidateBolus, dose of etomidate administered via bolus during induction; DoseInduPropoBolus, dose of propofol administered via bolus during induction; DoseInduPropoPerfu, dose of propofol administered via perfusor during induction DoseInduRemifPerfu, dose of remifentanil administered via perfusor duringinduction DoseInduSufenBolus, dose of sufentanil administered via bolus during induction; DoseInduSufenPerfu, dose of sufentanil administered via perfusor during induction; DoseInduThiopBolus, dose of thiopental administered via bolus during induction; DoseSurgGelafInf, dose of gelafundin administered via infusion during induction; DoseSurgSteroInf, dose of sterofundin administered via infusion during induction; EGFRPreSurg, estimated Glomerular Filtration Rate before surgery; height, patient height; MAP, mean arterial pressure; MaxSevoExp, maximum expiratory sevoflurane measurement during induction; MedInduKateBolus_bin, administration of catecholamine during induction; MedSurgAtroBolus_bin, administration of atropine during surgery; MedSurgKateBolus_bin, administration of catecholamine during surgery; MinMAPcumu1MinAnes, minimal MAP for 1 minute during anesthesia; MinMAPcumu5MinAnes, minimal MAP for 5 minutes during anesthesia; NYHA, New York Heart Association classification of heart disease; RCRI, Revised Cardiac Risk Index.

Features	Group LASSO	LASSO	Ridge	Elastic Net	SVM	Linear xGBM	Tree xGBM	RFC
General surgery	0.213 (3)	0.186 (1)	0.143 (2)	0.246 (1)	0.045 (1)	0.047 (3)	0.020 (3)	0.045 (1)
eGFRPreSurg	0.226 (2)	0.163 (2)	0.120 (3)	0.179 (4)	0.034 (4)	0.068 (1)	0.028 (1)	0.034 (4)
Duration of anesthesia	0.245 (1)	0.124 (4)	0.113 (4)	0.203 (3)	0.036 (2)	0.050 (2)	0.015 (4)	0.036 (2)
ASA	0.138 (7)	0.146 (3)	0.174 (1)	0.205 (2)	0.034 (3)	0.025 (9)	0.013 (5)	0.034 (3)
Diuretics	0.141 (5)	0.106 (5)	0.098 (7)	0.137 (6)	0.031 (6)	0.023 (10)	0.012 (6)	0.031 (6)
doseInduPropoPerfu	0.179 (4)	0.102 (6)	0.093 (8)	0.091 (11)	0.031 (5)	0.026 (8)	0.021 (2)	0.031 (5)
Urology surgery	0.067 (17)	0.096 (7)	0.102 (5)	0.158 (5)	0.024 (9)	0.023 (11)	0.007 (14)	0.024 (9)
gender	0.104 (9)	0.070 (9)	0.085 (9)	0.101 (8)	0.030 (7)	0.013 (22)	0.006 (16)	0.030 (7)
Neurology surgery	0.071 (16)	0.090 (8)	0.080 (10)	0.100 (9)	0.020 (11)	0.008 (29)	0.009 (10)	0.020 (11)
Surgical urgency	0.044 (21)	0.041 (12)	0.101 (6)	0.129 (7)	0.021 (10)	0.014 (20)	0.005 (20)	0.021 (10)
doseSurgSteroInf	0.111 (8)	0.043 (10)	0.063 (15)	0.094 (10)	0.012 (19)	0.0217 (12)	0.010 (8)	0.012 (19)
doseInduSufenBolus	0.140 (6)	0.028 (14)	0.065 (13)	0.072 (12)	0.017 (17)	0.016 (17)	0.004 (25)	0.017 (17)
Age	0.095 (13)	0.009 (20)	0.070 (12)	0.069 (13)	0.028 (8)	0.033 (4)	0.011 (7)	0.028 (8)
BMI	0.099 (11)	0.022 (17)	0.063 (14)	0.067 (14)	0.018 (14)	0.020 (13)	0.006 (15)	0.018 (14)
doseInduThiopBolus	0.100 (10)	0.028 (13)	0.043 (19)	0.048 (16)	0.015 (18)	0.006 (34)	0.001 (39)	0.015 (18)
Entropy	0.078 (14)	0.000 (53)	0.053 (16)	0.050 (15)	0.020 (12)	0.014 (21)	0.004 (24)	0.020 (12)
Mean	0.065 (18)	0.000 (56)	0.035 (24)	0.047 (17)	0.012 (21)	0.028 (6)	0.009 (9)	0.012 (21)
doseInduSufenPerfu	0.072 (15)	0.023 (16)	0.043 (18)	0.041 (19)	0.004 (44)	0.003 (43)	0.002 (37)	0.004 (44)
minMAPcumu5MinAnes	0.039 (22)	0.041 (11)	0.041 (20)	0.033 (23)	0.009 (26)	0.012 (24)	0.005 (19)	0.009 (26)
Biguanides	0.022 (29)	0.026 (15)	0.037 (21)	0.037 (21)	0.010 (24)	0.0058 (36)	0.002 (36)	0.010 (24)
doseInduRemifPerfu	0.097 (12)	0 (57)	0.021 (37)	0.016 (29)	0.004 (43)	0.004 (39)	0.002 (33)	0.004 (43)
medInduKateBolus_bin	0.032 (24)	0.000 (37)	0.032 (26)	0.038 (20)	0.017 (15)	0.007 (32)	0.002 (38)	0.017 (15)
RCRI	0.007 (38)	0.000 (25)	0.080 (11)	0.008 (32)	0.017 (16)	0.008 (28)	0.003 (26)	0.017 (16)
Standard deviation	0.027 (26)	0.000 (55)	0.025 (33)	0.006 (35)	0.020 (13)	0.029 (5)	0.005 (17)	0.020 (13)
Stomach tube	0.033 (23)	0.000 (38)	0.035 (23)	0.031 (24)	0.012 (20)	0.006 (35)	0.000 (51)	0.012 (20)
Trend	0.047 (20)	0.000 (47)	0.030 (28)	0.016 (30)	0.007 (28)	0.017 (15)	0.005 (18)	0.007 (28)
NYHA	0 (42)	0.008 (21)	0.048 (17)	0.044 (18)	0.007 (29)	0.008 (27)	0.003 (27)	0.007 (29)
doseSurgGelafInf	0.028 (25)	0.000 (54)	0.036 (22)	0.024 (27)	0.010 (25)	0.005 (37)	0.002 (32)	0.010 (25)
Liver Cirrhosis	0.008 (35)	0.013 (18)	0.028 (30)	0.036 (22)	0.007 (33)	0.004 (40)	0.000 (58)	0.007 (33)
Thoracic surgery	0.015 (33)	0.006 (23)	0.029 (29)	0.025 (26)	0.006 (36)	0.002 (54)	0.000 (48)	0.006 (36)
Cerebrovascular Disease	0.011 (34)	0.013 (19)	0.027 (31)	0.023 (28)	0.003 (50)	0.005 (38)	0.002 (35)	0.003 (50)
Vascular surgery	0.016 (32)	0.006 (22)	0.023 (35)	0.027 (25)	0.005 (37)	0.003 (45)	0.001 (44)	0.005 (37)
Statins	0.021 (30)	0.000 (46)	0.022 (36)	0.011 (31)	0.012 (22)	0.007 (31)	0.001 (40)	0.012 (22)
maxSevoExp	0.048 (19)	0.000 (51)	0.010 (45)	0.000 (54)	0.007 (27)	0.007 (33)	0.002 (31)	0.007 (27)
Duration of Induction	0.026 (28)	0.000 (60)	0.004 (54)	0.000 (56)	0.007 (32)	0.028 (7)	0.007 (13)	0.007 (32)
Trauma surgery	0.020 (31)	0.000 (44)	0.030 (27)	0.006 (34)	0.007 (31)	0.004 (41)	0.002 (34)	0.007 (31)
Skewness	0.027 (27)	0.000 (50)	0.014 (40)	0.000 (55)	0.006 (35)	0.017 (16)	0.004 (21)	0.006 (35)
Other surgery department	0.007 (36)	0.000 (41)	0.032 (25)	0.000 (53)	0.011 (23)	0.003 (49)	0.001 (61)	0.011 (23)
doseInduPropoBolus	0.000 (60)	0.001 (24)	0.018 (38)	0.000 (58)	0.004 (46)	0.014 (19)	0.007 (12)	0.004 (46)
aucMAPunder65Anes	0.007 (37)	0.000 (52)	0.000 (61)	0.000 (57)	0.003 (51)	0.020 (14)	0.008 (11)	0.003 (51)
Indwelling urinary catheter	0.000 (48)	0.000 (36)	0.023 (34)	0.000 (46)	0.007 (34)	0.003 (47)	0.001 (41)	0.007 (34)

Features	Group LASSO	LASSO	Ridge	Elastic Net	SVM	Linear xGBM	Tree xGBM	RFC
Diabetes	0.000 (54)	0.000 (39)	0.025 (32)	0.006 (36)	0.002 (58)	0.002 (52)	0.001 (47)	0.002 (58)
Kurtosis	0.000 (57)	0.000 (48)	0.010 (46)	0.000 (51)	0.003 (49)	0.015 (18)	0.004 (23)	0.003 (49)
minMAPcumu1MinAnes	0.000 (56)	0.000 (49)	0.011 (43)	0.000 (61)	0.002 (56)	0.011 (26)	0.003 (28)	0.002 (56)
Chronic heart failure	0.000 (43)	0.000 (26)	0.012 (42)	0.007 (33)	0.003 (54)	0.002 (51)	0.001 (60)	0.003 (54)
baselineMAP	0.000 (59)	0.000 (58)	0.002 (57)	0.000 (60)	0.003 (47)	0.013 (23)	0.004 (22)	0.003 (47)
Otolaryngology surgery	0.004 (39)	0.000 (43)	0.010 (47)	0.002 (38)	0.004 (41)	0.002 (59)	0.001 (50)	0.004 (41)
medSurgKateBolus_bin	0.000 (53)	0.000 (33)	0.006 (50)	0.000 (48)	0.005 (38)	0.008 (30)	0.002 (30)	0.005 (38)
Beta-blockers	0.000 (44)	0.000 (30)	0.013 (41)	0.000 (40)	0.004 (42)	0.004 (42)	0.001 (43)	0.004 (42)
Height	0.000 (58)	0.000 (59)	0.004 (53)	0.000 (59)	0.003 (52)	0.012 (25)	0.003 (29)	0.003 (52)
Coronary Heart Disease	0.000 (50)	0.000 (27)	0.014 (39)	0.000 (45)	0.004 (45)	0.002 (58)	0.001 (53)	0.004 (45)
Peripheral Arterial Disease	0.000 (46)	0.000 (34)	0.011 (44)	0.004 (37)	0.002 (59)	0.003 (50)	0.001 (45)	0.002 (59)
Atrial fibrillation	0.000 (47)	0.000 (28)	0.009 (48)	0.000 (42)	0.005 (39)	0.002 (56)	0.001 (49)	0.005 (39)
doseInduEtomidateBolus	0.000 (61)	0.000 (61)	0.001 (59)	0.000 (52)	0.007 (30)	0.003 (48)	0.001 (42)	0.007 (30)
Calcium channel blockers	0.000 (45)	0.000 (29)	0.008 (49)	0.000 (39)	0.002 (60)	0.003 (44)	0.001 (52)	0.002 (60)
Sartans	0.000 (49)	0.000 (32)	0.003 (55)	0.000 (47)	0.004 (40)	0.003 (46)	0.001 (56)	0.004 (40)
Cardiac surgery	0.001 (40)	0.000 (42)	0.002 (56)	0.000 (44)	0.003 (48)	0.002 (55)	0.001 (59)	0.003 (48)
medSurgAtroBolus_bin	0.000 (51)	0.000 (45)	0.005 (51)	0.000 (49)	0.003 (55)	0.002 (57)	0.001 (46)	0.003 (55)
Plastic surgery	0.000 (41)	0.000 (40)	0.004 (52)	0.000 (50)	0.001 (61)	0.002 (61)	0.001 (54)	0.001 (61)
ACE inhibitors	0.000 (55)	0.000 (31)	0.001 (60)	0.000 (41)	0.003 (53)	0.002 (53)	0.001 (55)	0.003 (53)
Insulins	0.000 (52)	0.000 (35)	0.002 (58)	0.000 (43)	0.002 (57)	0.002 (60)	0.001 (57)	0.002 (57)

Table 7. Performance of different AKI severity stage-specific RFC models. Mean and standard deviation of different performance metrics, estimated over 100 bootstrapping iterations of the respective AKI stage-specific validation data sets. Outcome distributions (No AKI/AKI) are provided for development (N dev) and validation data set (N val).
Abbrev.: Neg Pred Value, negative predictive value; RFC, Random Forest Classifier; ROC-AUC, area under receiver operating characteristics curve.

Data	N dev	N val	ROC-AUC	Sensitivity	Specificity	Neg Pred Value	Precision	F1	Balanced Accuracy
AKI 1	(802/802)	(4,055/406)	0.710 ± 0.013	0.648 ± 0.021	0.648 ± 0.007	0.948 ± 0.004	0.156 ± 0.008	0.251 ± 0.012	0.648 ± 0.011
AKI 2	(181/181)	(4,055/100)	0.744 ± 0.023	0.660 ± 0.047	0.684 ± 0.008	0.988 ± 0.002	0.048 ± 0.006	0.090 ± 0.010	0.672 ± 0.023
AKI 3	(160/160)	(4,055/69)	0.894 ± 0.024	0.872 ± 0.047	0.792 ± 0.007	0.997 ± 0.001	0.068 ± 0.007	0.126 ± 0.012	0.832 ± 0.024

Table 8. Performance of RFC models trained on the balanced development cohort with and without preoperative eGFR. Mean and standard deviation of different performance metrics, estimated over 100 bootstrapping iterations of the respective validation data sets.
Abbrev.: Neg Pred Value, negative predictive value; RFC, Random Forest Classifier; ROC-AUC, area under receiver operating characteristics curve.

Data	AUC	Sensitivity	Specificity	Neg Pred Value	Precision	F1	Balanced Accuracy
Full Model	0.738 ± 0.011	0.740 ± 0.019	0.600 ± 0.008	0.942 ± 0.005	0.209 ± 0.009	0.326 ± 0.012	0.670 ± 0.010
no eGFR	0.730 ± 0.010	0.653 ± 0.019	0.661 ± 0.007	0.930 ± 0.004	0.215 ± 0.010	0.324 ± 0.012	0.657 ± 0.010

Table 9. Final model performances of Random Forest classifier models, trained on the balanced development cohort at different time points during anesthesia. Mean and standard deviation of different performance metrics, estimated on 100 different bootstrap samples of the corresponding validation datasets to estimate overall performance variances are provided.
Abbrev.: BACC, Balanced Accuracy; Neg Pred Value, negative predictive value; ROC-AUC, area under receiver operating characteristics curve.

Model	ROC-AUC	Sensitivity	Specificity	Neg Pred Value	Precision	F1-score	BACC
Preoperative	0.722 ± 0.012	0.704 ± 0.021	0.617 ± 0.007	0.936 ± 0.005	0.208 ± 0.009	0.321 ± 0.012	0.661 ± 0.011
Induction	0.720 ± 0.010	0.722 ± 0.016	0.596 ± 0.009	0.938 ± 0.004	0.203 ± 0.010	0.317 ± 0.013	0.659 ± 0.010
Induction + 30 min	0.726 ± 0.012	0.728 ± 0.018	0.602 ± 0.007	0.939 ± 0.004	0.208 ± 0.010	0.323 ± 0.013	0.665 ± 0.010
Induction + 60 min	0.725 ± 0.010	0.723 ± 0.019	0.604 ± 0.008	0.939 ± 0.004	0.207 ± 0.010	0.322 ± 0.012	0.664 ± 0.010
Induction + 90 min	0.725 ± 0.011	0.727 ± 0.020	0.600 ± 0.008	0.939 ± 0.005	0.207 ± 0.009	0.322 ± 0.012	0.663 ± 0.011
Induction + 120 min	0.722 ± 0.010	0.711 ± 0.018	0.619 ± 0.009	0.938 ± 0.004	0.210 ± 0.009	0.325 ± 0.012	0.665 ± 0.010
Induction + 150 min	0.716 ± 0.011	0.710 ± 0.019	0.621 ± 0.008	0.938 ± 0.004	0.210 ± 0.010	0.324 ± 0.014	0.666 ± 0.010
Postoperative	0.738 ± 0.011	0.740 ± 0.019	0.600 ± 0.008	0.942 ± 0.005	0.209 ± 0.009	0.326 ± 0.012	0.670 ± 0.010

Table 10. Native Feature Importances (FI) of Random Forest classifiers (RFCs) trained on the balanced development cohort at different perioperative time points. For each defined time point, the FI was extracted from the corresponding RFC. Scaled native FI values and their respective ranks (in parentheses) for each time point are shown. Lower rank values (1: highest rank) indicate higher importance. To facilitate comparability across time points, time point specific variables are indicated by the label "TP". Features are ordered by the sum of their ranks across all time points. FIs of variables that were not available at certain time points were set to 0. Abbrev.: age, patient age; ASA, American Society of Anesthesiology physical status; AUC, area under the curve; AucMAPunder65Anes, area under the Curve for all MAP below 65mmHg during anesthesia; BMI, body mass index of patient; DoseInduEtomidateBolus, dose of etomidate administered via bolus during induction; DoseInduPropoBolus, dose of propofol administered via bolus during induction; DoseInduPropoPerfu, dose of propofol administered via perfusor during induction DoseInduRemifPerfu, dose of remifentanil administered via perfusor duringinduction DoseInduSufenBolus, dose of sufentanil administered via bolus during induction; DoseInduSufenPerfu, dose of sufentanil administered via perfusor during induction; DoseInduThiopBolus, dose of thiopental administered via bolus during induction; DoseSurgGelafInf, dose of gelafundin administered via infusion during induction; DoseSurgSteroInf, dose of sterofundin administered via infusion during induction; EGFRPreSurg, estimated Glomerular Filtration Rate before surgery; height, patient height; MAP, mean arterial pressure; MaxSevoExp, maximum expiratory sevoflurane measurement during induction; MedInduKateBolus_bin, administration of catecholamine during induction; MedSurgAtroBolus_bin, administration of atropine during surgery; MedSurgKateBolus_bin, administration of catecholamine during surgery; MinMAPcumu1MinAnes, minimal MAP for 1 minute during anesthesia; MinMAPcumu5MinAnes, minimal MAP for 5 minutes during anesthesia; NYHA, New York Heart Association classification of heart disease; RCRI, Revised Cardiac Risk Index.

Variable	Pre-operative	Induction	Induction + 30 min	Induction + 60 min	Induction + 90 min	Induction + 120 min	Induction + 150 min	Post-operative
eGFRPreSurg	100.00 (1)	100.00 (1)	100.00 (1)	100.00 (1)	100.00 (1)	100.00 (1)	100.00 (1)	100.00 (1)
Age	80.84 (2)	74.03 (2)	71.88 (2)	67.83 (2)	72.58 (2)	67.80 (2)	67.54 (2)	72.58 (3)
Standard Deviation (TP)	0.00 (37)	64.37 (4)	66.03 (3)	60.16 (3)	65.92 (3)	62.81 (3)	55.65 (3)	66.57 (6)
Mean (TP)	0.00 (37)	65.18 (3)	63.75 (4)	58.47 (5)	64.05 (5)	61.05 (4)	55.44 (4)	70.05 (4)
baselineMAP	72.76 (3)	58.16 (11)	55.82 (9)	49.07 (10)	56.09 (10)	50.91 (10)	43.14 (11)	59.35 (12)
Duration of induction	0.00 (37)	63.87 (6)	63.24 (5)	59.60 (4)	65.10 (4)	58.43 (6)	52.49 (6)	63.80 (10)
Skewness (TP)	0.00 (37)	64.12 (5)	60.17 (7)	55.57 (7)	63.17 (6)	54.91 (7)	51.83 (7)	64.45 (8)
Trend (TP)	0.00 (37)	62.68 (7)	58.70 (8)	58.02 (6)	63.03 (7)	59.14 (5)	52.89 (5)	61.27 (11)
Kurtosis (TP)	0.00 (37)	60.75 (9)	60.60 (6)	51.92 (8)	61.98 (8)	54.48 (8)	48.16 (8)	64.45 (7)
BMI	64.56 (4)	55.87 (15)	52.41 (12)	48.58 (11)	54.30 (13)	50.22 (11)	43.42 (10)	55.81 (16)
height	63.71 (5)	56.32 (13)	51.69 (13)	45.23 (15)	53.86 (14)	47.53 (14)	41.76 (12)	53.37 (18)
aucMAPunder65 (TP)	0.00 (37)	38.77 (18)	50.69 (15)	49.20 (9)	58.47 (9)	52.13 (9)	44.82 (9)	64.30 (9)
doseInduPropo(Bolus)	0.00 (37)	56.70 (12)	54.92 (10)	48.31 (12)	54.45 (12)	48.30 (13)	40.64 (14)	57.29 (14)
minMAPcumu1Min (TP)	0.00 (37)	58.88 (10)	51.68 (14)	47.89 (13)	55.37 (11)	49.62 (12)	39.02 (15)	58.14 (13)
doseInduSufen(bolus)	0.00 (37)	56.10 (14)	53.15 (11)	47.81 (14)	52.95 (15)	45.72 (16)	37.80 (16)	53.47 (17)
minMAPcumu5Min (TP)	0.00 (37)	61.37 (8)	50.53 (16)	42.98 (16)	52.77 (16)	46.14 (15)	37.29 (17)	56.16 (15)
ASA	32.41 (6)	33.34 (19)	35.24 (19)	27.22 (19)	32.94 (19)	29.05 (19)	26.48 (19)	34.17 (21)
Surgical urgency	23.66 (7)	20.06 (21)	22.55 (20)	21.01 (20)	23.01 (20)	20.40 (20)	21.62 (21)	21.24 (23)
doseInduPropo(Perfu)	0.00 (37)	42.36 (17)	49.94 (17)	39.56 (17)	43.61 (17)	39.66 (17)	40.74 (13)	44.51 (19)
RCRI	22.49 (8)	22.70 (20)	22.45 (21)	16.86 (24)	22.80 (21)	17.69 (22)	14.29 (24)	21.91 (22)
Entropy (TP)	0.00 (37)	52.56 (16)	43.98 (18)	36.98 (18)	43.44 (18)	38.43 (18)	29.23 (18)	44.25 (20)
Urology surgery	13.66 (11)	16.94 (26)	20.71 (23)	20.11 (21)	19.45 (22)	20.08 (21)	21.77 (20)	18.61 (26)
Neurology Surgery	16.69 (9)	16.25 (27)	20.77 (22)	17.78 (22)	18.79 (24)	17.62 (23)	15.56 (23)	17.72 (29)
General surgery	14.08 (10)	17.51 (24)	18.74 (24)	17.53 (23)	17.62 (26)	16.15 (24)	17.68 (22)	18.38 (28)
doseInduRemif(Perfu)	0.00 (37)	17.56 (23)	18.52 (26)	15.71 (25)	18.85 (23)	15.45 (25)	13.28 (25)	20.26 (24)
Diuretics	13.47 (12)	15.77 (28)	16.62 (28)	13.68 (27)	16.57 (28)	14.67 (27)	10.82 (27)	16.38 (31)
doseInduThiop(Bolus)	0.00 (37)	18.35 (22)	18.57 (25)	14.67 (26)	18.26 (25)	14.79 (26)	13.04 (26)	17.66 (30)
gender	11.43 (14)	12.19 (29)	11.55 (29)	8.74 (29)	11.83 (29)	8.66 (30)	5.61 (31)	12.08 (33)
maxSevoExp	0.00 (37)	17.44 (25)	16.68 (27)	12.34 (28)	17.36 (27)	13.67 (28)	9.98 (28)	18.42 (27)
Indwelling urinary catheter	13.12 (13)	8.92 (32)	7.15 (33)	5.75 (33)	8.34 (32)	6.07 (33)	3.87 (34)	7.91 (37)
doseInduSufen(Perfu)	0.00 (37)	9.61 (31)	10.37 (30)	8.44 (30)	11.49 (30)	8.82 (29)	6.91 (29)	12.25 (32)
doseInduEtomidate (Bolus)	0.00 (37)	8.66 (33)	8.98 (31)	6.95 (31)	8.13 (33)	6.61 (31)	6.05 (30)	7.97 (36)
medInduKate(Bolus_bin)	0.00 (37)	9.98 (30)	7.72 (32)	5.78 (32)	8.66 (31)	6.15 (32)	3.83 (35)	9.12 (35)
Beta-blockers	10.49 (16)	8.41 (34)	6.71 (34)	4.65 (37)	7.63 (34)	4.93 (37)	3.28 (37)	7.47 (39)

Variable	Pre-operative	Induction	Induction + 30 min	Induction + 60 min	Induction + 90 min	Induction + 120 min	Induction + 150 min	Post-operative
Stomach tube	10.52 (15)	7.81 (35)	5.91 (39)	4.42 (38)	7.45 (35)	5.20 (35)	3.14 (39)	7.88 (38)
Statins	8.03 (19)	6.60 (38)	6.47 (35)	5.15 (36)	6.46 (39)	4.95 (36)	3.70 (36)	6.92 (42)
NYHA	6.80 (21)	6.11 (40)	6.00 (38)	5.32 (34)	6.56 (38)	5.37 (34)	5.07 (32)	5.25 (45)
ACE inhibitors	9.62 (17)	7.05 (37)	6.42 (36)	4.38 (39)	6.65 (37)	4.12 (41)	2.95 (40)	7.10 (41)
Sartans	8.82 (18)	7.69 (36)	6.20 (37)	4.36 (40)	6.67 (36)	4.48 (38)	2.93 (41)	6.69 (43)
Other surgery department	7.39 (20)	6.37 (39)	5.44 (41)	3.59 (41)	6.26 (40)	4.26 (39)	2.49 (43)	7.17 (40)
Cerebrovascular Disease	4.90 (24)	5.33 (41)	5.69 (40)	5.22 (35)	4.82 (43)	4.26 (40)	4.76 (33)	5.02 (47)
Diabetes	5.03 (23)	4.92 (43)	5.15 (42)	3.44 (42)	5.39 (42)	4.11 (42)	2.83 (42)	4.50 (48)
Calcium channel blockers	6.48 (22)	5.14 (42)	4.34 (43)	3.21 (44)	5.41 (41)	3.35 (45)	2.42 (44)	5.10 (46)
Biguanides	3.96 (27)	3.54 (46)	3.90 (45)	3.43 (43)	4.73 (44)	3.73 (43)	3.16 (38)	3.81 (49)
Trauma surgery	4.64 (25)	4.78 (44)	3.98 (44)	3.16 (45)	4.00 (45)	3.37 (44)	2.38 (45)	5.56 (44)
Coronary Heart Disease	4.39 (26)	3.83 (45)	3.33 (47)	2.73 (46)	3.49 (46)	2.62 (47)	2.22 (47)	3.32 (50)
Thoracic surgery	2.19 (32)	2.60 (47)	3.63 (46)	2.46 (47)	3.14 (47)	3.06 (46)	2.23 (46)	2.58 (52)
Insulins	3.32 (28)	2.49 (48)	2.59 (49)	2.20 (48)	2.98 (48)	1.96 (49)	2.17 (49)	2.25 (54)
Duration of anesthesia	0.00 (37)	0.00 (56)	0.00 (56)	0.00 (56)	0.00 (56)	0.00 (56)	0.00 (56)	79.81 (2)
doseSurgSteroInf	0.00 (37)	0.00 (56)	0.00 (56)	0.00 (56)	0.00 (56)	0.00 (56)	0.00 (56)	66.89 (5)
Liver Cirrhosis	2.71 (30)	1.80 (50)	2.64 (48)	1.83 (50)	2.73 (49)	2.10 (48)	2.20 (48)	2.08 (56)
Atrial fibrillation	2.75 (29)	2.17 (49)	1.86 (51)	1.84 (49)	2.44 (51)	1.70 (51)	1.84 (52)	2.16 (55)
Vascular surgery	1.65 (35)	1.25 (52)	1.94 (50)	1.70 (52)	2.52 (50)	1.95 (50)	2.12 (51)	2.28 (53)
doseSurgGelafInf	0.00 (37)	0.00 (56)	0.00 (56)	0.00 (56)	0.00 (56)	0.00 (56)	0.00 (56)	19.61 (25)
Peripheral Arterial Disease	2.17 (33)	1.30 (51)	1.76 (52)	1.80 (51)	2.01 (52)	1.48 (52)	2.13 (50)	1.75 (57)
medSurgKateBolus_bin	0.00 (37)	0.00 (56)	0.00 (56)	0.00 (56)	0.00 (56)	0.00 (56)	0.00 (56)	10.13 (34)
Chronic heart failure	1.80 (34)	0.55 (54)	0.90 (54)	0.89 (53)	1.13 (54)	1.06 (53)	1.02 (53)	1.29 (58)
Cardiac surgery	2.62 (31)	0.68 (53)	1.28 (53)	0.13 (55)	1.37 (53)	0.79 (54)	0.63 (55)	0.93 (59)
medSurgAtroBolus_bin	0.00 (37)	0.00 (56)	0.00 (56)	0.00 (56)	0.00 (56)	0.00 (56)	0.00 (56)	3.31 (51)
Plastic surgery	0.00 (37)	0.00 (56)	0.00 (56)	0.21 (54)	0.00 (56)	0.18 (55)	0.66 (54)	0.19 (60)
Otolaryngology surgery	0.43 (36)	0.09 (55)	0.34 (55)	0.00 (56)	0.68 (55)	0.00 (56)	0.00 (56)	0.00 (61)

Table 11. Estimation of performance variance dependent on available predictor variable sets across different perioperative time points.
Mean and standard deviation of different performance metrics, estimated in 100 different splits of the respective development data set into training and test data sets to estimate training-set-dependent performance variance of a Random Forest classifier. Abbrev.: BACC, Balanced Accuracy; Neg Pred Value, negative predictive value; ROC-AUC, area under receiver operating characteristics curve.

Timepoint	ROC-AUC	Sensitivity	Specificity	Neg Pred Value	Precision	F1-score	BACC
Preoperative	0.732 ± 0.022	0.698 ± 0.037	0.629 ± 0.033	0.675 ± 0.026	0.654 ± 0.021	0.675 ± 0.023	0.663 ± 0.021
Induction	0.722 ± 0.023	0.676 ± 0.035	0.639 ± 0.033	0.664 ± 0.024	0.654 ± 0.022	0.664 ± 0.023	0.658 ± 0.021
Induction + 30 min	0.725 ± 0.024	0.681 ± 0.037	0.644 ± 0.034	0.669 ± 0.027	0.658 ± 0.024	0.669 ± 0.026	0.663 ± 0.024
Induction + 60 min	0.733 ± 0.023	0.689 ± 0.034	0.645 ± 0.035	0.674 ± 0.025	0.661 ± 0.023	0.675 ± 0.023	0.667 ± 0.022
Induction + 90 min	0.656 ± 0.028	0.339 ± 0.081	0.827 ± 0.046	0.556 ± 0.021	0.663 ± 0.038	0.443 ± 0.076	0.583 ± 0.027
Induction + 120 min	0.738 ± 0.022	0.684 ± 0.033	0.651 ± 0.034	0.673 ± 0.024	0.664 ± 0.023	0.673 ± 0.022	0.668 ± 0.021
Induction + 150 min	0.737 ± 0.024	0.681 ± 0.033	0.652 ± 0.032	0.671 ± 0.024	0.663 ± 0.022	0.672 ± 0.023	0.667 ± 0.022
Postoperative	0.742 ± 0.023	0.688 ± 0.035	0.664 ± 0.035	0.680 ± 0.026	0.673 ± 0.024	0.680 ± 0.025	0.676 ± 0.024

References

1. Hackett, N. J., De Oliveira, G. S., Jain, U. K. & Kim, J. Y. Asa class is a reliable independent predictor of medical complications and mortality following surgery. *Int. J. Surg.* **18**, 184–190 (2015).
2. Shao, J. & Zhong, B. Last observation carry-forward and last observation analysis. *Stat. Medicine* **22**, 2429–2441 (2003).
3. Group, K. *et al.* Kdigo clinical practice guideline for acute kidney injury. *Kidney Int. Suppl.* **2**, 1 (2012).
4. Walsh, M. *et al.* Relationship between intraoperative mean arterial pressure and clinical outcomes after noncardiac surgery: Toward an empirical definition of hypotension. *Anesthesiology* **119**, 507–515 (2013).
5. Sun, L. Y., Wijeysundera, D. N., Tait, G. A. & Beattie, W. S. Association of intraoperative hypotension with acute kidney injury after elective noncardiac surgery. *Anesthesiology* **123**, 515–523 (2015).
6. Xue, B. *et al.* Use of machine learning to develop and evaluate models using preoperative and intraoperative data to identify risks of postoperative complications. *JAMA network open* **4**, e212240–e212240 (2021).
7. Shannon, C. E. A mathematical theory of communication. *Bell Syst. Tech. J.* **27**, 379–423 (1948).
8. Pearson, K. X. contributions to the mathematical theory of evolution.—ii. skew variation in homogeneous material. *Philos. Transactions Royal Soc. London. (A.)* **186**, 343–414 (1895).
9. Westfall, P. H. Kurtosis as peakedness, 1905–2014. r.i.p. *The Am. Stat.* **68**, 191–195 (2014).
10. Kendall, M. G. A new measure of rank correlation. *Biometrika* **30**, 81–93 (1938).
11. El-Shaarawi, A. H. & Niculescu, S. P. On kendall's tau as a test of trend in time series data. *Environmetrics* **3**, 385–411 (1992).
12. Kuhn, M. Building predictive models in r using the caret package. *J. Stat. Softw.* **28**, 1–26 (2008).
13. Gevrey, M., Dimopoulos, I. & Lek, S. Review and comparison of methods to study the contribution of variables in artificial neural network models. *Ecol. Model.* **160**, 249–264 (2003).
14. Louppe, G. *Understanding random forests: From theory to practice* (Universite de Liege (Belgium), 2014).
